# The Impacts of Benefit Sanctions: A Scoping Review of the Quantitative Research Evidence

**DOI:** 10.1017/S0047279421001069

**Published:** 2022-02-14

**Authors:** Serena Pattaro, Nick Bailey, Evan Williams, Marcia Gibson, Valerie Wells, Mark Tranmer, Chris Dibben

**Affiliations:** * Scottish Centre for Administrative Data Research, School of Social and Political Sciences, University of Glasgow; Urban Big Data Centre, 7 Lilybank Gardens, Glasgow G12 8RZ, UK; ** Scottish Centre for Administrative Data Research, School of Social and Political Sciences, University of Glasgow; Urban Big Data Centre, 7 Lilybank Gardens, Glasgow G12 8RZ, UK; *** Scottish Centre for Administrative Data Research, School of Social and Political Sciences, University of Glasgow; Urban Big Data Centre, 7 Lilybank Gardens, Glasgow G12 8RZ, UK; **** MRC/CSO Social and Public Health Sciences Unit, University of Glasgow, Berkeley Square, 99 Berkeley Street, Glasgow G3 7HR, UK; ***** MRC/CSO Social and Public Health Sciences Unit, University of Glasgow, Berkeley Square, 99 Berkeley Street, Glasgow G3 7HR, UK; ****** School of Social and Political Sciences, University of Glasgow; Urban Big Data Centre, 7 Lilybank Gardens, Glasgow G12 8RZ, UK; ******* Scottish Centre for Administrative Data Research, Institute of Geography and the Lived Environment, School of Geosciences, University of Edinburgh, Drummond Street, Edinburgh EH8 9XP, UK

**Keywords:** Benefit sanctions, Welfare conditionality, Job search, Unemployment, Social and health impacts, Scoping review

## Abstract

In recent decades, the use of conditionality backed by benefit sanctions for those claiming unemployment and related benefits has become widespread in the social security systems of high-income countries. Critics argue that sanctions may be ineffective in bringing people back to employment or indeed harmful in a range of ways. Existing reviews largely assess the labour market impacts of sanctions but our understanding of the wider impacts is more limited. We report results from a scoping review of the international quantitative research evidence on both labour market and wider impacts of benefit sanctions. Following systematic search and screening, we extract data for 94 studies reporting on 253 outcome measures. We provide a narrative summary, paying attention to the ability of the studies to support causal inference. Despite variation in the evidence base and study designs, we found that labour market studies, covering two thirds of our sample, consistently reported positive impacts for employment but negative impacts for job quality and stability in the longer term, along with increased transitions to non-employment or economic inactivity. Although largely relying on non-experimental designs, wider-outcome studies reported significant associations with increased material hardship and health problems. There was also some evidence that sanctions were associated with increased child maltreatment and poorer child well-being. Lastly, the review highlights the generally poor quality of the evidence base in this area, with few studies employing research methods designed to identify the causal impact of sanctions, especially in relation to wider impacts.

## 1 Introduction

Over the last three decades, social security systems in high-income countries have increasingly been marked by a tightening of eligibility requirements and the introduction of more conditions linked to the receipt of unemployment and related benefits (Langenbucher, 2015; Immervoll and Knotz, 2018). Conditions are enforced through the imposition of sanctions, which are a temporary reduction or interruption of benefit payments (that in some cases can be permanent) (Griggs and Evans, 2010). Introduced as part of a broader shift towards active labour market policies, sanctions are intended to promote compliance with conditions on work search or similar activities, and hence speed the return to employment (Eichhorst *et al*., 2008; Bonoli, 2010). Greater intensity in sanctions has been accompanied by an extension in the coverage of sanctions in some cases, so sanctions increasingly affect not only those unemployed but also those economically inactive or in low-paid work, and groups including lone parents and even those with a chronic illness or disability (Baumberg Geiger, 2017; Dwyer and Wright, 2014; McHale *et al*., 2020).

The impacts of sanction regimes are contested, but systematic reviews of the evidence are lacking. On the labour market side, there are numerous reviews and meta-analyses of evidence on active labour market policies. However, most of these have methodological limitations, including poor reporting of methods and lack of detail or non-systematic approaches to study identification and inclusion (e.g. McVicar, 2020). Reviews of active labour market policies shed little light on the impact of sanctions as they employ a typology of policies which places sanctioning in the category of job-search assistance (e.g. Kluve, 2010; Filges *et al*., 2015; Crépon and van den Berg, 2016; Card *et al*., 2018; Vooren *et al*., 2019; Yeyati *et al*., 2019). Very broadly, these studies suggest significant effects in terms of an increase in rates of both benefit exit and job entry in the short term. Others suggest that, in the longer term, there may be higher risks of economic inactivity or a return to unemployment benefits, and worsening job quality.

The literature on wider impacts is much smaller and has never been systematically identified or reviewed to our knowledge. We are aware of one scoping review of the health effects of participation in active labour market programmes, but it too includes sanctions within a wider category of job-search assistance (Puig-Barrachina *et al*., 2019). One review of UK welfare reformstudies includes quantitative and qualitative evidence on the wider impacts of sanctions, finding negative effects on a range of health and social outcomes (Hudson-Sharp *et al*., 2018). Individual studies frequently emphasise the negative consequences of sanctions for areas including financial stress and debt accumulation, adverse physical and mental health outcomes, hunger and utility cutoffs, increased reliance on food banks, survival crime, rent arrears, eviction and homelessness. Benefit sanctions are also reported to have negative repercussions for family relations, including impacts on the well-being of children, their cognitive development and education (Griggs and Evans, 2010; Watts *et al*., 2014; Dwyer, 2018; Webster, 2019). Further criticism of sanctions policies come from studies that have shown that they lead to the diversion of limited resources by key public services to address these consequences (National Audit Office, 2016a). Adverse resource impacts have also been highlighted by employers, who must deal with the large numbers of unsuitable job applications that arise from mandatory job-search requirements (Ingold, 2020).

In this study, we therefore provide a new assessment of the impact of sanctions by conducting a scoping review of the quantitative research evidence covering labour market and wider impacts. A scoping review involves the application of systematic search, screening and data extraction processes to identify and summarise evidence from the body of work in a given field. This provides transparency and minimises the scope for reviewer selection of works to bias findings.

The focus on quantitative evidence is not to deny the enormous importance of qualitative evidence in the study of welfare reform in general or the impact of conditionality or sanctions in particular. A great deal of that work informs the present review, both in terms of overall framing and in terms of thinking about the causal pathways or mechanisms by which sanctions produce outcomes or impacts. We draw on that wider literature in summarising our understanding of the latter. We have chosen to focus on quantitative studies, however, because they provide some measure of the direction and scale of impacts using the conventions of statistical methods, and offer a basis for comparison and synthesis (although we do not seek to provide a full meta-analysis here). Even with this limitation, the scale of this review is substantial.

Within the quantitative literature, we pay particular attention to research design, using the familiar concept of the hierarchy of evidence which runs from purely observational studies through those with quasi-experimental designs to randomised experiments (Guyatt *et al*., 1995). Unlike purely observational studies, the latter can provide more convincing evidence that observed relationships are causal, i.e. that it is sanction events which lead to particular outcomes rather than other factors. The idea of an evidence hierarchy has been challenged particularly by those who emphasise external validity or generalisability, and hence the need to build knowledge about causal mechanisms and the role of context in shaping outcomes (Deaton, 2010). Since our focus here is primarily on internal validity or assessing whether a particular programme had a particular outcome, the hierarchy remains a valid and useful framework (Imbens, 2010).

In this study, the primary focus is the working-age population in receipt of out-of-work benefits, such as unemployment-related or other means-tested benefits, which are subject to job-search and related requirements. By applying a rigorous systematic search strategy, study selection and data extraction process, we aim to provide a synthesis of the quantitative evidence base by capturing characteristics such as temporal and/or geographic spread, target population, research study design, outcomes assessed and results. We address the following research questions:

1. What is the scale and nature of the quantitative evidence base on the impacts of benefit sanctions, for both labour market and wider outcomes? How does this vary in terms of the study designs used?
2. What does the quantitative evidence suggest are the impacts of sanctions for both labour market and wider outcomes? Do conclusions vary depending on study design?

The remainder of this article is structured as follows. Section 2 briefly reviews the international contexts for sanction policies. Section 3 offers an overview of the various mechanisms for understanding the impacts of benefit sanctions. Section 4 presents the research design typology or hierarchy. Section 5 describes the methods used in the scoping review to identify and assess the evidence base. Results are presented in Section 6 which examines the scale and nature of the quantitative evidence, and Section 7 which summarises the results from this work in relation to labour market and wider outcomes. The article concludes with a summary (Section 8) and reflections on literature gaps, future research directions and policy implications.

## 2 Contexts for sanction policies

While benefit sanctions have long been a feature of some social security systems, in recent decades their severity in terms of value, duration, and requirements has markedly increased. Beginning with the Personal Responsibility Work Opportunity and Reconciliation Act (PRWORA) in the USA (1996), which escalated sanctioning for lone parents in receipt of means-tested benefits, many high-income countries have implemented increasingly stringent sanctioning regimes. These reforms were underpinned initially by arguments about work disincentives and benefit dependency produced by ‘passive’ welfare systems (Grubb, 2000), but given additional impetus by austerity policies introduced after the Global Financial Crisis (Moffitt, 2014). Such policies have focused primarily on supply-side factors, paying little attention to the impact of demand on individuals’ ability to find employment (Blank, 2002). Simultaneously, the labour market in many high-income countries has become increasingly flexibilised and precarious for many workers (Barbieri, 2009; Weber *et al*., 2020).

There are important contextual differences between sanction regimes in high-income countries which are likely to influence the impacts of sanctioning, and which have a bearing on interpretation of the evidence as a result. Table 1 provides the main characteristics of both social and unemployment protection policies, and sanction policies operating in the countries covered by this study, grouped in regional clusters. Additional information on the national systems can be found in Table Ai in the online appendix. In Nordic countries and some countries in Continental Europe, the majority of studies are of interventions aimed at the general unemployed population. In countries such as Germany or the Netherlands, unemployment benefits are largely based on compulsory insurance schemes, financed through contributions in addition to income taxes. Voluntary insurance schemes are mainly found among Nordic countries except for Norway. For both compulsory and voluntary schemes, qualifying conditions, including previous employment, contributory requirements, or earnings are attached to unemployment insurance benefits. As they are related to previous earnings, these schemes are also relatively generous. Higher net wage replacement rates can largely be observed among Nordic and Continental European countries, in contrast with English-speaking countries, where traditionally the emphasis is on means-tested provisions. Unemployment insurance benefits represent the first tier of the safety net and, if entitlement ceases due to time limits or sanctions, claimants can access a second tier of the safety net in the form of residualised social assistance, usually financed through taxes. Thus, where European studies report that sanctions lead to labour market exit, it does not necessarily mean exit to no job and no benefit income. By contrast, in countries such as the USA, the UK or Australia, there is only one tier of the safety net, and the value of payments is lower (Esser *et al*., 2013; Immervoll and Knotz, 2018; see also Table 1). Receiving a sanction can mean that claimants have no other source of cash income, although repayable hardship loans may be available in the UK and some non-cash benefits may be available in the US. These differences have implications for the interpretation of evidence from differing national contexts.

Since the pioneering reform of the service delivery system for unemployed people introduced in 1998 in Australia, private organisations have increasingly played a major role in the provision of employment services (e.g. job search, placement and training) across North-American and European countries (van Berkel *et al*., 2011). By introducing market competition in service provision processes, this shift has given rise to so-called ‘quasi-markets’, where publicly funded employment schemes are increasingly sub-contracted to private service providers. While aimed at improving the cost-effectiveness of service provision, the partial privatisation of tax-financed services was also accompanied by ‘black-box’ contracting, giving providers a higher degree of discretion to focus on ‘what works’ in terms of service design and delivery (Finn, 2016). In some countries the shift towards a performance-driven approach has created a range of distortions. This is the case for the UK, where the emphasis on short-term targets in the management of public employment services has led to higher benefit off-flow rates. These appear to be achieved through a preferential treatment of more readily employable claimants and the inappropriate imposition of harsher sanctions to encourage claim drops (House of Commons Work and Pensions Committee, 2014). Other countries have adopted a more beneficiary-focused approach, such as the ‘voucher system’ in Germany, where claimants may use vouchers to purchase placement or training services from public or private providers, and the Netherlands where unemployment benefit recipients may select a provider for their labour market integration plan (van Berkel *et al*., 2011; Powers, 2017). This has clear implications for the longer-term outcomes, in terms of both job quality and stability, which need to be taken into account when interpreting the findings reported by the studies in this scoping review.

## 3 The mechanisms of sanction impacts

Griggs and Evans (2010) distinguish between take-up, threat, warning and imposition effects of sanctions. Take-up effects occur by discouraging eligible individuals from applying for benefits in the first place. Threat effects refer to the general pressure on claimants to comply with requirements, whilst warning effects result from formal sanction warnings, where such provisions exist. Imposition effects occur when an applied sanction results in a loss of benefit income. The majority of studies considered in this review capture imposition effects, reflecting the limited use of warnings internationally and the fact that it is more straightforward to estimate the impact of sanctions that have actually been applied. Studies that measure existing or changing rules, however, arguably capture a combined form of sanction effect. Attempts have also been made to estimate threat effects separately from the influence of the job-search requirements that they underpin.

The economic literature utilises job-search theory to understand labour market impacts of sanction policies (Abbring *et al*., 2005). Job-search theory implies that both the threat and the imposition of sanctions will increase exits to employment, by reducing the relative value of continuing to claim unemployment benefits. Sanctions increase the monetary and non-monetary costs of being unemployed, leading individuals to increase job-search efforts and to lower wage expectations, thereby increasing their likelihood of finding employment. Formal warnings exert a similar effect by signalling that a sanction is likely to be enforced (Lalive *et al*., 2005). Importantly, however, actual effects are contingent on benefit design. Threat effects, for example, will be ineffective if they simply lead to a direct substitution of formal for informal job-search methods (van den Berg and van der Klaauw, 2006).

Job-search theory provides inconclusive predictions with regard to post-unemployment outcomes such as job quality (Arni *et al*., 2013; van den Berg and Vikström, 2014). Shorter unemployment durations may help individuals secure work at their pre-unemployment occupational level, which might be expected to have beneficial implications for initial wages, future earnings and job stability. However, sanctions may encourage individuals to lower their wage expectations to find work, therefore increasing the likelihood that they will accept lower quality jobs than they would otherwise secure. Arni *et al*. (2013) also argue that sanctions policy could increase transitions out of the labour force itself, though it is unclear how prevalent this effect is expected to be or how long it might last.

Job-search theory implies that more severe sanctions will have larger threat and imposition effects (Hofmann, 2012). The availability of substitute income sources, such as access to alternative benefits, hardship payments or informal assistance from friends and family, will therefore also be influential. Critics emphasise that unemployed individuals require adequate financial resources to conduct effective job search, and therefore contest the expected link between sanctions and positive employment outcomes (Webster, 2019).

Sanctions may also impact on a wide range of areas including health, debt and financial problems, homelessness or crime (Griggs and Evans, 2010; Watts *et al*., 2014). In part, these arise through the immediate financial impacts of sanctions. These can be expected to initiate or worsen pre-existing debts, rent and utility arrears and severely restrict expenditure on basic necessities, such as food, heating and electricity (Dwyer, 2018). However, non-financial routes are also argued to be important. For health, for example, psychosocial aspects have been highlighted as sanctions may heighten stress and anxiety from negative social attitudes and stigma, not just material hardship. One recent review which considered the impacts of reductions in social security across high-income countries found negative effects for mental health outcomes (Simpson *et al*., 2021). Such effects may persist in the longer term, due to the potential adverse impact of sanctions on job quality and labour force attachment.

Impacts on sanctioned adults may also affect children in the household (Griggs and Evans, 2010). Both material and psychosocial pathways are again relevant. For example, sanctions can increase parental stress which may affect parent-child relationships and child development, while lack of funds for school-related costs such as food and transport can lead to reduced school attendance (Peters and Joyce, 2006; Dwyer, 2018). If sanctions are associated with longer-term adverse labour market consequences for adults, wider research indicates that children are likely to be adversely affected as well. A recent systematic review finds that household income has a causal influence on children’s outcomes, including their health, cognitive, social and behavioural development (Cooper and Stewart, 2020). Detrimental impacts are driven directly by restricted financial resources, which affects housing and diet, but also by the associated financial stress and its impact on parenting behaviours, potential abuse and neglect.

## 4 Study design typology

There is a widely-recognised classification of research designs into a hierarchy with three broad types, based on their value for the identification of causal relationships: non-experimental, quasi-experimental and experimental designs (Murnane and Willett, 2010; Angrist and Pischke, 2009). At times in the analysis, we further divide the first group into three sub-categories reflecting their relative ability to identify causal relationships (Table 2).

In the non-experimental group, Type 1a comprises descriptive studies based on bivariate analysis and studies based on simple multivariable regression techniques, such as linear regression and logistic/probit models. In these models, outcomes are generally compared across exposed and unexposed individuals while covariate adjustment is used to account for potential confounders that may be associated with both the exposure and the outcome of interest. Conventional regression analyses lie at the lowest end of the continuum as they only control for observed variations. They can only make weak claims that observed relationships indicate causal effects since these may be biased by unmeasured confounders. Type 1b includes more advanced regression-based approaches which, depending on how they are implemented, can control for some unmeasured confounding and hence provide estimates which are likely to be closer to causal effects. These include survival models, and fixed- and random-effects models. Type 1c covers designs based on matching techniques relying on covariate adjustment to estimate a propensity score which is the probability of an individual being assigned to or receiving an intervention. Estimates may still be affected by residual and unmeasured confounding, as with other regression techniques.

We note that, in Type 1b, we include studies based on a timing-of-events approach (Abbring and van den Berg, 2003) using mixed proportional hazards models. These can be considered a form of competing risks models allowing for potential unobservable confounding, so could plausibly be included with the quasi-experimental designs. For now, we group them here due to their commonalities with other approaches in the group.

Type 2 covers quasi-experimental approaches and is quite heterogeneous. Difference-in-differences models rely on ‘naturally occurring’ policy variations allocating people to treatment ‘as if at random’. They combine comparisons of before and after exposure with comparisons between exposed and unexposed individuals. If interrupted time series include data on an unexposed comparison group, they can be considered a form of difference-in-differences model, where the randomisation mechanism is defined by the calendar time. For both difference-in-differences and interrupted time series, stronger assumptions are needed to increase their credibility, due, for example, to changes over time occurring independently of the exposure and affecting exposed and unexposed groups unequally, or to group composition changing over time.

Type 2 also includes regression discontinuity models which rely on a cut-off or threshold rule on a continuous assignment variable allocating individuals to the treatment or a comparison group. The model compares those just above and below the threshold, looking for corresponding discontinuity in outcomes to estimate the impact of the intervention. Compared to difference-in-differences, regression discontinuity models may offer stronger causal inferences but limited to a restricted region around the threshold (Bärnighausen *et al*., 2017). Instrumental variables models rely on finding an exogenous factor which is related to the intervention but not otherwise related to the outcome of interest and which is also independent of potential confounders.

With all the approaches included in Type 2, the weakness is that the underlying assumptions about ‘as if random’ allocation to treatment or independence of confounders are impossible to prove. Although various kinds of evidence can strengthen claims in this regard, challenges to the interpretation and attribution of causal effects may remain.

At the highest end of the hierarchy lies Type 3 which covers randomised controlled trials. By relying on strict random assignment to allocate individuals to treatment and control groups, researchers can legitimately claim to have eliminated confounding due to unobserved variations so that differences in outcomes have a clear causal interpretation. Even in this case, issues may still remain with the practical application or ensuring compliance with the design (Deaton, 2010). There can be issues with selective attrition after allocation to treatment which need to be clearly accounted for and, with experiments in ‘real world’ settings, there can be issues with ensuring people adhere to the intended treatment and possible spillover effects from treatment to control group.

One additional aspect of the typology of research designs is worth noting – namely, that they do not all seek to estimate the same measure of causal impact or treatment effect. Understanding the differences here helps inform the broad distinction between non-experimental (Type 1) and quasi-experimental (Type 2) designs, as well as the more nuanced distinction among the different approaches within the latter group. For example, among non-experimental (Type 1) designs, linear regression models are considered to offer an estimate of the average treatment effect (ATE) across the population, albeit one which is potentially biased as a result of any unmeasured confounding. Among quasi-experimental (Type 2) designs, difference-in-differences models offer an estimate of the average treatment effect for (or conditional on) the treated (ATT) rather than the whole population. This may provide valid causal inferences to the extent that the composition of the treated and the comparison group is similar and does not change over time (or whether the so-called ‘parallel trends assumption’ is plausible: see Bärnighausen *et al*., 2017). In the context of instrumental variables and regression discontinuity models, the assumption of homogenous treatment effects across all the individuals in a study can be relaxed through the estimation of local average treatment effects (LATE) (Imbens and Angrist, 1994), which identifies the causal effect of the treatment in a group of ‘compliers’ who receive the treatment when their assignment variable shifts from a point just below the threshold to a point just above the threshold. LATE estimates apply only in this restricted area around the thresholds, where the groups of individuals can be deemed balanced with respect to unobserved confounders.

## 5 Methods

### Scoping review

We draw on the seminal framework by Arksey and O’Malley (2005) and more recent advances (Levac *et al*., 2010; Peters *et al*., 2015) to conduct a systematic search and screening of quantitative studies reporting the labour market and the wider impacts of sanctions in high-income countries. We developed a protocol for our scoping review (Pattaro *et al*., 2019) following, where possible, the Preferred Reporting Items for Systematic Review and Meta-Analysis (PRISMA) guidelines (Tricco *et al*., 2018). These ensure that a rigorous, consistent and transparent process is followed. Scoping reviews often aim to map the existing evidence on a particular topic, and may inform subsequent systematic reviews, by providing the baseline knowledge required to establish whether a full systematic review of the evidence is warranted.

### Search strategy

In consultation with an Information Scientist, we iteratively developed an extensive search strategy including many subject headings, keywords, and synonyms for benefit sanctions. Between March and June 2019, we conducted initial electronic searches of eight major social and health sciences bibliographic databases: ASSIA, British Education Index, EconLit, ERIC, PsycINFO, MEDLINE, Scopus, SocINDEX. Results are summarised in Table A2, with full details of the search strategies in Table A3 (both in the online appendix). We also hand searched relevant research and policy organisations’ websites (e.g. Institute of Labor Economics - IZA, National Bureau of Economic Research- NBER, Research Papers in Economics – RePEc, Institute for Evaluation of Labour Market and Education Policy - IFAU, Organisation for Economic Co-operation and Development – OECD, and International Labour Organization - ILO). The combined results of the searches were imported into Endnote and deduplicated.

### Inclusion criteria and study selection process

The studies for this review were selected using the following inclusion criteria:

1. Targeting working-age recipients of unemployment-related and other means-tested benefits in high-income countries;
2. Investigating sanctions applied to these benefits for failure to comply with job-search and other requirements;
3. Quantitative research studies based on either experimental, quasi-experimental or non-experimental designs;
4. Published in the English language;
5. Published between 1990 and 2019.

**Figure 1:**
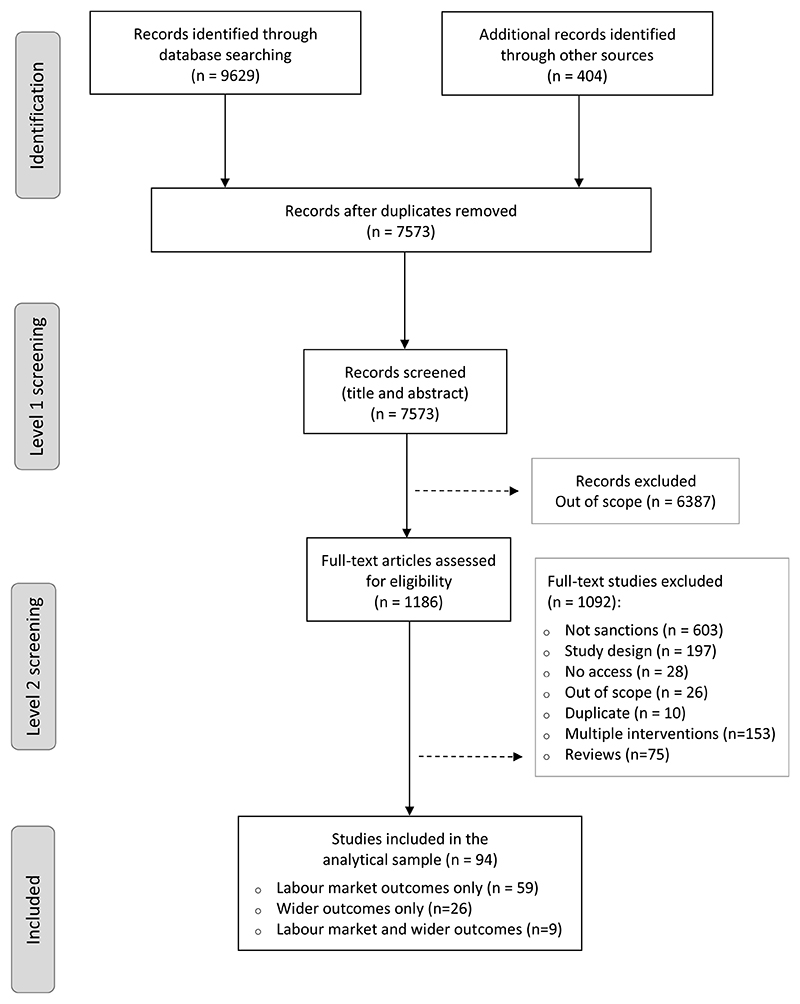
Flow chart representing the study selection process

Four authors (SP, NB, EW and MG) screened and extracted data from the studies included in the review. An overview of the selection process is shown in Figure 1. The electronic database searches yielded 9629 records. These were combined with 404 records identified by the hand searches. Deduplication yielded a total of 7573 records.

Following initial screening to assess whether studies appeared to meet our eligibility criteria based on title and abstract, 6387 (84%) records were excluded due to lack of relevance, publication date, or language. To ensure the reliability of initial screening, 10% of retrieved records were checked by SP and EW. The disagreement rate was 2% (n=19). Discrepancies in the checked group were resolved through discussion with a third researcher (MG).

Initial screening yielded 1186 full-text articles for a second stage of screening, which excluded a further 1092 studies. Of these, a large group (n=603; 55%) were not focused on sanctions, including studies of welfare leavers’ outcomes and the effects of other welfare reforms such as time limit policies or job-search interventions not directly reporting sanction impacts. Working papers already in our database that were subsequently published as a journal article were also excluded. A second group of studies (n=197; 18%) was excluded due to study design, because they were narrative policy analysis papers, commentaries, discussion pieces, general overviews, qualitative studies, theoretical papers and studies based on microsimulation modelling. Another 153 studies (14%) were excluded because they were evaluating multiple simultaneous interventions or policies, precluding identification of the unique impact of sanctions. For example, some used period or policy dummy indicators to identify a set of welfare changes or combined sanctioned individuals with groups affected by other policies. A further 75 studies (7%) were reviews of a number of individual studies using a variety of methodologies from informal narrative review to systematic review. The remaining excluded studies (6%) comprised 28 articles which could not be accessed, 26 out-of-scope studies (published before January 1990, not in English or not from high-income countries) and 10 further duplicates.

Following the second screening, SP and EW conducted an additional review of 118 studies (10% of the sample assessed for eligibility) with a disagreement rate of 21% (n = 25); discrepancies were discussed with MG. The overall discrepancy rate for both screening stages was 5% (n=44).

The screening process identified 94 studies providing original evidence on the impact of benefit sanctions, on which data extraction and analysis were subsequently conducted. The sample comprises 59 studies (63%) reporting only labour market outcomes, 26 studies (28%) reporting wider outcomes only, and 9 studies (9%) reporting both outcomes. Some tables therefore present statistics for 103 studies in total as nine are counted twice. Many studies report results for multiple outcomes; the total number of outcomes is 253.

### Data extraction

A data extraction form was developed to record detailed information for the analytical sample. The form was pilot-tested on a randomly-selected study and subsequently refined on a larger number of studies. We gathered high-level characteristics such as type of outcome reported, population, national context, time period of the intervention and study design. We then extracted more detailed information on the magnitude, sign and statistical significance for the parameters estimated for the outcomes. We also extracted the time horizon of the results (short-, medium- or longer-term) and details on the exposure including the type of sanctions (whether full or partial) and related effect (for instance, whether an imposition or threat of sanctions), along with details on the study design. To ensure consistency of the data extraction phase, SP and EW conducted a review of the data extraction forms compiled for 12 studies (13% of the analytical sample) and discrepancies were discussed (n=1; disagreement rate = 8%), without resorting to a third reviewer.

### Literature analysis and synthesis

**Figure 2:**
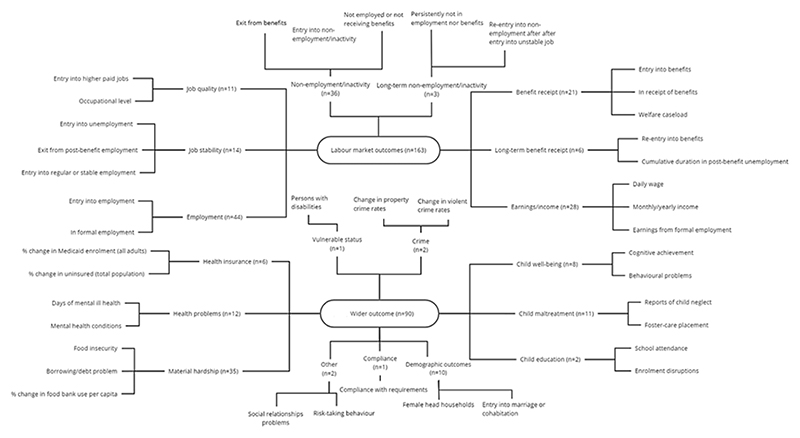
Representation of outcomes and selected measures for studies on labour market and wider outcomes Note: Frequencies are reported at the lowest level of aggregation – namely, for outcome measures (n=253).

We conduct a descriptive analysis of the evidence base by exploring how this varies by main study characteristics. We present a synthesis of effects for eight labour market and eleven wider outcomes, each of which can be assessed by one or more measures as Figure 2 below records.

In synthesising results, we combine two approaches. First, we report simple frequencies and percentages for relevant characteristics across the sample. Where possible, we provide these details at the highest level of aggregation – namely, by broad categories of labour market and wider outcomes (n=103). For the impact of sanctions on the outcomes, we report the sign and significance of estimated parameters at a lower level of aggregation – that is, for outcome measures (n=253; Figure 2 below). We extracted impact data for all reported outcomes, including any subgroups or further subdivisions of impacts reported in the original studies. However, for sake of simplicity, when summarising the data, we report the most prevalent results when these were recorded for multiple subgroups, time horizons or exposure categories. In doing this, we rely on the results foregrounded by the study authors where possible. We provide a narrative summary of the effects, and include comments and examples to clarify any mixed results emerging from the body of evidence.

We report the effect estimate and significance for the outcomes foregrounded by the study authors in Table A4 in the online appendix. However, we do not report effect sizes in the main text, as we were not able to conduct a meta-analysis or calculate common metrics for effect size due to the very large number of heterogeneous outcomes reported by the included studies. This is also standard practice in scoping reviews, which do not typically report standardised effect sizes for included outcomes. For this reason, we do not discuss effect magnitude in the text.

## 6 Scale and nature of the quantitative evidence

In this section we address the first research question by providing an overview of the scale and nature of the quantitative evidence base. Table A4 in the online appendix includes additional details for the studies, ordered alphabetically by author and grouped by outcomes and study design. Details include information on the programme or intervention, outcome measures assessed and key outcome results. The references for the studies are reported in Table A5 in the online appendix. These are ordered using a sequential number which we report jointly with the reference in the text in the remainder of the article.

### Study contexts

**Figure 3:**
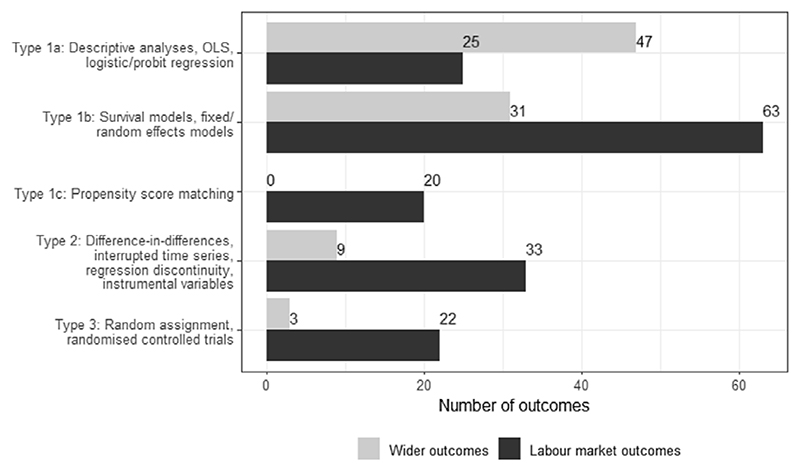
Study design typology by labour market and wider outcome measures Note: Frequencies are reported at the lowest level of aggregation – namely, for outcome measures (n=253).

Table 3 provides an overview of the contexts and nature of the studies included in the scoping review, divided between labour market and wider outcomes (n=103). In general, the labour market literature looks much more substantial. There are twice as many studies of labour market outcomes. They cover more of the potential sanction effects (e.g. threat as well as imposition effects), with a greater use of individual-level data and administrative sources likely to have larger scale. As Figure 3 shows, a larger proportion of these studies can support causal inference.

For both labour market and wider outcomes, the large majority of studies cover the 1990s or 2000s with relatively few for the last decade but this may reflect the lag in the research process in part. By publication period, the labour market literature has the same volume in the last decade as the previous but the wider-outcome literature showed a sharp decline. The United States accounts for the largest share, with 52 percent of labour market and 86 percent of wider outcome studies. For the latter, it is notable that the entire evidence base identified by the scoping review comes from English-speaking countries, with the UK adding four studies and Australia one. This may in itself be one indication of the more severe impact of sanctions in these contexts, as noted in Section 2 above, and hence a greater urgency to produce evidence on these outcomes. By contrast, studies on labour market outcomes include twenty-two from Continental European countries and a further seven from Nordic countries. The Continental European studies are from Germany (n=10), Switzerland (n=5), the Netherlands (n=4), Belgium (n=2) and Hungary (n=1). Among Nordic countries, Denmark has four studies, with Sweden, Norway and Finland one each.

There is an almost-perfect correspondence between target population group and type of programme or intervention covered by the studies. These dimensions also largely overlap with geographical coverage. Studies reporting wider outcomes are primarily US studies of low-income families or lone parents largely in receipt of means-tested benefits in the form of ‘Temporary Assistance for Needy Families’ (TANF) or its antecedent ‘Aid to Families with Dependent Children’ (AFDC). On the other hand, a conspicuous portion of studies reporting labour market outcomes, largely among European countries, focuses on unemployed people in receipt of either contribution-based Unemployment Insurance (UI) or means-tested Unemployment Assistance (UA). Only one study, from the UK’s National Audit Office (2016b [61]) focused on people in receipt of disability benefits.

Looking at the remaining study characteristics, different profiles emerge depending on the outcomes being assessed. Nearly all studies on wider outcomes reported on imposition of sanctions (94%; n=33), either full sanctions (n=11) or not distinguishing full and partial sanctions (n=22). By contrast, among studies of labour market outcomes, a large share reported either an imposition or a threat of sanctions (85%, n=58), and there was a split between those examining full sanctions (n=29) and those looking at partial sanctions (n=16). For TANF benefits in the US, sanctions may extend beyond the portion of benefits attributable to the non-compliant household member to the benefits for the entire household, including children.

Three quarters of studies of labour market outcomes used sanction indicators measured at an individual level (75%, n=51). Studies of wider outcomes were more evenly divided, relying on sanction indicators measured at both individual (51%, n=18) and area levels (49%, n=17). The latter included indicators measured at state, regional or local area level. Of these, approximately one third (n=6 and n=7, respectively) also employed area-level units of analysis. These are largely based on non-experimental designs and may suffer from additional problems related to ecological fallacies, whereby individual-level inferences are incorrectly derived from correlations observed at the area-level, as recognised by some authors (e.g. Loopstra *et al*., 2015 [83]).

While a significant proportion of the labour market studies use administrative data (57%; n=39), among studies on wider outcomes, data sources are more diverse, with survey data the most common source (43%; n=15), followed by linked administrative-survey data and administrative data (29%, n=10, and 26%, n=9, respectively). This may be linked to the fact that a larger proportion of non-labour market studies are based on non-experimental study designs in contrast with quasi- or experimental designs used more commonly among labour market studies.

### Outcomes

Figure 2 summarises the specific outcomes examined along with selected measures used in each case. It shows the enormous diversity across the literature. In our review, we identified 253 outcomes measures across the 94 studies. Of these, 163 (64%) related to the labour market. From these, we identified eight specific outcomes (Figure 2, top panel): employment; job stability; job quality; non-employment/economic inactivity in short- and long-term; benefits receipt in short- and long-term; and earnings/income. Within each of these outcomes, a range of measures might be used. With employment, for example, we identified 44 measures, mostly referring to either employment status or entry into employment. Job stability and quality were assessed through 25 measures. These appeared in studies examining, for example, whether unemployed individuals entered regular employment in the longer-term or transitioned to jobs which were better paid (e.g.: Hofmann, 2012 [16]; van den Berg and Vikström, 2014 [44]). Entry into non-employment or economic inactivity also included exits to an unknown destination. Nearly a quarter of labour market-related measures fall into the combined category of non-employment or economic inactivity, and long-term non-employment/inactivity (n=39). The remaining measures are equally divided between those relating to benefits and those relating to either earnings from employment or income. In our sample, we have a total of 27 measures (17%) regarding either re-entry into benefits or long-term persistence of benefit receipt and 28 measures (17%) regarding earnings from employment and/or other sources of income.

Among studies concerned with wider outcomes, we identify five broad groups: material hardship; health-related outcomes, covering health problems and access to health insurance; child outcomes, including well-being, maltreatment and education; demographic outcomes; and a last group including vulnerable status, crime, compliance and other outcomes (Figure 2, bottom panel). Material hardship was covered by 35 measures (39%) assessed through measures including food insecurity, financial hardship, housing problems, or utility cutoffs, as well as impacts of material hardship on adult health or on children’s opportunities. In two studies, it was not possible to disaggregate the last two measures and we reported these as part of material hardship (Lindhorst and Mancoske, 2006 [26]; Lindhorst *et al*., 2000 [27]). Health problems and health insurance status were assessed through 18 measures (20%) for adults but also children. Additionally, we identified 21 measures (23%) for child outcomes including well-being, maltreatment and educational outcomes. Ten measures (11%) concerned demographic outcomes, including entry into marriage, cohabitation or female household headship. We identified one measure for vulnerable status and two measures for crime. Compliance was quantified through one measure where other outcomes pertained to social relationship problems (n=1) and risk-taking behaviour (n=1).

While all labour market measures concerned working-age adults (of necessity), only half of the wider outcome studies focussed on adults (n=42), with one third looking at children (n=29) and a further fifth covering both adults and children (n=19). In terms of time horizons for outcomes, more than half of the wider outcome studies (n=46) looked at the short-term (i.e. within the first year following a sanction), whereas for labour market studies this was less than 40 percent (results not shown).

### Study design

The distribution of the study designs is presented in Figure 3. The majority of studies across both labour market and wider outcomes rely on non-experimental designs (Types 1a-1c), with these making up a much larger proportion ofthe latter group. For labour market studies, more than a third of the outcome measures are from quasi-experimental or experimental designs (34%; 55 out of 163), but for wider outcome studies, the figure is even lower (13%; 12 out of 90). Among the latter, the literature is dominated by Types 1a (n=47) or 1b (n=31). This highlights the fact that causal relationships cannot be established by many studies in this policy area.

## 7 The impact of sanctions

**Figure 4:**
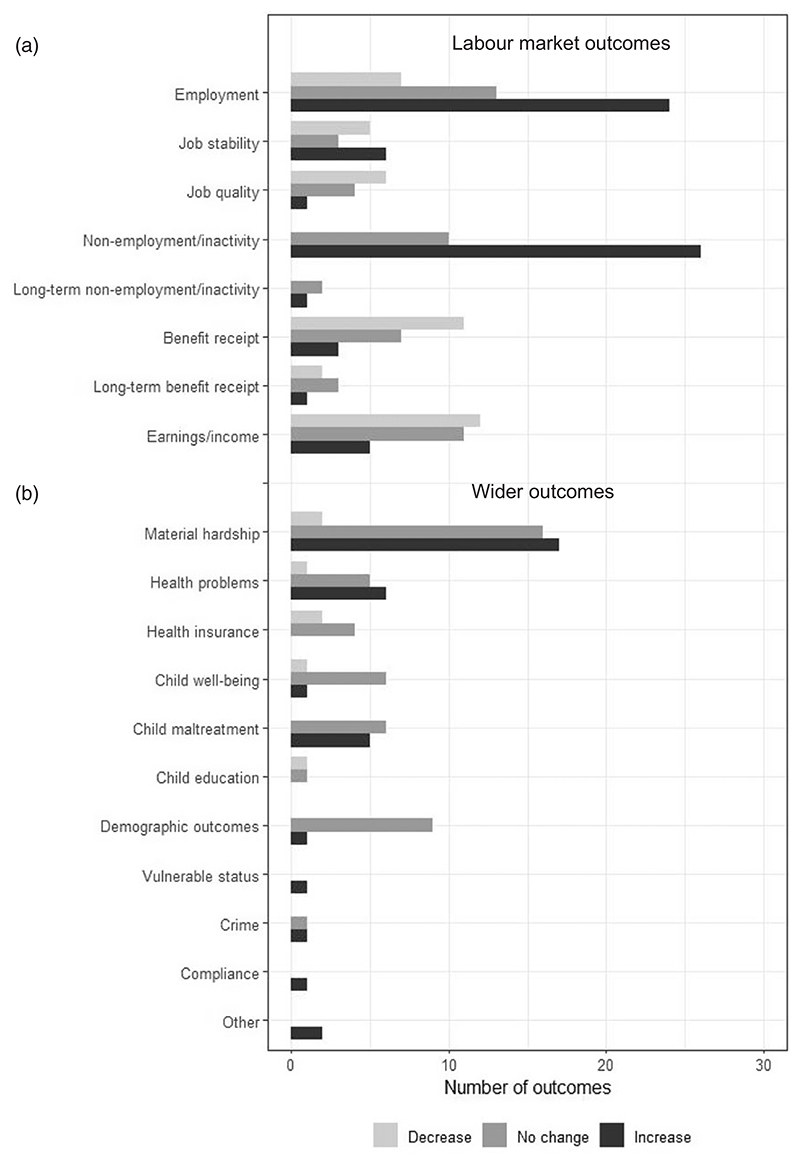
Direction of results by type of labour market and wider outcome Note: Frequencies are reported at the lowest level of aggregation – namely, for outcome measures (n=253).

In this section we address the second research question by presenting a synthesis of the results of the impact of sanctions across labour market and wider outcomes. We also look at variations by study design. Figure 4 shows the number of times measures displayed a significant increase, significant decrease or no change for each outcome identified. Table 4 disaggregates these by the three main types of study design while the text refers to the finer categories of study design where relevant.

### Labour market outcomes

With the labour market literature, a large proportion reported a positive impact of sanctions on employment outcomes (Figure 4, panel a). However, sanctions appear to be associated with adverse or null impacts on job quality in the longer term. Sanctions also seem to be associated with both a significant increase in exits from benefits to non-employment or economic inactivity, and a significant decrease in benefit receipt. A large share of studies reported negative or null impacts on earnings or income.

#### Employment status and entry

A total of 44 outcomes relating to employment status or entry into employment were reported. Of these, just over half (n=24; 55%) reported a positive association with the threat or imposition of sanctions, while 13 (30%) reported no impact, and 7 (16%) reported negative effects (Figure 4 and Table 4). The majority (n=30; 68%) were from non-experimental studies that were less likely to show an increase in employment (14 out of 30). However, the quasi-experimental and experimental studies were much more likely to do so (10 out of 14).

For positive employment outcomes reported by non-experimental studies (n=14), the majority relied on the timing-of-events approach (n=11), covering mostly Continental European countries. As noted in Section 4 above, this is an approach which could plausibly be included with the quasi-experimental category. Two studies from Germany (Müller and Steiner, 2008 [31]; Hohenleitner and Hillmann, 2019a [17]) and one from the US (Peck, 2007 [34]) used propensity score matching. Various methods were used by the quasi-experimental group, covering two studies from Belgium and Switzerland (Cockx and Dejemeppe, 2007 [56]; Arni and Schiprowski, 2016 [53], respectively) and one from the UK (National Audit Office, 2016b [61]). By contrast, the experimental studies relied on random assignment of study participants to intervention and control group. These were largely from the US, except from one study from Hungary (Micklewright and Nagy, 2010 [65]). Within the non-experimental group, numerous studies mostly coming from the US also reported either a significant decrease or no change in employment outcomes following a sanction (n=16). While most of the studies reporting no change in employment outcomes relied on survival modelling or descriptive analysis (9 studies reporting a total of 11 outcomes), those reporting a significant decrease relied on conventional regression models (n=5). There are four studies among the quasi-experimental group which reported either no change or a significant decrease in employment outcomes, largely in the short-term. While the former are Continental European studies (Cockx and Dejemeppe, 2012 [57]; Arni and Schiprowski, 2016 [53]), the latter are from the UK (National Audit Office, 2016b [61]; Taulbut *et al*., 2018 [62]).

#### Job stability and quality

Fewer outcomes relating to job stability and quality were reported, mostly from non-experimental studies (Table 4, panel a, and Figure 4). Of 14 job stability outcomes, there were positive effects for 6, no effect on 3, and 5 found that job stability decreased. Eleven job quality outcomes included one positive impact, 6 negative associations, and no effect in 4 cases. Between the two outcomes, the majority of effects were either negative or null (n=11 and n=7), suggesting that sanctions may not promote job stability or quality. Evidence for these measures was dominated by outcomes from non-experimental studies (n=17; 68%), with no experimental studies reporting measures of job stability or quality. The direction of effects was quite inconsistent with too few to make useful comparisons on the basis of study design.

Negative or null job stability effects were reported mainly by Continental European studies with outcomes measured mostly in the medium-term, whereas similar results for job quality were reported mostly in the longer-term by three US studies and two Continental European studies. For both outcomes, most studies relied on survival modelling techniques (n=7), whereas two German studies applied matching techniques (Hofmann, 2012 [16]; Hohenleitner and Hillmann, 2019b [18]). Although largely in the short-term, negative or no impacts were also exhibited by quasi-experimental studies for European countries, mostly relying on instrumental variables (National Audit Office, 2016b [61]; Arni and Schiprowski, 2019 [54]).

#### Entry into non-employment or economic inactivity

Adverse labour market impacts were also reported in terms of a significant increase in transitions to non-employment or economic inactivity. These were mostly measured through exits from benefits or transitions to destinations other than benefit receipt or gainful employment. An increase in the risks of exit to non-employment or inactivity was recorded for 26 (72%) out of a total of 36 outcomes. For the remaining 10 outcomes (28%), no effect was reported. Only three long-term non-employment or inactivity outcomes were reported, with inconsistent findings.

Most studies reporting a positive association with non-employment/inactivity outcomes were from Continental European countries and relied on survival modelling or a timing-of-events approach (n=14; 70%). Note that, in many of these countries, sanctioned unemployment insurance claimants would have access to second-tier social assistance benefits. The only exception was a UK study (Reeves, 2017 [37]) which used fixed effects models applied to area-level data to investigate the impact of a recent reform of the UK Job Seekers’ Allowance regime. Four outcomes were reported from three European studies using matching techniques (Lissenburgh (2004 [28]) for the UK; Hofmann (2012 [16]) and Hohenleitner and Hillmann (2019a [17]) for Germany). Using quasi-experimental designs, positive impacts of sanctions on nonemployment or economic inactivity were found for five measures, as reported by three European studies using instrumental variables (Boockmann *et al*. (2014 [55]) for Germany; National Audit Office (2016b [61]) for the UK; Arni and Schiprowski (2019 [54]) for Switzerland). One descriptive study (Ovwigho *et al*., 2011 [33]) and one experimental study (Olson *et al*., 1997 [66]) reported no change within the US context, while a quasi-experimental study (Arni and Schiprowski, 2016 [53]) reported an increase in long-term inactivity within a Swiss context.

#### Benefit receipt

Overall, there were twenty-one outcomes measured for benefit receipt which referred to either re-entry to benefits, the amount of benefit received, or the number of people claiming benefits. Among these, more than half (53%; n=11) reported a significant decrease following an imposition of a sanction, while no statistically significant effects were reported by one third (n=7) and a significant increase was reported by just three studies (14%). Six long-term benefit receipt outcomes were reported by non-experimental studies. Of these, there was no effect in three cases, a reduction in two, and an increase in one.

Studies from two Nordic countries, applying survival modelling (Diop-Christensen (2015 [10]) for Denmark) and a timing-of-events approach (Busk (2016 [7]) for Finland) generated a positive association and no effect, respectively. Two positive and two negative impacts were reported for outcomes from quasi-experimental studies (n=4), while there was no effect on three outcomes from US studies based on random assignment exercises and a negative effect in a further four. Within this group, one study (Scrivener and Walter, 2001 [68]) reported two negative and one null effect.

#### Earnings and income

We identified 28 outcomes reporting effects on earnings and/or income. A large share of these reported either a significant reduction (n=12; 43%) or no effects (n=11; 39%) while a significant increase was reported by five studies (18%). Non-experimental designs predominantly showed a reduction or no change in income (n=9 and n=7 out of 17). The negative results employed descriptive analyses (n=3), probit regression (n=1), survival or timing-of-events models (n=3) and propensity score matching (n=2). The quasi-experimental or experimental studies had slightly less negative results (n=3 out of 11). Notably, although only six earnings-related outcomes were reported by experimental studies from the US context, four of these found a positive association with sanctions, while two found no effect. Although a large majority of effects were negative (n=12) or null (n=11), indicating that sanctions are associated with a reduction or no change in earnings or income, most of the findings from experimental studies suggested a positive impact.

### Wider outcomes

Fewer studies reported results on wider outcomes measures (n=90) (Table 4 and Figure 4, panel b). Almost all use non-experimental methods (n=78) and all stem from English-speaking countries, such as the US, UK and Australia. A large proportion of studies reported an increase in both material hardship, such as financial distress and food insecurity, and adverse health outcomes for adults and children. A significant association was also shown with adverse child outcomes, such as child maltreatment, poorer child well-being and educational outcomes. While the results on child maltreatment were corroborated by one quasi-experimental study, for child well-being there were some inconsistencies across study designs. A significant increase in survival crime was also reported by one quasi-experimental study.

#### Material hardship

Material hardship was assessed though measures such as food insecurity, inability to pay rent or utility bills, borrowing and debt problems. Positive associations with material hardship were observed for 17 outcomes (48%), while 16 outcomes reported no significant associations (46%); for only two outcomes there was a negative association or improvement in welfare (6%). All the evidence on material hardship hinges on non-experimental designs, mostly relying on descriptive and standard regression techniques. Two exceptions employed fixed effects models: a study by Reichman *et al*. (2005 [87]) for the US which found a positive relationship with food poverty and utility cut-offs, and a study by Loopstra *et al*. (2018 [82]) for the UK which found a positive relationship with food bank use.

All 35 studies reporting outcomes on material hardship were from English-speaking countries, with US covering the vast majority, UK contributing two studies and Australia one. A significant increase in food insecurity and poverty was reported by 8 studies out of 15 (53%), with just one US study based on state-level aggregated data reporting a significant reduction in poverty rates (Rodgers *et al*., 2006 [88]). A significant increase in difficulties in paying bills and the experience of utility cut-offs was reported by 6 studies out of 9 (67%). In addition, 3 out of 5 studies (60%) displayed a significant increase in issues related to health insurance coverage for the adult or parent, while no associations were reported for children by one study (Lindhorst and Mancoske, 2006 [26]). Adverse schooling outcomes were reported for children in a study by Oggins and Fleming (2001 [32]). For problems concerning both borrowing and debt, and housing-related problems, including homelessness and eviction, the majority of the studies reported no significant associations with the imposition of sanctions (75%, 3 out of 4 studies, and 67%, 4 out of 6 studies, respectively).

#### Health problems and health insurance status

Health problems were largely quantified using self-reported measures relating to mental and physical health, which referred to either adults/parents or children. In the case of children, these were reported by one of the parents. Other measures included indicators related to hospitalisation and doctor consultations (Cook *et al*., 2002 [72]; Baltagi and Yen, 2016 [69]).

A significant increase in health problems was shown for 6 out of 12 measures. Of the remainder, five reported no statistically significant associations, while one reported a significant negative association. While a significant reduction in health insurance coverage was reported for two out of six outcomes (33%), null associations were reported for four. The first two came from the study by Moffitt (2000 [29]), while the latter four were from Chavkin *et al*. (2000 [8]). These results are in line with those on health insurance coverage recorded as part of material hardship (see section above). Across the board, most studies were from the US, except for one Australian study by Eardley (2006 [75]). The majority applied either standard regression or fixed/random effects models.

### Child outcomes: well-being, maltreatment and education

There are mixed results for the effects of sanctions on child well-being. We identified three US studies reporting eight measures relating to child well-being (Chase-Lansdale *et al*., 2002 [71]; Lohman *et al*., 2004 [81]; Wang, 2015 [93]). These generally included measures regarding cognitive achievement and behavioural problems. Most measures (n=6; 75%) showed no sanction effects for child well-being, while a significant increase was found for behavioural problems (n=1) and a significant reduction for cognitive achievement (n=1). These two results both appeared in the study by Lohman *et al*. (2004 [81]), which was based on descriptive analyses, similarly to Chase-Lansdale *et al*. (2002 [71]). Adopting a quasi-experimental design, the study by Wang (2015 [93]) combined propensity score matching with a difference-in-differences modelling approach. The author used a composite outcome measure by combining multiple items, including cognitive development, family’s interactions and stress, and educational outcomes. For each of these measures a nil impact was shown for the imposition of sanctions.

We identified a total of 11 measures on child maltreatment, including (indicated or substantiated) reports of abuse or neglect as well as foster-care placement. For the majority of the measures, no statistically significant effects were found (n=6; 55%), while a significant increase (worsening) was reported for the remaining measures. In the latter case, when studies provided a significant increase, this was largely reported for substantiated cases of child neglect or mal-treatment (Fein and Lee, 2003 [94]; Ovwigho *et al*., 2003 [84]; Paxson and Waldfogel, 2003 [85]; Slack *et al*., 2007 [90]). For foster-care placement, results were less clear with Paxson and Waldfogel’s non-experimental study (2003 [85]) reporting a statistically positive effect whilst Fein and Lee’s experimental study (2003 [94]) recorded a null effect. All the non-experimental studies were based on survival modelling applications.

The evidence base on children’s educational outcomes is rather scant, resting on a single non-experimental study by Larson *et al*. (2011 [79]) using descriptive analyses. The study reported a negative association between the imposition of sanctions and school attendance rate, while no effect was reported for enrolment disruptions. Mixed results were also reported for children’s educational outcomes, as part of material hardship as noted above.

### Demographic outcomes

Demographic outcomes were quantified by measures such as entry into marriage or cohabitation, non-marital childbearing or female household headship, and living arrangements of both adults and children. We identified a total of ten demographic outcomes, mostly reporting no statistically significant associations with the imposition of sanctions. Consistent findings were reported across non- and quasi-experimental studies, six of which applied survival models including fixed effects, while one used a difference-in-differences model. The only exception within the non-experimental group was a UK study by Reeves and Loopstra (2017 [86]) applying a fixed effects model on area-level data and reporting a significant positive association with areas with a higher proportion of lone parents.

### Vulnerable status, crime, compliance and other outcomes

We identified one study from the UK which applied fixed-effects models and found a significant positive relationship between sanctions and vulnerable status, measured through the proportion of unemployment benefit claimants with a disability (Reeves and Loopstra, 2017 [86]). Another UK study, relying on a difference-in-differences model, reported a significant positive impact of sanctions on survival crime rates but a nil impact on violent crime rates (Machin and Marie, 2006 [92]). A significant increase in compliance with requirements and other outcomes – namely, social relationship problems and risk-taking behaviour, was reported by a non-experimental study for Australia (Eardley, 2006 [75]).

## 8 Conclusion

### Summary of the results

Our scoping review describes the evidence base relating to the impact of benefit sanctions. The review makes an original contribution through its application of comprehensive searching, screening and data extraction processes to the international quantitative research evidence on labour market and wider outcomes. We are not aware of any previous attempt to systematically identify and synthesise the latter literature. The review relies on a rigorous methodology to provide transparency and reduce the potential for reviewer selection to bias findings. We do not attempt a systematic review or meta-analysis of results at this stage but look at where the preponderance of the evidence lies. However, in our narrative summary, we do examine the study designs employed using an extended hierarchy to assess the robustness of the evidence base.

Our scoping review identified 94 studies providing novel quantitative evidence on the labour market and/or wider impacts of sanctions which met our inclusion criteria. From these, we identified 253 outcome measures, of which nearly two thirds related to labour market outcomes while one third covered wider outcomes. The literature on labour market outcomes was not only larger but also had a higher proportion of studies employing research designs which are better suited to supporting causal claims. In general, however, studies employing quasi-experimental or experimental methods did not diverge substantially in their findings from those employing non-experimental methods.

Labour market studies produced evidence of a positive impact of sanctions on employment outcomes. This is consistent with the findings from existing reviews (e.g. Griggs and Evans, 2010; McVicar, 2020). However, our review also highlighted that sanctions were associated with a range of adverse impacts in terms of worsening job quality and stability in the longer term, along with higher rates of exits to non-employment or economic inactivity, and more rapid returns to benefit claiming. Null or negative impacts were shown for earnings or income measures.

The evidence base on wider outcomes was not only considerably smaller but also dominated by non-experimental studies. The studies reported a wide range of negative impacts. The imposition of sanctions was associated with an increase in material hardship, including food deprivation and the experience of financial hardships. Sanctions were also associated with worse physical and mental health and decreased access to healthcare insurance. For outcomes related to children, there was some evidence that sanctions were associated with an increase in child maltreatment as well as behavioural problems and poorer cognitive development. There were no significant associations between sanctions and demographic outcomes, such as non-marital childbearing or living arrangements for adults and children. There was some evidence of increases in crime and worsening child education for a small number of studies.

### Results in context

All studies reporting wider outcomes originated from English-speaking countries, with the US covering a large proportion, while labour market studies included a significant share from Continental and Nordic European countries. This geographical divide also reflects heterogenous policy intervention programmes and different degrees of severity of the sanctioning regimes across the regions, with US welfare-to-work programmes targeting low-income families and lone parents, while sanctioning among the included European studies is directed towards unemployed claimants in receipt of either unemployment insurance or unemployment assistance benefits. While the sanctioning regime of the former, along with that of the UK, features a safety net with just a single tier, several European countries present an additional tier of the safety net in the form of means-tested social assistance (e.g. Esser *et al*., 2013); this may have implications for the nature of the impacts that we observe. Further investigation of the role played by the context in which sanction regimes operate would require conducting a full meta-analysis at least for studies reporting labour market outcomes.

### Literature gaps

In contrast to an increasing number of studies focussing on labour market outcomes, we observed a reduction in the evidence base on wider outcomes over time. While the former is increasingly based on quasi-experimental or experimental designs, the latter uses predominantly non-experimental designs. This highlights an important gap in the literature on wider outcomes, as the evidence base may be affected by issues concerning unobserved confounding that limit the causal inferences that can be drawn.

Concerning the wider impacts of benefit sanctions, our review revealed some areas which remain under-investigated. One area concerns the housing impacts of benefit sanctions, in terms of rent arrears, eviction and homelessness. A recent UK study by Hardie (2020), examining the effects of the recently implemented Universal Credit programme (which merges pre-existing means-tested working-age benefits), found that benefit conditionality and sanctioning were associated with increased landlord repossession rates. An additional area where we found either inconsistent or limited evidence concerns child well-being, including educational and health outcomes. A recent review on the effects of social security reforms on mental health in high-income countries has reached similar conclusions concerning child health outcomes and acknowledged that these have important implications for health, education and employment opportunities of children as they progress through different stages of their life course (Simpson *et al*., 2021).

### Limitations and future research directions

There are two main limitations that should be considered when interpreting the findings of this review. One limitation concerns the scope of the literature which is captured by the review. As is common with other scoping reviews, this is highly dependent on the inclusion criteria established at the outset. While the search strategy was designed to identify as many relevant studies as possible, we cannot be certain that all such studies were found. For example, by excluding studies not published in the English language or including only studies available at the time when the searches were completed, it is possible that we did not capture some relevant studies. In addition, in the econometrics literature, many studies are working papers which do not use consistent key words and are accessed via websites with basic search functions. Further, indexing in economics journals is not always optimal. We developed a comprehensive search strategy and hand searched many relevant repositories of working papers in an effort to overcome this limitation.

A second limitation relates to the synthesis of the results from the studies reviewed. Although we extracted impact data for all reported outcomes, including any subgroups, time horizons of the results and exposure categories, in the context of a scoping review, it was not possible to encompass this level of detail. We used a ‘vote counting approach’ based on direction and significance of effect to provide a summary of the impacts of sanctions. Vote counting has well recognised limitations, including lack of weighting for sample size and not accounting for effect magnitude or precision of estimates. However, in the context of a scoping review with a large number of heterogenous outcomes we feel that vote counting provides an accessible high-level summary of trends in the data.

For future, there is clearly potential to extend the exercise undertaken for this scoping review to a full systematic review, including a critical appraisal of the evidence base, a detailed narrative synthesis and, if possible, a meta-analysis of the impacts. The latter would be dependent on having a degree of homogeneity in the outcome measures and related estimated parameters and this may be particularly difficult for the studies reporting wider outcomes. However, if possible, it would provide the basis for producing more holistic estimates of the societal costs and benefits from sanctions which could offer a valuable input to policy making.

This review also highlights the urgent need for more studies of the impacts of sanctions to extend the knowledge base in this contested policy field. In particular, there is an urgent need for more studies to examine the wider outcomes of sanctions using quasi-experimental or experimental methods. A strong commitment from policy makers to improve the evidence base would be invaluable here since the design of policies (e.g. the use of controlled trials or phased rollouts) can greatly aid the delivery of strong evidence.

### Policy implications

The evidence reviewed casts serious doubts on the sanctions policies being pursued in many countries, particularly those which have expanded the reach and increased the intensity of sanctions regimes in recent years. The evidence does not seem to show that sanctions ‘work’. Rather it shows that, while there may be some positive outcomes in relation to often stated goals for sanctions, these are accompanied by a range of null and negative outcomes. In relation to the labour market, while sanctions tend to increase exits to employment in the short term, there is evidence of adverse impacts on job quality, job stability, earnings and income, and of increased exits to non-employment or inactivity. Taking a wider perspective, and acknowledging that the evidence here is thinner and weaker, the high proportion of adverse impacts on measures of material hardship, health, and child outcomes is sufficient to give significant cause for concern. In this area in particular, the findings from the scoping review corroborate and are reinforced by the evidence from numerous qualitative studies. Given the potential for a range of significant and long-lasting harms for welfare benefit claimants and their children, and in the absence of clearer evidence of other benefits, policy-makers should give serious consideration to limiting policies which deprive people of income.

## Supplementary Material

Supplementary material 1

Supplementary material 2

## Figures and Tables

**Table 1:** Characteristics of social/unemployment protection and sanction policies by selected regions and countries, 2018-2019

Region/country	Benefit type	Qualifying period/earnings	Benefit amount	Initial net replacement rate^a^	Maximum benefit duration (months)	Reduction in benefit for most severe sanction	Duration of sanction (months)
*Nordic countries:*							
Denmark	Unemployment insurance (UI)	DKK 233,376 last 3 years, plus min. 12 months of employment and payment membership fee	Max. DKK 18,866/month	72	24 in 3 years	Termination of payment	n/a
Finland	Unemployment insurance (UI)	26 weeks, last 28 months (min. 18 hours/week) Earnings-related benefit: min. 26 weeks of membership unemployment fund	EUR 32.40/day (flat-rate) Max. EUR 143.16/day^b^ (earnings-related benefit)	75	20	Suspension of payment	2
	Unemployment assistance (UA)	n/a	Same as flat-rate UI	n/a	Unlimited	n/a	n/a
Norway	Unemployment insurance (UI)	Min. earnings: 1.5 times or 3 times the annual base amount^c^, last 1 or 3 years respectively	Daily benefit: 0.24% of annual base amount^c^	73	26	Suspension of payment	6
Sweden	Unemployment insurance (UI)	6 months, last 12 months; membership of insurance fund for min. 12 months	Max income-related benefit: SEK 910/day, first 100 days; after: SEK 760/day; Basic insurance: SEK 365/day	65	10	Termination of payment	6.5
*Continental European countries:*							
Belgium	Unemployment insurance (UI)	468 days, last 27 months	n/a	71	Unlimited	Termination of payment	1-12
Netherlands	Unemployment insurance (UI)	26 weeks, last 36 weeks: week's condition, short-term benefits 208 hours, last 4 out of 5 years: year’s condition, medium-term benefits	Max. EUR 216.90/month, first 2 months	74	3: short-term benefit (week’s condition) 24: medium-term benefit (year’s condition)	Suspension of of payment	n/a
Germany	Unemployment insurance (UI)	Min. 12 months, last 2 years	n/a	72	6-24, depend on age, contribution period	Suspension of payment	3
	Unemployment Assistance (UA)	n/a	EUR 416/month (flat-rate)	n/a	Unlimited, revised after 12 months	n/a	n/a
Switzerland	Unemployment insurance (UI)	Min. 12 months, last 2 years	n/a	85	18	Suspension of payment	2
Hungary	Unemployment insurance (UI)	360 days, last 3 years	Max. HUF 149,000/month	54	3	Termination of payment	n/a
*English-speaking countries:*							
UK	Unemployment insurance (UI)	Min. 26 weeks, last 2 tax years	GBP 73.10/week (flat-rate)	59	6	Suspension of payment	36
	Unemployment assistance (UA)	n/a	Same as UI amount	n/a	Unlimited	n/a	n/a
Australia	Unemployment assistance (UA)	n/a	AUD 538.8/ fortnight (flat-rate)^d^	52	Unlimited	Termination of payment	n/a
USA	Unemployment insurance (UI)^ef^	20 weeks, plus min. earnings requirement	n/a	41	20	Termination of payment	n/a
	Social assistance (SA)^f^	USD 848/month (max. median income)^g^	USD 486/month (median)^h^	n/a	12-60^1^	Adult portion of benefit - termination of payment^i^	Until compliance or 1 month - permanent^i^

Note:

a Initial net replacement rate in unemployment is the percentage of income maintained after 1 month of unemployment for one adult with dependent children, with an average wage; calculation includes social assistance benefits and housing benefits (OECD, 2021);

b The value is augmented by 20% of daily gross earnings;

c The annual base amount is NOK 93,634 (2019);

d Plus a tax-exempt energy supplement of AUD 8.80 per fortnight;

e Data reported reflect the UI benefit scheme for Michigan;

f Information refer to state-level data on SA scheme ‘Temporary Assistance for Needy Families’ (TANF) (Shantz *et al*., 2020: Table II.A.4 pp.124-125; Table IV.C.1 pp. 203-207; Table L3: pp. 243-245; Table L7: pp. 263-274);

g Value calculated for a single parent with two children;

h Value calculated for a family with no income;

i Reported minimum and maximum values from state-level data.Source: International Social Security Association and U.S. Social Security Administration (2018a, 2018b, 2019); OECD (2018, 2019, 2021); MISSOC (2019), Shantz *et al*. (2020) and Nordic Health and Welfare Statistics (2021).

**Table 2:** Overview of the study design typology based on the studies included in the scoping review

Study design type	Description	Issues for identification of causal effects
*Non-experimental study designs:*		
1a. Descriptive analysis, Ordinary Least Squares (OLS), logistic or probit regression	Bivariate analyses and multivariable regression models relying on standard covariate adjustment to control for potential confounders	Omission of unobserved confounders which correlate with sanction risks and relevant outcomes may bias estimations of sanction effects
1b. Survival models, fixed and random effects models	More complex models which may control for some unmeasured confounding along with that due to covariates	Issues of residual confounding and reverse causation (endogeneity) may remain
1c. Propensity Score Matching (PSM)	Using selection on observables to estimate the probability of exposure or treatment conditioned on measured confounders	Potential issues of residual and unmeasured confounding
*Quasi-experimental study designs:*		
2. Difference-in-differences (DiD), Interrupted Time Series (ITS), Regression Discontinuity (RD), Instrumental Variables (IV)	Using exogenous variation occurring ‘naturally’ in the data to estimate causal effect	Rely on strong assumptions (e.g. time-invariant confounding, continuity of the assignment variable continuity, association of the instrument with the outcome exclusively through the treatment variable) which are difficult to test although various analyses may give additional support. Some potential issues of unmeasured confounding remain.
*Experimental study designs:*		
3. Random assignment, Randomised Controlled Trial (RCT)	Exploit random assignment of individuals to a treatment and a control group to effectively account for sources of selection bias	Considered as the gold standard for the identification of causal effects

**Table 3:** Overview of the studies for labour market and wider outcomes

Study characteristics	Total sample			Labour market outcomes			Wider outcomes	
	n	%		n	%		n	%
*All^a^*	*103*	100		*68*	*66.0*		*35*	*34.0*
Period covered by study^b^								
1980s	3	2.9		1	1.5		2	5.7
1990s	60	58.3		36	52.9		24	68.6
2000s	34	33.0		26	38.2		8	22.9
2010s	6	5.8		5	7.4		1	2.8
Publication period								
1990s	2	1.9		2	2.9		-	-
2000s	59	57.3		32	47.1		27	77.1
2010s	42	40.8		34	50.0		8	22.9
Selected countries/regions								
USA	65	63.1		35	51.5		30	85.7
Australia	1	1.0		-	-		1	2.9
UK	8	7.7		4	5.9		4	11.4
Continental Europe	22	21.4		22	32.3		-	-
Nordic countries	7	6.8		7	10.3		-	-
Target population^c^								
Low-income families/lone parents	66	63.5		36	52.2		30	85.7
Unemployed people	37	35.5		32	46.4		5	14.3
People with a disability	1	1.0		1	1.4		-	-
Type of programme^c^								
TANF/AFDC benefits^d^	64	61.5		34	49.3		30	85.7
Unemployment insurance	24	23.1		20	29.0		4	11.4
Unemployment assistance	15	14.4		14	20.3		1	2.9
Disability benefits	1	1.0		1	1.4		-	-
Sanction effect^e^								
Take-up	3	2.9		3	4.4		-	-
Threat	9	8.7		8	11.8		1	2.9
Warning	1	1.0		1	1.5		-	-
Imposed	83	80.6		50	73.5		33	94.2
Multiple	5	4.9		4	5.9		1	2.9
Not known	2	1.9		2	2.9		-	-
Exposure^f^								
Full sanctions	40	38.8		29	42.7		11	31.4
Partial sanctions	18	17.5		16	23.5		2	5.7
Full or partial sanctions	39	37.9		17	25.0		22	62.9
Other	6	5.8		6	8.8		-	-
Type of sanction indicator								
Individual-level	69	67.0		51	75.0		18	51.4
Area-level	34	33.0		17	25.0		17	48.6
Unit of analysis								
Individual-level	90	87.4		62	91.2		28	80.0
Area-level	13	12.6		6	8.8		7	20.0
Type of data								
Administrative data	48	46.6		39	57.4		9	25.7
Survey data	28	27.2		13	19.1		15	42.8
Linked admin-survey data	23	22.3		13	19.1		10	28.6
Other	4	3.9		3	4.4		1	2.9

a The number in each column exceeds the number of studies in the analytical sample (n=94) due to 9 publications reporting both labour market and wider outcomes;

b Study period refers to the onset of the period covered by a study when this encompasses more than one decade;

c The information for ‘Target population’ and ‘Type of programme’ refers to n=104 due to a study reporting outcomes for two target populations exposed to two policy programmes (National Audit Office, 2016b [61]);

d TANF is defined as ‘Temporary Assistance for Needy Family’, means-tested assistance benefits introduced by the US Federal Government in 1996 to replace the prior grant programme ‘Aid to Families with Dependent Children’ (AFDC);

e A definition of sanction effect is provided in Section 3;

f In the US, full sanctions also include full-family sanctions imposed to low-income/lone-parent households in receipt of TANF benefits for work-related non-compliance reported by the head of the household or other adult members.

**Table 4:** Summary of results^a^ for labour market and wider outcomes by main study design

Study characteristics	Non-experimental design									Quasi-experimental design									Experimental design								Tot (n)
	Increase^b^			No effect^b,c^			Decrease^b^			Increase^b^			No effect^b,c^			Decrease^b^			Increase^b^			No effect^b,c^			Decrease^b^		
	n	%		n	%		n	%		n	%		n	%		n	%		n	%		n	%		n	%	
*All*	74	29.0		75	30.0		37	14.5		15	5.9		18	7.1		9	3.6		13	5.1		8	3.2		4	1.6		253
Panel (a): Labour market outcomes																												
Employment	14	31.8		11	25.0		5	11.3		3	6.8		2	4.6		2	4.6		7	15.9		–	–		–	–		44
Job stability	3	21.4		1	7.2		4	28.5		3	21.4		2	14.3		1	7.2		–	–		–	–		–	–		14
Job quality	1	9.1		3	27.3		5	45.4		–	–		1	9.1		1	9.1		–	–		–	–		–	–		11
Non-employment/ inactivity	20	55.6		7	19.4		–	–		5	13.9		3	8.3		–	–		1	2.8		–	–		–	–		36
Long-term non-em-ployment/inequality	–	–		1	33.3		–	–		1	33.3		–	–		–	–		–	–		1	33.3		–	–		3
Benefit receipt	1	4.8		4	19.0		5	23.9		2	9.5		–	–		2	9.5		–	–		3	14.3		4	19.0		21
Long-term benefit receipt	1	16.7		3	50.0		2	33.3		–	–		–	–		–	–		–	–		–	–		–	–		6
Earnings/income	1	3.6		7	25.0		9	32.2		–	–		2	7.1		3	10.7		4	14.3		2	7.1		–	–		28
*Total*	41	25.1		37	22.7		30	18.4		14	8.6		10	6.1		9	5.5		12	7.4		6	3.7		4	2.5		163
Panel (b): Wider outcomes																			
Material hardship	17	48.6		16	45.7		2	5.7		–	–		–	–		–	–		–	–		–	–		–	–		35
Health problems	6	50.0		5	41.7		1	8.3		–	–		–	–		–	–		–	–		–	–		–	–		12
Health insurance	–	–		4	66.7		2	33.3		–	–		–	–		–	–		–	–		–	–		–	–		6
Child well-being	1	12.5		2	25.0		1	12.5		–	–		4	50.0		–	–		–	–		–	–		–	–		8
Child maltreatment	4	36.4		4	36.4		–	–		–	–		–	–		–	–		1	9.1		2	18.1		–	–		11
Child education	–	–		1	50.0		1	50.0		–	–		–	–		–	–		–	–		–	–		–	–		2
Demographic outcomes	1	10.0		6	60.0		–	–		–	–		3	30.0		–	–		–	–		–	–		–	–		10
Vulnerable status	1	100		–	–		–	–		–	–		–	–		–	–		–	–		–	–		–	–		1
Crime	–	–		–	–		–	–		1	50		1	50.0		–	–		–	–		–	–		–	–		2
Compliance	1	100		–	–		–	–		–	–		–	–		–	–		–	–		–	–		–	–		1
Other	2	100		–	–		–	–		–	–		–	–		–	–		–	–		–	–		–	–		2
*Total*	33	36.7		38	42.2		7	7.8		1	1.1		8	8.9		–	–		1	1.1		2	2.2		–	–		90

a Information is reported at the lowest level of aggregation, for outcome measures (n=253);

b The significance level for the reported results is p<0.05; to aid interpretation of the direction of results, we report row percentages referring to total observations for each outcome. When significant, the sign of the estimated parameter for some outcome measures are inverted for ease of interpretation. For example, for measures concerning job stability, if the study reports a significant increase in the risk of entry into short-term jobs, then this is reported as a significant decrease;

c The category ‘no change’ includes results from descriptive studies for which the level of significance was not reported (not applicable).
