# Supplementary material for: The Impacts of Benefit Sanctions: A Scoping Review of the Quantitative Research Evidence
Source: J Soc Policy. Author manuscript; Available in PMC 2022 Aug 22. (PMC7613403; doi:10.1017/S0047279421001069)
Supplement: Supplementary material 2 [file EMS152035-supplement-Supplementary_material_2.docx]

## Tables to be included as Supplementary Material

TABLE A1. Additional information on the social/unemployment protection systems and sanction policies by benefit scheme and selected countries and regions, 2019

| ***Nordic countries:*** |
| --- |
| **Denmark:** The voluntary Unemployment Insurance (UI) (*Arbejdsløshedsdagpenge og andre Akasseydelser*) scheme covers both employees and the self-employed. The scheme is financed by employees’ contributions through membership payments to an approved insurance fund and the State through general taxation. The regulation of the scheme was partly modified with the implementation of the unemployment benefit reforms of 2017 and 2018. One of the main changes of the reforms concerned the qualifying criteria for UI benefits which are based upon ‘income’ rather than ‘hours worked’. Behavioural requirements include registration with the public employment service, weekly access to the ‘jobnet’ online portal to confirm work availability and attendance of an interview with the insurance fund every 3 months and on request after 6 months. UI benefits are subject to taxation. Recipients do not pay the social security contribution but do pay contributions to the UI fund including early retirement insurance contributions (a fixed amount). UI benefit recipients can receive other benefits (e.g. disability pension) but duration is limited; benefits are taken into account for means testing of social assistance. Recipients are allowed to work but earnings reduce the amount of benefits on an hour-to-hour basis. |
| **Finland:** The Unemployment Insurance (UI) benefit include two components: (i) a compulsory basic flat-rate benefit (*peruspäiväraha*) and (ii) a voluntary earnings-related benefit (*ansiopäiväraha*), based on previous income, covering both employees and the self-employed. The labour market subsidy (työmarkkinatuki) is a means-tested Unemployment Assistance (UA) scheme consisting of a flat-rate benefit for residents in Finland who do not qualify for UI benefits. The basic flat-rate benefit is funded by the state and employees, while the earnings-related benefit is funded by the state, employers, employees and unemployment funds. The labour market subsidy is financed by taxes. Since 2018 behavioural requirements for both UI and UA benefits are assessed every 3 calendar months and include: being employed at least for 18 hours, earning at least 23% of the monthly earnings (for the self-employed), participating in employment promotion measures for at least 5 days. People with disabilities and family carers are exempted from these requirements. Sanctions are imposed when a claimant: (i) refuses to taken on work or declines training without a justifiable reason; (ii) refuses to cooperate for the preparation of a job-seeking plan; (iii) resigns from a job without an acceptable reason or loses a job due to misconduct. Benefits are taxable and subject to health insurance contribution. Recipients may receive housing allowance and social assistance. |
| **Norway:** The Unemployment Insurance (UI) benefit is a contributory scheme called ‘unemployed benefit under the National Insurance Scheme’ (*dagpenger under arbeidsløshet)*, funded by taxes and the National Budget. Members of the National Insurance Scheme are eligible for UI benefits, i.e. all persons who are either resident or working as employees. Benefit claimants must be capable of working, registered with the labour and welfare service and willing to take any employment anywhere in Norway. Entitlement to UI benefits is granted even when the claimant is not able to meet the availability requirements due to health or care duties. Benefit sanctions are imposed for resigning from a job without acceptable reason, refusing to take a suitable job offer or refusing to participate in labour market activities. UI benefits are taxable as personal income and subject to social security contribution. They cannot be combined with other benefits (e.g. disability benefits, maternity or parental benefit). Earnings from work lead to a proportional reduction of UI benefits. |
| **Sweden:** The Unemployment Insurance (UI) scheme is composed of two parts: (i) voluntary income-related unemployment insurance *(Arbetslöshetsförsäkring inkomstrelaterad)*, financed by employers’ contributions and unemployment insurance funds; (ii) basic unemployment insurance (Arbetslöshetsförsäkring grundnivå), financed by employers’ contributions covering those not voluntarily insured and providing a flat-rate benefit. The benefit claimant must be: (i) capable of working at least 3 hours daily (weekly average 17 hours); (ii) registered at the public employment service; (iii) a member of an unemployment insurance fund for at least 12 months. Reasons for sanctions include neglecting a job-seeking task; unjustified refusal of suitable job offer or of a referral to a labour market programme; leaving a job without an acceptable reason or being dismissed on the grounds of unacceptable behaviour. Both income-related and basic UI benefits are taxable, are compatible with family and housing benefits and are taken into account when assessing means tests for both housing and social assistance benefits. Earnings from paid employment entails a proportional reduction of UI benefits. |
| ***Continental European countries:*** |
| **Belgium:** *Assurance chômage/werkloosheidsverzekering* is a compulsory unemployment insurance scheme funded by contributions from employers and employees. Administrative sanctions are issued by the Unemployment Office and imposed in case of non-cooperation during specific procedures of active job search. Severe sanctions involve the termination of benefit payment and obligation to re-apply. Unemployment benefits can be combined with cash benefits in specific circumstances (e.g. accident at work; occupational disease) and child benefits. Unemployment benefits are also compatible with earnings from paid employment (up to 130% of the maximum daily amount of the unemployment benefit of a person with dependent children) or casual activity. Benefits are subject to taxation and social security contributions. |
| **Netherlands:** Regulated by the Unemployment Benefit Act (Werkloosheidswet, WW) 1986, the compulsory unemployment insurance scheme is funded by employer contributions and includes two types of benefits: (i) a short-term flat-rate benefit (3 months) and (ii) a medium-term earnings-related benefit (4 months up to 38 months which starting from 2015 was incrementally reduced to a maximum of 24 months as of 2019). Benefit claimants must be: (i) involuntary unemployed; (ii) lost at least 5 hours or 50% of their weekly working hours; (iii) registered with the Institute for Employee Benefit Schemes (UWV); (IV) capable and available for work; and (V) reside in the Netherlands. Sanctions are imposed by the social security agency providing the benefits on a contractual basis and involve circumstances such as not applying for jobs, failure to take a suitable job offer or failure to cooperate with the public employment service. |

TABLE A1. Continued

| In principle unemployment benefits can be combined with other social benefits except for statutory old-age benefits. They can also be combined with earnings, although 70% of the income earned is deducted from the unemployment benefit. Benefits are subject to taxation and social security contributions. |
| --- |
| **Germany:** Unemployment insurance (*Arbeitslosenversicherung*) is a compulsory contribution-funded non-taxable scheme regulated by the 1997 Social Code Book III (*Dritte Buch* *Sozialgesetzbuch, SGB III*) while social assistance benefits for jobseekers (*Grundsicherung für Arbeitsuchende*) are a tax-financed scheme regulated by the 2003 Social Code Book II (Zweites Buch *Sozialgesetzbuch, SGB II*), also known as ‘Hartz IV’, the fourth stage of the Hartz reforms (Hartz I-IV) which were implemented in Germany between 2003 and 2005. As a result of ‘Hartz IV’ reform, unemployment assistance and social assistance for those who are able to work were combined into one means-tested non-taxable benefit: the basic jobseekers allowance. UI benefits are not compatible with other social security benefits; income from any part-time work (less than 15 hours per week) reduces entitlement to unemployment benefit. The beneficiary is not required to pay social security contributions. However, the Federal Employment Agency pays contributions for retirement, sickness and long-term care insurance for the beneficiaries of UI benefits. Sanctions are imposed on claimants who voluntarily leave their job without good cause, refuse to take up a reasonable job offer or participate in job-search activities without a justification, or makes insufficient effort to find work. |
| **Switzerland:** The compulsory Unemployment Insurance (*Assurance Chômage/Arbeitslosenversicherung*) scheme covers the risk of both total and partial employment for employees residing in Switzerland, except for self-employed individuals. It is financed by both contributions and taxes. There is no unemployment assistance scheme at federal level. Benefits are subject to taxation. Cantonal and regional unemployment funds (public funds), and approved private unemployment funds administer the program for cantons or regions and for certain professional groups. Regional placement offices and government approved employment agencies assist unemployed persons in finding employment. Sanctions are imposed to claimants who voluntarily leave their job without good cause, make insufficient effort to find work, do not observe the instructions of the relevant authority, refuse a suitable job, does not take part or leave an active labour market programme without good cause. |
| **Hungary:** The Unemployment Insurance (UI) benefit (*Álláskeresési járadék*) scheme covers both employed and self-employed individuals (except employed pensioners) and is regulated by the 1991 Act IV on Promoting Employment and Providing for the Unemployed. The scheme is financed by employers’ and employees’ contributions, and central state budget subsidies. Eligible claimants must be registered jobseekers and cooperate with both county and local unemployment offices. The benefit cannot be combined with any gainful activity, except for occasional and short-term (up to 120 days) employment, during which the benefit is suspended. The benefit is subject to taxation and social security benefit contributions, such as pension insurance, but not health contribution. |
| ***English-speaking countries:*** |
| **United Kingdom:** Unemployment benefits are regulated by the Jobseeker Act 1995 and the Welfare Reform Act 2012; the universal credit is being gradually introduced across the United Kingdom and will replace a number of existing benefits with a single monthly payment by 2024. *Contribution-based Jobseekers' Allowance (JSA*) is a compulsory social insurance scheme for all employed and some self-employed persons financed by employee and employer contributions. Benefits are flat-rate*. Income-based Jobseekers' Allowance* is a social assistance scheme, tax financed and with means-tested flat-rate benefits. Both contribution- and income-based JSA are subject to taxation. Unemployment benefits can be combined with other social security benefits (e.g. Child Benefit) or with paid employment of less than 16 hours per week. Sanctions are imposed on people who fail to show they had just cause for leaving a job voluntarily, refuse or fail to comply with a reasonable a direction from their employment officer, loose their job due to misconduct or refuse an offer of suitable work. |
| **Australia:** Regulated by the 1991 law on social security, the Newstart Allowance (NSA) is payable to those 22 years or older who actively seek paid work, and participate in government-approved activities designed to facilitate entry into employment, unless temporarily exempted. The tax-financed programme is run by the Department of Social Services which is responsible for policy development and provides general supervision of cash benefits, while the Department of Human Services delivers social and health-related payments and services. NSA payments are subject to taxation, although tax rebates operate, and can be combined with other social security benefits such as childcare benefits. NSA payments are not compatible with full-time employment. |
| **USA:** Unemployment Insurance (UI) benefits are regulated by the 1935 Federal law on social security and cover public- and private-sector employees, except for self-employed persons and employees of the agriculture sector. The tax-financed federal programme is administered by the Department of Labour while individual state unemployment programmes and benefits are administered and paid by state workforce agencies. Sanctions are imposed to persons who voluntarily leave their job without good cause, are discharged from employment for misconduct or refuse an offer of suitable work.  The Personal Responsibility and Work Opportunity Reconciliation Act 1996 (PRWORA) created the Temporary Assistance for Needy Families (TANF) social-assistance block grant by replacing the Aid to Families with Dependent Children (AFDC) programme, which provided mean-tested public assistance to low-income families with children since its creation as part of the New Deal in 1935. Federal aid receipt was limited to 5 years and workfare was mandated for those who do not find paid work within 2 years. States were given significant discretionary power in setting programme rules (e.g. work requirements and financial sanctions for non-compliance with programme rules). |

Source: MISSOC (2019), OECD (2019), Shantz *et al.* (2020) and Nordic Health and Welfare Statistics (2021).

TABLE A2. Summary of search strategies and records retrieved

| Database name and coverage | Interface | Database dates | Records retrieved |
| --- | --- | --- | --- |
| ASSIA | Proquest | 1987- | 324 |
| BEI (British Education Index) | EBSCO | 1929- | 56 |
| EconLit^a^ | EBSCO | 1886- | 1033 |
| EconLit^b^ | EBSCO | 1886- | 1424 |
| ERIC | EBSCO | 1966- | 492 |
| PsycINFO | EBSCO | 1698- | 821 |
| MEDLINE^c^ | OVID | 1996- | 534 |
| SCOPUS | Elsevier | 2004- | 2365 |
| SocINDEX | EBSCO | 1908- | 2580 |
|  |  |  |  |
| Total |  |  | 9629 |
|  |  |  |  |
| After duplicates removed |  |  | 7169 |

Notes: **^a^** The searches for both this database and all the remaining unmarked databases, including Medline, were conducted in March 2019; ^b^ A revised search for EconLit was conducted in June 2019 in order to integrate the search terms relating to ‘unemployment insurance’ which were not previously included; **^c^** No revisions from 1996 to February Week 4 2019.

TABLE A3. Search strategies

| Search | Terms |
| --- | --- |
|  | *ASSIA* |
| Set 1 | (noft(sanction*) OR noft(penalt*) OR noft(punishment*) OR noft(punitive) OR noft(monitoring) OR noft(exclusion) OR noft(leaving) OR noft(exiting)) OR noft(exit) OR noft(austerity) OR noft(conditionality) OR noft("welfare conditionality") OR noft ("welfare sanction") |
| Set 2 | (noft(claimant*) OR noft("job seeker*") OR noft(unemployed) OR noft("welfare recipient*") OR noft(recipient*) OR noft(unemployment) OR noft(family) OR noft(families) OR noft(child*) OR noft(youth) OR noft(jobless) OR noft (sick) OR noft (sickness) or noft (disabled) OR noft (disability) OR noft (impaired) OR noft(incapacity) OR noft(parent*) OR noft(lone) OR noft(singlel)) |
| Set 3 | (noft("TANF") OR noft("welfare to work") OR noft("public assistance") OR noft("employment and support allowance") OR noft("individual re-integration agreement") OR noft(monetary NEAR/2 incentive) OR noft("monetary N/3 incentive") OR noft("monetary benefit") OR noft("social assistance") OR noft("work first strateg*") OR noft("incapacity benefit*") OR noft("disability living allowance") OR noft("Personal Responsibility and Work Opportunity Reconciliation Act") OR noft("Active labo?r market polic*") OR noft("Active labo?r market program*") OR noft("agenda 2010") OR noft("domestic purposes benefit") OR noft("america works") OR noft("cash benefit*") OR noft("cash incentive") OR "government intervention*" OR noft("government program*") OR noft("income benefit*") OR noft("income supplement*") OR noft("job seeker*") OR noft("job seekers allowance") OR noft("public welfare reform*") OR noft("tax credit*") OR noft("universal credit") OR noft("financial benefit*") OR noft("conditional benefit*") OR noft("work program*") OR noft("social security reform*") OR noft("claimant*") OR noft("welfare reform*") OR noft("benefit cap") OR noft("welfare conditionality") OR noft("social protection scheme*") OR noft("temporary assistance to needy families")) |
| Set 4 | All sets combined |
|  |  |
|  | *EBSCO – for all databases* |
| S1 | "help to work program" |
| S2 | "canada health and social transfer" |
| S3 | "Personal Responsibility and Work Opportunity Reconciliation Act" |
| S4 | "Active labo?r market polic*" |
| S5 | Active labo?r market program* OR active labo?r participation |
| S6 | "Agenda 2010" |
| S7 | "domestic purposes benefit" |
| S8 | "America works" |
| S9 | cash benefit* |
| S10 | cash incentives |
| S11 | "government intervention*" |
| S12 | "government program*" |
| S13 | "income benefit" |
| S14 | "income support" |
| S15 | "income supplement*" |
| S16 | "job seeker allowance*" |
| S17 | "public welfare reform*" |
| S18 | "tax credit" |

TABLE A3. Continued

| Search | Terms |
| --- | --- |
| S19 | "universal credit" |
| S20 | "financial benefit*" |
| S21 | "conditional benefits" |
| S22 | "work program*" |
| S23 | "social security reform*" |
| S24 | "welfare reform*" |
| S25 | "benefit cap" |
| S26 | welfare conditionality |
| S27 | ((DE "PUBLIC welfare policy") OR (DE "WELFARE recipients")) OR (DE "WELFARE state") |
| S28 | "public assistance" |
| S29 | DE "SOCIAL security" |
| S30 | "social protection scheme" |
| S31 | "Temporary Assistance to Needy Families" OR tanf |
| S32 | "welfare to work" |
| S33 | "Employment and Support Allowance" |
| S34 | hilfe zum arbeit OR hilfe zum lebensunterhalt |
| S35 | "individual re-integration agreement" |
| S36 | monetary N3 incentive |
| S37 | "monetary benefit*" |
| S38 | "social assistance" |
| S39 | "work first strateg*" |
| S40 | "disability living allowance" |
| S41 | basic income |
| S42 | welfare funds |
| S43 | ontario works |
| S44 | mandatory employment |
| S45 | new start allowance |
| S46 | jobbskatteavdraget |
| S47 | workfare |
| S48 | disability benefit* |
| S49 | incapacity benefit* |
| S50 | S1 OR S2 OR S3 OR S4 OR S5 OR S6 OR S7 OR S8 OR S9 OR S10 OR S11 OR S12 OR S13 OR S14 OR S15 OR S16 OR S17 OR S18 OR S19 OR S20 OR S21 OR S22 OR S23 OR S24 OR S25 OR S26 OR S27 OR S28 OR S29 OR S30 OR S31 OR S32 OR S33 OR S34 OR S35 OR S36 OR S37 OR S38 OR S39 OR S40 OR S41 OR S42 OR S43 OR S44 OR S45 OR S46 OR S47 OR S48 OR S49 |
| S51 | sanction* |
| S52 | penalt* |
| S53 | punishment* |
| S54 | punitive |
| S55 | welfare conditionality |
| S56 | "welfare sanction" |
| S57 | austerity |
| S58 | exclusion OR exit* OR leaving or loss |
| S59 | monitoring |
| S60 | S51 OR S52 OR S53 OR S54 OR S55 OR S56 OR S57 OR S58 OR S59 |

TABLE A3. Continued

| Search | Terms |
| --- | --- |
| S61 | claimant* or parent* or family or families or child* or youth or lone or single or disabled or disability or impaired or incapacity or sick or sickness |
| S62 | job seeker* |
| S63 | jobless* |
| S64 | recipient* |
| S65 | "welfare recipient" |
| S66 | unemployed or unemployment |
| S67 | S61 OR S62 OR S63 OR S64 OR S65 OR S66 |
|  |  |
|  | *Medline* |
| 1 | "welfare benefits".ab,ti. |
| 2 | help to work program.ab,ti. |
| 3 | (canada health and social transfer).ab,ti. |
| 4 | (Personal Responsibility and Work Opportunity Reconciliation Act).ab,ti. |
| 5 | "Active labo?r market polic*".ab,ti. |
| 6 | labo?r force participation.ab,ti. |
| 7 | "Active labo?r market program*".ab,ti. |
| 8 | Agenda 2010.ab,ti. |
| 9 | domestic purposes benefit.ab,ti. |
| 10 | america works.ab,ti. |
| 11 | cash benefit.ab,ti. |
| 12 | cash incentive.ab,ti. |
| 13 | government intervention.ab,ti. |
| 14 | "Government program*".ab,ti. |
| 15 | "income benefit*".ab,ti. |
| 16 | income support.ab,ti. |
| 17 | income supplement.ab,ti. |
| 18 | job seeker allowance.ab,ti. |
| 19 | "public welfare reform*".ab,ti. |
| 20 | "tax credit*".ab,ti. |
| 21 | basic income.ab,ti. |
| 22 | universal credit.ab,ti. |
| 23 | financial benefit*.ab,ti. |
| 24 | conditional benefits.ab,ti. |
| 25 | "work program*".ab,ti. |
| 26 | social security reform*.ab,ti. |
| 27 | "welfare reform*".ab,ti. |
| 28 | welfare fund.ab,ti. |
| 29 | benefit cap.ab,ti. |
| 30 | welfare conditionality.ab,ti. |
| 31 | Social Welfare/ |
| 32 | public assistance/ |
| 33 | Social Security/ |
| 34 | "social protection scheme*".ab,ti. |
| 35 | Temporary Assistance to Needy Families.ab,ti. |
| 36 | TANF.ab,ti. |

TABLE A3. Continued

| Search | Terms |
| --- | --- |
| 37 | welfare to work.ab,ti. |
| 38 | public assistance.ti,ab. |
| 39 | (Employment and Support Allowance).ab,ti. |
| 40 | hilfe zum arbeit.ab,ti. |
| 41 | hilfe zum lebensunterhalt.ab,ti. |
| 42 | revenu minimum d'insertion.ab,ti. |
| 43 | individual re-integration agreement.ab,ti. |
| 44 | (monetary adj 2 incentive*).ab,ti. |
| 45 | "monetary benefit*".ab,ti. |
| 46 | monetary support.ab,ti. |
| 47 | social assistance.ab,ti. |
| 48 | "work first strateg*".ab,ti. |
| 49 | "incapacity benefit*".ab,ti. |
| 50 | disability living allowance.ab,ti. |
| 51 | disability benefit*.ab,ti. |
| 52 | workfare.ab,ti. |
| 53 | new start allowance.ab,ti. |
| 54 | jobbskatteavdraget.ab,ti. |
| 55 | mandatory employment.ab,ti. |
| 56 | Ontario works.ab,ti. |
| 57 | "sanction*".ab,ti. |
| 58 | "penalt*".ab,ti. |
| 59 | "punishment*".ab,ti. |
| 60 | punitive.ab,ti. |
| 61 | welfare conditionality.ab,ti. |
| 62 | conditionality.ab,ti. |
| 63 | "welfare sanction*".ab,ti. |
| 64 | austerity.ab,ti. |
| 65 | loss.ab,ti. |
| 66 | exclusion.ab,ti. |
| 67 | exit*.ab,ti. |
| 68 | monitoring.ab,ti. |
| 69 | leaving.ab,ti. |
| 70 | "job seeker*".ab,ti. |
| 71 | jobless.ab,ti. |
| 72 | "recipient*".ab,ti. |
| 73 | "welfare recipient*".ab,ti. |
| 74 | unemployed.ab,ti. |
| 75 | unemployment.ab,ti. |
| 76 | (claimant* or parent* or family or families or child* or youth or lone or single or disabled or disability or impaired or incapacity or sick or sickness).ab,ti. |
| 77 | 1 or 2 or 3 or 4 or 5 or 6 or 7 or 8 or 9 or 10 or 11 or 12 or 13 or 14 or 15 or 16 or 17 or 18 or 19 or 20 or 21 or 22 or 23 or 24 or 25 or 26 or 27 or 28 or 29 or 30 or 31 or 32 or 33 or 34 or 35 or 36 or 37 or 38 or 39 or 40 or 41 or 42 or 43 or 44 or 45 or 46 or 47 or 48 or 49 or 50 or 51 or 52 or 53 or 54 or 55 or 56 |

TABLE A3. Continued

| Search | Terms |
| --- | --- |
| 78 | 57 or 58 or 59 or 60 or 61 or 62 or 63 or 64 or 65 or 66 or 67 or 68 or 69 |
| 79 | 70 or 71 or 72 or 73 or 74 or 75 or 76 |
| 80 | 77 and 78 and 79 |
|  |  |
|  | SCOPUS |
| 1 | ( ( ( TITLE-ABS-KEY ( "Ontario Works" ) OR TITLE-ABS-KEY ( "welfare benefits" ) OR TITLE-ABS-KEY ( "exit to work" ) OR TITLE-ABS-KEY ( "unemployment insurance" ) OR TITLE-ABS-KEY ( "Help to work program*" ) ) OR ( "Personal Responsibility and Work Opportunity Reconciliation Act" ) ) OR ( TITLE-ABS-KEY ( "Active labo?r market polic*" ) ) OR ( TITLE-ABS-KEY ( active AND labo?r AND market AND program* ) ) OR ( TITLE-ABS-KEY ( "Agenda 2010" ) ) OR ( TITLE-ABS-KEY ( "le new deal" ) ) OR ( TITLE-ABS-KEY ( labo?r AND force AND participation ) OR ( TITLE-ABS-KEY ( "domestic purposes benefit" ) ) ) OR ( ( TITLE-ABS-KEY ( "America works" ) ) OR ( TITLE-ABS-KEY ( cash AND benefit* ) ) OR ( TITLE-ABS-KEY ( cash AND incentive* ) ) OR ( TITLE-ABS-KEY ( "government intervention*" ) ) OR ( TITLE-ABS-KEY ( "government program*" ) ) OR ( TITLE-ABS-KEY ( "income benefit" ) ) OR ( TITLE-ABS-KEY ( "income support" ) ) OR ( TITLE-ABS-KEY ( "income supplement*" ) ) OR ( TITLE-ABS-KEY ( "job seeker allowance*" ) ) ) OR ( ( TITLE-ABS-KEY ( "tax credit" ) ) OR ( TITLE-ABS-KEY ( "universal credit" ) ) OR ( TITLE-ABS-KEY ( "financial benefit*" ) ) OR ( TITLE-ABS-KEY ( "conditional benefits" ) ) OR ( TITLE-ABS-KEY ( "work program*" ) ) OR ( TITLE-ABS-KEY ( workfare ) ) OR ( TITLE-ABS-KEY ( "social security reform*" ) ) OR ( TITLE-ABS-KEY ( "welfare reform*" ) ) OR ( TITLE-ABS-KEY ( "benefit cap" ) ) ) OR ( ( TITLE-ABS-KEY ( welfare AND conditionality ) ) OR ( TITLE-ABS-KEY ( "public assistance" ) ) OR ( TITLE-ABS-KEY ( "social protection scheme" ) ) OR ( TITLE-ABS-KEY ( "Temporary Assistance to Needy Families" OR tanf ) ) OR ( TITLE-ABS-KEY ( "welfare to work" ) ) OR ( TITLE-ABS-KEY ( "Employment and Support Allowance" ) ) OR ( TITLE-ABS-KEY ( hilfe AND zum AND arbeit OR hilfe AND zum AND lebensunterhalt ) ) OR ( TITLE-ABS-KEY ( "individual re-integration agreement" ) ) OR ( TITLE-ABS-KEY ( "monetary benefit*" ) ) OR ( TITLE-ABS-KEY ( "monetary incentive*" ) ) ) OR ( ( TITLE-ABS-KEY ( "social assistance" ) ) OR ( TITLE-ABS-KEY ( "work first strateg*" ) ) OR ( TITLE-ABS-KEY ( "incapacity benefit" ) ) OR ( TITLE-ABS-KEY ( "disability living allowance" ) ) OR ( TITLE-ABS-KEY ( "Canada health and social transfer" ) ) OR ( TITLE-ABS-KEY ( "Active labo?r force prticipation)) or (TITLE-ABS-KEY(public welfare reform*)) or (TITLE-ABS-KEY(basic income)) or (TITLE-ABS-KEY(welfare fund*)) (TITLE-ABS-KEY(" revenue AND minimum AND d'insertion ")) or (TITLE-ABS-KEY(" monetary AND support ")) or (TITLE-ABS-KEY(" disability AND living AND allowance* ) ) OR ( TITLE-ABS-KEY ( "disability benefit" ) ) OR ( TITLE-ABS-KEY ( "new start allowance" ) ) OR ( TITLE-ABS-KEY ( "jobbskatteavdraget" ) ) OR ( TITLE-ABS-KEY ( "mandatory employment" ) ) ) ) |
| 2 | AND ( TITLE-ABS-KEY ( impaired OR incapacity OR parent* OR lone OR single OR sickness OR sick OR family OR families OR child* OR youth OR claimant* OR "job seeker" OR unemployment OR unemployed OR "welfare recipient*" OR jobless* OR recipient* OR disability OR disabled ) ) |
| 3 | AND ( TITLE-ABS-KEY ( sanction* OR punishment* OR penalt* OR punitive OR exit* OR monitoring OR leaving OR exclusion OR austerity OR loss OR conditionality OR "welfare conditionality" OR "welfare sanction" ) ) |

TABLE A3. Continued

| Search | Terms |
| --- | --- |
|  | *EconLit (revised search conducted in June 2019)* |
| S1 | "help to work program" |
| S2 | "canada health and social transfer" |
| S3 | "Personal Responsibility and Work Opportunity Reconciliation Act" |
| S4 | "Active labo?r market polic*" |
| S5 | "Agenda 2010" |
| S6 | "domestic purposes benefit" |
| S7 | "America works" |
| S8 | "cash benefit*" |
| S9 | "cash incentive*" |
| S10 | "government intervention*" |
| S11 | "government program*" |
| S12 | "income benefit*" |
| S13 | "income support" |
| S14 | "income supplement*" |
| S15 | "job seeker allowance*" |
| S16 | "public welfare reform*" |
| S17 | "tax credit*" |
| S18 | "universal credit" |
| S19 | "financial benefit*" |
| S20 | "conditional benefits" |
| S21 | "work program*" |
| S22 | "welfare reform*" |
| S23 | "benefit cap" |
| S24 | "welfare conditionality" |
| S25 | "Temporary Assistance to Needy Families" OR "TANF" |
| S26 | "welfare to work" |
| S27 | "Employment and Support Allowance" |
| S28 | hilfe zum arbeit OR hilfe zum lebensunterhalt |
| S29 | "individual re-integration agreement" |
| S30 | "monetary benefit*" |
| S31 | "social assistance" |
| S32 | "work first strateg*" |
| S33 | "disability living allowance" |
| S34 | "basic income" |
| S35 | "basic income guarantee" |
| S36 | "ontario works" |
| S37 | "mandatory employment" |
| S38 | "new start allowance" |
| S39 | jobbskatteavdraget |
| S40 | workfare |
| S41 | "disability benefit*" |
| S42 | "incapacity benefit*" |

TABLE A3. Continued

| Search | Terms |
| --- | --- |
| S43 | S1 OR S2 OR S3 OR S4 OR S5 OR S6 OR S7 OR S8 OR S9 OR S10 OR S11 OR S12 OR S13 OR S14 OR S15 OR S16 OR S17 OR S18 OR S19 OR S20 OR S21 OR S22 OR S23 OR S24 OR S25 OR S26 OR S27 OR S28 OR S29 OR S30 OR S31 OR S32 OR S33 OR S34 OR S35 OR S36 OR S37 OR S38 OR S39 OR S40 OR S41 OR S42 |
| S44 | (ZU "unemployment insurance") or (ZU "unemployment insurance; severance pay; plant closings") or (ZU "unemployment assistance") |
| S45 | (ZU "welfare, well-being, and poverty: government programs; provision and effects of welfare programs" OR ZU "social security")) |
| S46 | S44 OR S45 |
| S47 | S43 OR S46 |
| S48 | AB sanctions* OR TI sanction* |
| S49 | AB penalt* OR TI penalt |
| S50 | AB punishment* OR TI punishment* |
| S51 | AB punitive OR TI punitive |
| S52 | AB conditionality OR TI conditionality |
| S53 | AB austerity OR TI austerity |
| S54 | TI exclusion OR AB exclusion |
| S55 | AB exit* AND TI exit* |
| S56 | AB leaving OR TI leaving |
| S57 | AB loss AND TI loss |
| S58 | TI monitoring OR AB monitoring |
| S59 | S48 OR S49 OR S50 OR S51 OR S52 OR S53 OR S54 OR S55 OR S56 OR S57 OR S58 |
| S60 | S47 OR S59 |

TABLE A4. Overview of studies on the impacts of benefit sanctions included in the analytical sample by type of outcomes and study design

| Author(s) (year) and  study no. [#]^a^ | Country,  period and  population | Data and  sample size | Intervention  description | Outcome(s), measure(s)  and exposure | Study design and method | Key results and  time horizon of effects^b^ | Summary^c^ |
| --- | --- | --- | --- | --- | --- | --- | --- |
| Panel a1: Labour market outcomes: Non-experimental design | | | | | | | |
| Abbring, van den Berg and van Ours  (2005)  [1] | Netherlands  1992-1993  Unemployment Insurance (UI) claimants | Administrative data: Dutch Social Security Council (SVr)  n = (up to) 40,089 spells;  32,657 individuals | UI system: variation by industry and insurance organisations; Sanctioning rates: increased with 1987 Unemployment Law.  UI benefits:  Wage compensation rate: 70%;  Maximum duration:  6 months to 5 years | *Adult outcome*:  Employment:  1.Entry into employment  *Exposure*:  Imposition of temporary sanctions (91 days) with average ~20% reduction in benefits | Non-experimental:  Timing-of-events: mixed proportional hazards models | Sanctions significantly increase the risk of entry into employment by 58% for men and 67% for women.  *Time horizon*: medium-term | 1.🡩 |
| Acs, Phillips and Nelson (2005)  [2] | USA  1996-2000  Low-income families/lone parents | Survey of Income and Program Participation (SIPP), 1990 and 1996 panels  n = (up to) 11,307 spells for low-income lone mothers | In 1996 the Temporary Assistance for Needy Families (TANF) replaced Aid to Families with Dependent Children (AFDC), a mean-tested public assistance programme for lone parents with low income. Federal aid receipt was limited to 5 years and workfare was mandated for those who do not find paid work within 2 years. States were given significant discretionary power in setting programme rules (e.g. work requirements and financial sanctions for non-compliance with programme rules). | *Adult outcomes*:  Benefits:  1.Entry into benefits  Non-employment/ inactivity  2.Exit from benefits  *Exposure*:  Imposition of a full-family sanction (100%); duration of sanction varies by state | Non-experimental:  Discrete-time competing risks models (multinomial logit models) | Sanctions significantly decrease the risk of entry into benefits by 41% among lone mothers from the post-reform cohort compared to the pre-reform cohort. No significant effects are observed for transitions out of benefits.  *Time horizon*: long-term | 1.🡫  2. ○ |

Notes: ^a^ Study number and reference for each study are reported in Table A4; ^b^ Time horizon of effects is classified as: short-term (0-12 months); medium-term (13-24 months), long-term (25+ months)

^c^ Direction of effect by outcome measure number: 🡩 = significant increase (p<0.05); 🡫 = significant decrease (p<0.05); ○ = no significant change (p>0.05).

TABLE A4. Continued

| Author(s) (year) and  study no. [#]^a^ | Country,  period and  population | Data and  sample size | Intervention  description | Outcome(s), measure(s)  and exposure | Study design and method | Key results and  time horizon of effects^b^ | Summary^c^ |
| --- | --- | --- | --- | --- | --- | --- | --- |
|  |  |  |  |  |  |  |  |
| Ahmad, Svarer and Naveed (2019)  [3] | Denmark  2003-2005  Unemployment Insurance (UI) claimants aged 26-65 years | Administrative data: Danish Register for Evaluation of Marginalization (DREAM) and AMANDA register of sanctions  n = (up to) 219,348 spells;  164,962 individuals | Members of a UI fund qualify for UI payments, partly covered by UI fund and state. Around 80% of the labour force are members of a UI fund.  UI benefits:  Wage compensation rate: 60% (on average);  If UI benefits are stopped, the unemployed can apply for social assistance (means tested): approximately 20% lower than UI benefits. | *Adult outcome*:  Non-employment/ inactivity:  1.Exit from benefits  *Exposure*:  First imposition of a sanction (assumed 100%); aggregate sanction duration (typically either 2-3 days or 3 weeks) | Non-experimental:  Timing-of-events: mixed proportional hazards models | The imposition of a sanction increases the exit rate out of unemployment by 71% for men and 64% for women.  *Time horizon*: long-term | 1.🡩 |
|  |  |  |  |  |  |  |  |

Notes: ^a^ Study number and reference for each study are reported in Table A4; ^b^ Time horizon of effects is classified as: short-term (0-12 months); medium-term (13-24 months), long-term (25+ months)

^c^ Direction of effect by outcome measure number: 🡩 = significant increase (p<0.05); 🡫 = significant decrease (p<0.05); ○ = no significant change (p>0.05).

TABLE A4. Continued

| Author(s) (year) and  study no. [#]^a^ | Country,  period and  population | Data and  sample size | Intervention  description | Outcome(s), measure(s)  and exposure | Study design and method | Key results and  time horizon of effects^b^ | Summary^c^ |
| --- | --- | --- | --- | --- | --- | --- | --- |
|  |  |  |  |  |  |  |  |
| Arni and Schiprowski (2015)  [4] | Switzerland  (Bern, Fribourg, Solothurn, Graubuenden and Tessin cantons)  2010-2014  Unemployment Insurance (UI) claimants, aged 20-55 years | Swiss UI benefits data, 2010-2012; caseworker monitoring database  n = (up to) 76,404 individuals | Unemployment Insurance (UI) system requires minimum 6 months UI taxes in previous 2 years.  UI benefits:  Wage compensation rate: 70-80%;  Potential duration:  2 years.  Those ineligible for UI can receive social assistance (SA). Claimants are required to start job search when they receive notice for current job and evidence set number of monthly job applications. | *Adult outcomes:*  Employment:  1. Entry into employment, 8+ applications per month  Job stability:  2. Entry into unemployment, 8+ applications per month  *Exposure:*  Threat of sanctions through binding job-search requirements;  Sanctions: 100% of benefit (median value: 7 days of UI benefits) | Non-experimental:  Timing-of-events: mixed proportional hazards models with fixed effects | The threat of sanction through the requirement to write 8 or more additional job applications per month increases the probability of finding employment by 34% in the first 6 months. This probability declines to 22% at 24 months. Women and those without a high school diploma were more strongly affected, with a 67% (vs. no significant effect for men) and 38% (vs. no significant effect for higher educated) probability of finding a job at 6 months, respectively. Effects are not significant when the vacancy rate is low (at 6 months). Although job finding increases, job stability, measured as the probability of re-entry into unemployment is lower, with the threat of sanctions increasing the chances of returning to unemployment by 7%. There is also a risk that those with lower skills are pushed into unstable employment; the lowest educated had a 12% probability of returning to unemployment within the first 6 months.  *Time horizon:* short to medium-term | 1.🡩  2.🡩 |

Notes: ^a^ Study number and reference for each study are reported in Table A4; ^b^ Time horizon of effects is classified as: short-term (0-12 months); medium-term (13-24 months), long-term (25+ months)

^c^ Direction of effect by outcome measure number: 🡩 = significant increase (p<0.05); 🡫 = significant decrease (p<0.05); ○ = no significant change (p>0.05).

TABLE A4. Continued

| Author(s) (year) and  study no. [#]^a^ | Country,  period and  population | Data and  sample size | Intervention  description | Outcome(s), measure(s)  and exposure | Study design and method | Key results and  time horizon of effects^b^ | Summary^c^ |
| --- | --- | --- | --- | --- | --- | --- | --- |
| Arni, Lalive and van Ours (2013)  [5] | Switzerland  (7 out of 26 cantons)  2000-2002  Unemployment Insurance (UI) claimants aged 30-55 | Administrative data:  Unemployment Insurance Register (UIR) database,  1998-2003;  Social Security Administration (SSA) database,  1993-2002  n = 23,961 spells | See description provided for Arni and Schiprowski (2015) [4].  Potential duration of UI benefits: 2 years.  Sanctions: 100% of UI benefit; max. duration: 60 work days.  Claimants required to fulfil job-search criteria, participate in ALMP, apply for certain number of jobs per month (typically 10), and meet caseworker at least once a month. | *Adult outcomes:*  Employment:  1. Entry into employment  Job stability:  2. Exit from employment post UI benefits exit  Non-employment/ inactivity  3. Entry into non-employment  4. Exit from non-employment post UI benefits exit  Job quality (earnings)  5. Earnings from re-employment during the first 1 or 24 months from UI benefits exit  *Exposure:*  Receiving a warning (W) letter, implementation (I) of a sanction and threat (T) of sanctions (i.e. measure of how strictly is monitoring of a Public Employment Service (PES) office) | Non-experimental:  Timing-of-events: mixed proportional hazards models | Receiving warning (W) of a sanction or an imposed (I) sanction increases the exit rates from UI benefits to employment by 16%, while an imposed sanction increases the probability of labour market exit by 15% over the 24 months post-unemployment observation window.  While receiving a sanction warning increases the risk of entry into non-employment by 99%, the enforcement of a sanction increases the risk of entering non-employment by 67%. Sanction enforcement also increases the risk of moving of from the first post-benefit non-employment spell by 31%.  Both the warning and the threat of a sanction have a significant and negative effect on earnings with the warning and the threat of sanctions (measured with a one standard deviation increase in the latter case) leading to an increase in the earnings hazard by 0.8% and 2.8% during the first month of employment and by 11% and 4.9% during the 24 months after a UI benefits exit due to employment, respectively. When considering also those who experience temporary exits to non-employment after a UI benefits exit, the warning effect increases by 12% while a significant imposition effect of 11% is observed.  *Time horizon: medium-term* | 1. 🡩 (W)  🡩 (I)  ○ (T)  2. ○ (W)  🡩 (I)  ○ (T)  3. 🡩 (W)  🡩 (I)  ○ (T)  4. ○ (W)  🡩 (I)  ○ (T)  5. 🡫 (W)  ○ (I)  🡫 (T) |

Notes: ^a^ Study number and reference for each study are reported in Table A4; ^b^ Time horizon of effects is classified as: short-term (0-12 months); medium-term (13-24 months), long-term (25+ months)

^c^ Direction of effect by outcome measure number: 🡩 = significant increase (p<0.05); 🡫 = significant decrease (p<0.05); ○ = no significant change (p>0.05).

TABLE A4. Continued

| Author(s) (year) and  study no. [#]^a^ | Country,  period and  population | Data and  sample size | Intervention  description | Outcome(s), measure(s)  and exposure | Study design and method | Key results and  time horizon of effects^b^ | Summary^c^ |
| --- | --- | --- | --- | --- | --- | --- | --- |
|  |  |  |  |  |  |  |  |
| Born, Ovwigho and Cordero (2002)  [6] | USA  (Maryland)  1996-1997  Low-income families | Administrative data: Maryland Automated Benefits System (MABS), Automated Information Management System (AIMS), Automated Master File (AMF), Client Information System (CIS), Client Automated Resource and Eligibility System (CARES)  n = 2,665 families | See description provided for Acs, Phillips and Nelson (2005) [2]. Maryland adopts a ‘work first’ approach in which TANF clients who are not exempt from work activities are required to participate as soon as possible. | *Adult outcomes*:  Long-term benefits:  1. Re-entry into benefits  *Exposure*:  Imposition of full-family (100%) sanction | Non-experimental:  Descriptive statistics (no statistical test provided for differences across groups) | Compared to other reasons for leaving welfare (e.g. income above  limit/started work), sanctioned cases have the highest rate of benefit return within 3 months (32%), of which: 58.6% are < 30 days; and 41.4% are between 31-90 days.  *Time horizon*: short-term | 1. n/a |

Notes: ^a^ Study number and reference for each study are reported in Table A4; ^b^ Time horizon of effects is classified as: short-term (0-12 months); medium-term (13-24 months), long-term (25+ months)

^c^ Direction of effect by outcome measure number: 🡩 = significant increase (p<0.05); 🡫 = significant decrease (p<0.05); ○ = no significant change (p>0.05).

TABLE A4. Continued

| Author(s) (year) and  study no. [#]^a^ | Country,  period and  population | Data and  sample size | Intervention  description | Outcome(s), measure(s)  and exposure | Study design and method | Key results and  time horizon of effects^b^ | Summary^c^ |
| --- | --- | --- | --- | --- | --- | --- | --- |
| Busk (2016)  [7] | Finland  2003-2010  Unemployment Insurance (UI) and Labour Market Support (LMS) claimants | Administrative data:  Ministry of Employment and the Economy, Finnish Centre for Pensions  n = (up to) 920,137 spells;  486,651 individuals | Members of a UI fund qualify for UI payments, subject to sufficient employment history.  UI benefits:  Wage compensation rate: 90% (max);  Maximum duration: 500 business days.  LMS benefits:  LMS is flat-rate and means-tested and available to UI fund members with insufficient employment history or >500 days UI receipt. Duration is essentially unlimited though is approx. 50% of UI level. | *Adult outcomes*:  Employment:  1. Entry into employment  Benefits:  2. Entry into ALMP  Non-employment/ inactivity:  3. Entry into non-employment/inactivity  *Exposure*:  First imposition of a sanction (100%);  Duration: 60 days (66.4%), 150 days (29.9%), 90 days (2.9%), 30 days (0.8%). Sanctioned individuals may apply for other benefits such as basic social assistance (SA) / general housing allowance (HA). | Non-experimental:  Timing-of-events: mixed proportional hazards models | The effect of sanctions differs according to the benefits received. The imposition of a sanction increases the risk of entry into employment by 25% for UI claimants and 84% for LMS claimants. For LMS claimants, completed sanctions also increase the risk of entry into employment by 34%. For LMS claimants, the imposition of a sanction increases the risk of entry into an ALMP by 10.5% and by 4% for complete sanctions. The imposition of a sanction increases the risk of exit to outside the labour force by 82% for UI claimants and 58% for LMS claimants. For LMS claimants, completed sanctions also increase the risk of exit to outside the labour force by 27%.  *Time horizon*: long-term | 1. 🡩 (UI),  🡩 (LMS)  2. ○ (UI),  🡩 (LMS)  3. 🡩 (UI),  🡩 (LMS) |
| Chavkin, Romero and Wise (2000)  [8] | USA  1995-1998  Low-income families/lone parents | Linked  administrative-survey data:  various sources,  1995-1998  n = 50 states | See description provided for Acs, Phillips and Nelson (2005) [2]. | *Adult outcomes*:  Benefits:  1. % change in state benefit claims  *Exposure*: Imposition of a full-family sanction (100%) for initial non-compliance *versus* imposition of a benefit sanction for work non-compliance to Medicaid receipt (100%) (at state level) | Non-experimental:  Ordinary Least Squares (OLS) regression model | State-level sanctions policies are not shown to be associated with welfare enrolment.  *Time horizon*: medium-term | 1. ○ |

Notes: ^a^ Study number and reference for each study are reported in Table A4; ^b^ Time horizon of effects is classified as: short-term (0-12 months); medium-term (13-24 months), long-term (25+ months)

^c^ Direction of effect by outcome measure number: 🡩 = significant increase (p<0.05); 🡫 = significant decrease (p<0.05); ○ = no significant change (p>0.05).

TABLE A4. Continued

| Author(s) (year) and  study no. [#]^a^ | Country,  period and  population | Data and  sample size | Intervention  description | Outcome(s), measure(s)  and exposure | Study design and method | Key results and  time horizon of effects^b^ | Summary^c^ |
| --- | --- | --- | --- | --- | --- | --- | --- |
|  |  |  |  |  |  |  |  |
| Cherlin et al.  (2002)  [9] | USA  (Boston, MA, Chicago, IL, and San Antonio, TX)  1999  Low-income families | Survey data:  Children and welfare: a three-city study, 1999  n = 108 individuals | See description provided for Acs, Phillips and Nelson (2005) [2].  Sanctioning rates: substantial variation at the levels of benefit office and caseworker. | *Adult outcomes*:  Employment:  1. Entry into employment  *Exposure*:  Imposition of partial to full-family (100%) sanctions (vary by state). | Non-experimental:  Descriptive statistics | Of families that reported a partial or full loss of benefits (without complete restoration of these benefits), over a third (36%) got a job.  *Time horizon*: medium-term | 1. n/a |
| Diop-Christensen (2015)  [10] | Denmark  2006-2008  Long-term social assistance (SA) claimants | Administrative data:  Integrated Database for Labour Market Research (IDA) and Danish Register for Evaluation of Marginalization (DREAM) database  n = 5,564 spells;  5,564 individuals | In 2006, a new '300 rule' required married long-term SA recipients to work 300 hours in non-subsidised employment during a 2-year period in order to remain eligible for benefits. Study focuses on transition phase when 150 hours over a 12-month period was required. Local authorities had to notify potentially affected families by letter 6 months in advance. | *Adult outcomes*:  Job stability:  1. Entry into employment lasting ≤ 3 months  2. Entry into employment lasting > 3 months  Benefits:  3. Entry into other benefits  *Exposure*:  Warning letter (W) and first imposition of sanction (100%) (I), which lasts until compliance (requalify by working 150 hours, starting from date of sanction) | Non-experimental:  Multi-level discrete-time competing risks models | The imposition of a sanction (I) increases the transition rate by 4.4 times to short-term employment and by 1.3 times to longer-term employment. While receiving a warning letter (W) is not significantly associated with transitions to short-term employment, it significantly increases transitions to longer-term employment by 60%. The results also show that receiving a warning letter significantly increases the chances of moving to other benefits by 3.8 times for SA claimants.  *Time horizon*: short- to medium-term | 1. ○ (W)  🡩 (I)  2. 🡩 (W)  🡩 (I)  3. 🡩 (W)  ○ (I) |

Notes: ^a^ Study number and reference for each study are reported in Table A4; ^b^ Time horizon of effects is classified as: short-term (0-12 months); medium-term (13-24 months), long-term (25+ months)

^c^ Direction of effect by outcome measure number: 🡩 = significant increase (p<0.05); 🡫 = significant decrease (p<0.05); ○ = no significant change (p>0.05).

TABLE A4. Continued

| Author(s) (year) and  study no. [#]^a^ | Country,  period and  population | Data and  sample size | Intervention  description | Outcome(s), measure(s)  and exposure | Study design and method | Key results and  time horizon of effects^b^ | Summary^c^ |
| --- | --- | --- | --- | --- | --- | --- | --- |
|  |  |  |  |  |  |  |  |
| Farrell et al. (2008)  [11] | USA  2000-2005  Low-income families | Administrative data:  TANF Data Report  n = 389,178 families | See description provided for Acs, Phillips and Nelson (2005) [2]. | *Adult outcomes*:  Long-term benefits:  1. Probability of accumulating at least 60 months of benefit receipt  2. Number of months accumulated  *Exposure*:  Imposition of partial to full-family (100%) sanctions (varies by state – full-family can be gradual or immediate) (state-level indicator) | Non-experimental:  Probit and Ordinary Least Squares (OLS) regression models | Claimant families in states that impose immediate full-family sanctions are less likely to have accumulated 60 months on benefit and accumulated less months on benefit, relative to cases in states that impose partial or gradual full-family sanctions.  *Time horizon*: long-term | 1. 🡫  2. 🡫 |
| Graefe, Irving and DeJong (2006)  [12] | USA  1996-2003  Low-income families | Survey data: Survey of Income and Program Participation (SIPP), 1996 and 2001  n = 7,062 spells;  4,999 individuals | See description provided for Acs, Phillips and Nelson (2005) [2]. | *Adult outcomes*:  Employment  1. Entry into employment  (Transition from TANF to work without TANF)  Non-employment/ inactivity  2. Entry into non-employment/inactivity  (Transition from TANF to neither work nor TANF)  *Exposure*:  State-level indicator:  Stringent sanction policies (temporary or permanent ineligibility) versus lenient sanction policies (partial loss of benefits) | Non-experimental:  Discrete-time competing risks models: multinomial logistic regression models | Compared with partial sanction policies, stringent sanction policies are not shown to have a significant association with transitions to employment or economic inactivity.  *Time horizon*: long-term | 1. ○  2. ○ |

Notes: ^a^ Study number and reference for each study are reported in Table A4; ^b^ Time horizon of effects is classified as: short-term (0-12 months); medium-term (13-24 months), long-term (25+ months)

^c^ Direction of effect by outcome measure number: 🡩 = significant increase (p<0.05); 🡫 = significant decrease (p<0.05); ○ = no significant change (p>0.05).

TABLE A4. Continued

| Author(s) (year) and  study no. [#]^a^ | Country,  period and  population | Data and  sample size | Intervention  description | Outcome(s), measure(s)  and exposure | Study design and method | Key results and  time horizon of effects^b^ | Summary^c^ |
| --- | --- | --- | --- | --- | --- | --- | --- |
|  |  |  |  |  |  |  |  |
| Hillmann and Hohenleitner (2015)  [13] | Germany  2005-2007  Unemployment Benefit II (UB II) claimants. | Survey data: Labour Market and Social Security (PASS) survey, 2006-2007 and 2007-2008  n = 3,996 spells; 3,599 individuals | Since 2005 UB II merges Unemployment Assistance and Social Assistance (tax-based and means-tested); UB II claimants are required to register with the welfare agency and sign an ‘integration contract’.  Welfare agencies have discretionary powers for imposing sanctions; no formal warning when a non-compliance is detected.  UB II applies to households (‘need units’) with at least one person capable of working. Unemployed claimants typically have exceeded their maximum period of UI receipt or were not eligible for UI due to insufficient contributions  Study focuses on unemployed and low-income employed separately. | *Adult outcomes*:  Employment:  1. Entry into employment  Non-employment/ inactivity:  2. Entry into non-employment/inactivity  *Exposure*:  First imposition of a sanction affects base benefit: 10% (minor sanction), 30% (major sanction).  Duration: 90 days. Housing/accommodation costs and social security contributions unaffected. Harsher sanctions apply for young people. | Non-experimental:  Timing-of-events: mixed proportional hazards models | Sanctions increase the risk of entry into employment by 68-70% and into non-employment by 60-79%.  *Time horizon*: long-term | 1. 🡩  2. 🡩 |

Notes: ^a^ Study number and reference for each study are reported in Table A4; ^b^ Time horizon of effects is classified as: short-term (0-12 months); medium-term (13-24 months), long-term (25+ months)

^c^ Direction of effect by outcome measure number: 🡩 = significant increase (p<0.05); 🡫 = significant decrease (p<0.05); ○ = no significant change (p>0.05).

TABLE A4. Continued

| Author(s) (year) and  study no. [#]^a^ | Country,  period and  population | Data and  sample size | Intervention  description | Outcome(s), measure(s)  and exposure | Study design and method | Key results and  time horizon of effects^b^ | Summary^c^ |
| --- | --- | --- | --- | --- | --- | --- | --- |
|  |  |  |  |  |  |  |  |
| Hofferth, Stanhope and Harris (2002)  [14] | USA  1989-1996  Lone parents/  AFDC recipients | Survey data: Panel Study of Income Dynamics (PSID), 1989-1996  n = (up to) 18,207 person-months observations;  889 individuals | See description provided for Acs, Phillips and Nelson (2005) [2].  Prior to 1996, states had leeway to opt out of AFDC system requirements and to develop their own welfare policies, such as: work requirements, stringent child support enforcement provisions, and rules that increased cash assistance for married-couple families. | *Adult outcomes*:  Employment  1. Entry into employment  (Exit from benefits due to work)  Non-employment/ inactivity  2. Exit from benefits  *Exposure:*  State-level indicator: Imposition of partial to full-family (100%) sanctions (varies by state) | Non-experimental:  Discrete-time hazards models: logistic regression models with state fixed effects | State-level sanction policies were not significantly associated with welfare exits during this early period of the welfare reform.  *Time horizon*: long-term | 1. ○  2. ○ |
| Hofferth, Stanhope and Harris (2005)  [15] | USA  1989-1996  Lone parents/  AFDC recipients | Survey data: Panel Study of Income Dynamics (PSID), 1989-1996  n = 15,133 person-month observations; 742 individuals | See description provided for Acs, Phillips and Nelson (2005) [2].  Prior to 1996, states had leeway to opt out of AFDC system requirements and to develop their own welfare policies, eg: work requirements, child support enforcement, and rules that increased cash assistance for married-couple families. | *Adult outcomes*:  Long-term benefits:  1. Re-entry into benefits  *Exposure:*  State-level indicator: Imposition of partial to full-family (100%) sanctions (varies by state) | Non-experimental:  Discrete-time hazards models: logistic regression models with state fixed effects | State-level sanction policies are not shown to be significantly associated with benefit re-entry up to 96 months after AFDC exit.  *Time horizon*: long-term | 1. ○ |

Notes: ^a^ Study number and reference for each study are reported in Table A4; ^b^ Time horizon of effects is classified as: short-term (0-12 months); medium-term (13-24 months), long-term (25+ months)

^c^ Direction of effect by outcome measure number: 🡩 = significant increase (p<0.05); 🡫 = significant decrease (p<0.05); ○ = no significant change (p>0.05).

TABLE A4. Continued

| Author(s) (year) and  study no. [#]^a^ | Country,  period and  population | Data and  sample size | Intervention  description | Outcome(s), measure(s)  and exposure | Study design and method | Key results and  time horizon of effects^b^ | Summary^c^ |
| --- | --- | --- | --- | --- | --- | --- | --- |
|  |  |  |  |  |  |  |  |
| Hofmann (2012)  [16] | Germany (West)  2000-2005  Unemployment Insurance (UI) benefit claimants | Administrative data: Federal Employment Agency (FEA)  n = (up to) 12,418 individuals | UI benefits available for those who paid social contributions for at least 12 months within the 3 years prior to unemployment.  Wage compensation rate: 67% for unemployed with children, 60% if no children.  Maximum duration: 32 months. UI claimants are eligible for means-tested Unemployment Assistance (UA) once maximum duration reached. | *Adult outcomes*:  Job stability:  1. Entry into regular employment  Job quality:  2. Entry into other employment (subsidised employment and/or minor/short-term jobs)  Non-employment/ inactivity:  3. Entry into non-employment/inactivity  *Exposure:*  Imposition of 100% benefit sanction; duration: 84 days | Non-experimental:  Propensity Score Matching (PSM) | First, a sanction imposed in the first four months of individual UI benefit receipt increases the probability of subsequent regular employment for both women (W) and men (M). The positive effect on regular employment is persisting up to four years after the sanction, yet, for women only for the very early imposed sanctions. Second, these effects on regular employment are mainly driven by younger UI benefit recipients. Third, results suggest that older women respond to a sanction by taking up jobs of lower quality. Finally, the average number of months not being registered in the official work force is higher after a sanction for both women and men. These findings suggest that sanctions adversely affect the post-unemployment career of some sanctioned individuals.  *Time horizon*: long-term | 1. 🡩 (W)  ….🡩 (M)  2. 🡩 (W)  ….🡫 (M)  3. 🡩 (W)  ….🡩 (M) |

Notes: ^a^ Study number and reference for each study are reported in Table A4; ^b^ Time horizon of effects is classified as: short-term (0-12 months); medium-term (13-24 months), long-term (25+ months)

^c^ Direction of effect by outcome measure number: 🡩 = significant increase (p<0.05); 🡫 = significant decrease (p<0.05); ○ = no significant change (p>0.05).

TABLE A4. Continued

| Author(s) (year) and  study no. [#]^a^ | Country,  period and  population | Data and  sample size | Intervention  description | Outcome(s), measure(s)  and exposure | Study design and method | Key results and  time horizon of effects^b^ | Summary^c^ |
| --- | --- | --- | --- | --- | --- | --- | --- |
|  |  |  |  |  |  |  |  |
| Hohenleitner and Hillmann (2019a)  [17] | Germany  2008-2010  Unemployment Benefit II (UB II) claimants | Administrative data: Integrated Labour Market Biographies (SIAB) and German Federal Employment Agency (FEA)  n = 978,459 spells;  223,725 individuals | See description provided for Hillmann and Hohenleitner (2015) [13] | *Adult outcomse*:  Employment  1. Entry into employment  Non-employment/ inactivity  2. Exit from benefits  *Exposure:*  First imposition of a sanction affects base benefit: 10% (minor sanction), 30% (major sanction).  Duration: 90 days. Housing/accommodation costs and social security contributions unaffected. Harsher sanctions apply for young people. | Non-experimental:  Propensity Score Matching (PSM) | The effect of imposed sanctions is highly volatile over time and strongly dependent on the groups and subgroups that are analysed, their individual characteristics, regional differences, and the timing of the sanction.  In the short-term, there are positive effects on the probability of entering employment and larger positive effects on exiting benefits, which suggests increases in movements out of the labour force. The positive effects tend to be stronger in the short-term, and the observed negative effects tend to be stronger in the medium-term. In addition, the early positive effects are mainly driven by people with good labour market prospects and come at the cost of people with strongly detrimental sanction effects, even in the medium-term.  *Time horizon*: short- to medium-term | 1. 🡩  (short-term)  2. 🡩  (short-term) |

Notes: ^a^ Study number and reference for each study are reported in Table A4; ^b^ Time horizon of effects is classified as: short-term (0-12 months); medium-term (13-24 months), long-term (25+ months)

^c^ Direction of effect by outcome measure number: 🡩 = significant increase (p<0.05); 🡫 = significant decrease (p<0.05); ○ = no significant change (p>0.05).

TABLE A4. Continued

| Author(s) (year) and  study no. [#]^a^ | Country,  period and  population | Data and  sample size | Intervention  description | Outcome(s), measure(s)  and exposure | Study design and method | Key results and  time horizon of effects^b^ | Summary^c^ |
| --- | --- | --- | --- | --- | --- | --- | --- |
|  |  |  |  |  |  |  |  |
| Hohenleitner and Hillmann (2019b)  [18] | Germany  2008-2010  Unemployment Benefit II (UB II) claimants | Linked administrative data: Integrated Labour Market Biographies (SIAB), 2004-2010 and data from the German Federal Employment Agency (FEA)  n = n/a | See description provided for Hillmann and Hohenleitner (2015) [13] | *Adult outcomes*:  Job stability:  1. Cumulative duration in unsubsidised employment post UB II claim  Long-term benefits:  2. Cumulative duration in unemployment post UB II claim  Employment:  3. Cumulative duration in employment with supplementary UB II receipt  Earnings/income  4. Daily wage of first employment spell post UB II claim  5. Yearly income of first/second year post UB II claim  *Exposure:*  First imposition of a sanction affects base benefit: 10% (minor sanction), 30% (major sanction).  Duration: 90 days. Housing/accommodation costs and social security contributions unaffected. Harsher sanctions apply for young people. | Non-experimental:  Propensity Score Matching (PSM) | Sanctions are associated with highly significant and strongly negative effects on the quality of post-benefit employment, in both the short- and medium-term. For employment stability and income, there is a catch-up effect which is by far not strong enough to compensate the loss within two years. For employed welfare recipients the negative effects on job stability and income even exceed the effects for unemployed. Particularly striking are the remarkably strong and highly significant negative effects on indirectly affected unemployed household members.  *Time horizon*: short- to medium-term | 1. 🡫  2. 🡩  3. ○  4. 🡫  5. 🡫 |

Notes: ^a^ Study number and reference for each study are reported in Table A4; ^b^ Time horizon of effects is classified as: short-term (0-12 months); medium-term (13-24 months), long-term (25+ months)

^c^ Direction of effect by outcome measure number: 🡩 = significant increase (p<0.05); 🡫 = significant decrease (p<0.05); ○ = no significant change (p>0.05).

TABLE A4. Continued

| Author(s) (year) and  study no. [#]^a^ | Country,  period and  population | Data and  sample size | Intervention  description | Outcome(s), measure(s)  and exposure | Study design and method | Key results and  time horizon of effects^b^ | Summary^c^ |
| --- | --- | --- | --- | --- | --- | --- | --- |
|  |  |  |  |  |  |  |  |
| Irving (2008)  [19] | USA  1996-1999 and 2001-2003  Lone parents/ female TANF recipients | Linked admininistrative-survey data:  Survey of Income and Program Participation (SIPP), 1996-1999 and 2001-2003 panels;  State-level admin data:  Welfare Rules Database (WRD),  Local Area Unemployment Statistics (LAUS), Occupational Employment Statistics (OUS)  n = (up to) 5,561 spells;  4,487 TANF recipients | See description provided for Acs, Phillips and Nelson (2005) [2]. | *Adult outcomes*:  Employment:  1. Entry into employment  Non-employment/ inactivity:  2. Entry into non-employment  *Exposure*:  Imposition of partial or full benefit sanctions (ineligibility)  (at state level) | Non-experimental:  Multi-level discrete-time competing risks models: multinomial logistic regression models | Benefit sanctions increase the transition rates to non-employment by 22%, while there is no significant association with transition rates to employment. In the latter case no effect by areas of residence is observed.  *Time horizon*: short-term | 1. ○  2. 🡩 |

Notes: ^a^ Study number and reference for each study are reported in Table A4; ^b^ Time horizon of effects is classified as: short-term (0-12 months); medium-term (13-24 months), long-term (25+ months)

^c^ Direction of effect by outcome measure number: 🡩 = significant increase (p<0.05); 🡫 = significant decrease (p<0.05); ○ = no significant change (p>0.05).

TABLE A4. Continued

| Author(s) (year) and  study no. [#]^a^ | Country,  period and  population | Data and  sample size | Intervention  description | Outcome(s), measure(s)  and exposure | Study design and method | Key results and  time horizon of effects^b^ | Summary^c^ |
| --- | --- | --- | --- | --- | --- | --- | --- |
| Kim (2010)  [20] | USA  1996-2002  Female TANF recipients who participated in welfare-to-work programs | Linked administrative-survey data:  Survey of Program Dynamics (SPD), 1997-2001;  State-level admin data:  Welfare Rules Database (WRD), 1996-2002;  Local Area Unemployment Statistics (LAUS).  n = (up to) 4,020 spells;  251 mothers TANF recipients | The work-first approach, supported by sanctions, is intended to increase self-sufficiency through work and reduce dependency on social security benefits.  The work-first approach is implemented through two programmes: 1. Labor Force Attachment (LFA) programme which emphasizes short-term/less expensive job-related activities; 2. Human Capital Development (HCD) programme with longer-term, more expensive education and job training activities. | *Adult outcomes*:  Non-employment/ inactivity:  1. Exit from benefits  Long-term benefits:  2. Re-entry into benefits  *Exposure*:  Imposition of partial (for a specific time or until compliance) or full (100%) benefit sanctions (ineligibility for a specific time, until compliance or for life) (at state level) | Non-experimental:  Discrete-time hazard models: logistic regression models with random effects | Benefit sanctions are not significantly associated with either benefit exit and re-entry. Moreover, participation in a labour force attachment (LFA) programme was not associated with a higher likelihood of exiting benefits compared to participation in a Human Capital Development (HCD) programme or both types of welfare-to-work programmes. Benefit leavers who participated in both LFA and HDC programmes were more likely to return to benefits than those who participated in a LFA programme.  *Time horizon*: short-term | 1. ○  2. ○ |
| Koning (2015)  [21] | Netherlands  (Amsterdam)  2008-2012  Social Assistance (SA) recipients with debt problems | Administrative data (unspecified)  n = 29,855 spells;  23,769 individuals | Priority Care Debt Services (PCDS) is an intervention for Social Assistance (SA) recipients with debt problems to help restructuring personal debts, preventing new debt problems, and increasing direct incentives to resume work.  PCDS offered budgeting courses and assistance for debt restructuring and was meant to last up to one year. | *Adult outcomes*:  Employment:  1. Entry into employment  Non-employment/  inactivity:  2. Entry into non-employment  *Exposure*:  Imposition of a sanction: temporary benefit reduction or full benefit suspension | Non-experimental:  Timing-of-events: mixed proportional hazards model | The debt programme increased the transitions from SA to non-employment by 16%, while the association with transitions to employment was not significant. This suggests the presence of threat effects.  *Time horizon*: long-term | 1. ○  2. 🡩 |

Notes: ^a^ Study number and reference for each study are reported in Table A4; ^b^ Time horizon of effects is classified as: short-term (0-12 months); medium-term (13-24 months), long-term (25+ months)

^c^ Direction of effect by outcome measure number: 🡩 = significant increase (p<0.05); 🡫 = significant decrease (p<0.05); ○ = no significant change (p>0.05).

TABLE A4. Continued

| Author(s) (year) and  study no. [#]^a^ | Country,  period and  population | Data and  sample size | Intervention  description | Outcome(s), measure(s)  and exposure | Study design and method | Key results and  time horizon of effects^b^ | Summary^c^ |
| --- | --- | --- | --- | --- | --- | --- | --- |
| Lalive, van Ours and Zweimüller (2005)  [22] | Switzerland  (selected cantons)  1997-1999  Unemployment Insurance (UI) benefit claimants, aged 20-50 years | Administrative data: Swiss UI benefit register  n = 10,404 individuals | See description provided for Arni and Schiprowski (2015) [4]. | *Outcome*:  Non-employment/ inactivity:  1. Exit from benefits  *Exposure:*  Imposition of a 100% benefit sanction;  duration: typically 1-15 days (88%) (remainder 12% 16-60 days) | Non-experimental:  Timing-of-events: mixed proportional hazards model | Both sanction warnings and impositions have a positive effect on the exit rate out of unemployment. Sanction warnings increase the risk of exit from benefits by 25.2%, which increases again by 19.8% once the sanction has been imposed. Increasing the monitoring intensity also reduces the duration of unemployment of the non-sanctioned.  *Time horizon*: medium-term | 1. 🡩 (imposition, warning and threat) |
| Lee, Slack and Lewis (2004)  [23] | USA  (Illinois)  1999-2001  Female TANF recipients aged 15-64 years with a child aged < 18 years | Linked administrative-survey data:  Illinois Family Study (IFS), 1999/2000, 2001 and 2002;  Admin data from Illinois Department of Human Services and the Illinois Department of Employment Security  n = (up to) 1,123 individuals | See description provided for Acs, Phillips and Nelson (2005) [2]. | *Adult outcomes*:  Employment:  1. In formal employment  Non-employment/  inactivity:  2. Being off TANF  Earnings/income:  3. Earnings ($) from formal employment  *Exposure*:  Imposition (I) or threat (T) of sanctions. Gradual sanctions with 3 steps: (1) Partial-grant sanctions (50% reduction) until recipient cooperates or demonstrates good cause; (2) Partial-grant sanctions: benefits reduced by extra 50% until recipient cooperates or for 3 months; (3) Full-grant or full-family sanction for 3 months. | Non-experimental:  Ordinary Least Squares (OLS) and logistic regression models | The imposition (I) of a sanction is associated with a 56% reduction in the chances of being employed and a reduction in earnings of 1,320 ($) in the short-to-medium-term. The threat (T) of sanctions is not significantly associated with formal employment and benefit exit.  *Time horizon*: short- to medium-term | 1. 🡫 (I)  ○ (T)  2. ○ (I)  ○ (T)  3. 🡫 (I)  ○ (T) |

Notes: ^a^ Study number and reference for each study are reported in Table A4; ^b^ Time horizon of effects is classified as: short-term (0-12 months); medium-term (13-24 months), long-term (25+ months)

^c^ Direction of effect by outcome measure number: 🡩 = significant increase (p<0.05); 🡫 = significant decrease (p<0.05); ○ = no significant change (p>0.05).

TABLE A4. Continued

| Author(s) (year) and  study no. [#]^a^ | Country,  period and  population | Data and  sample size | Intervention  description | Outcome(s), measure(s)  and exposure | Study design and method | Key results and  time horizon of effects^b^ | Summary^c^ |
| --- | --- | --- | --- | --- | --- | --- | --- |
|  |  |  |  |  |  |  |  |
| Lee and Tomohara  (2007)  [24] | USA  1996-2000  Women aged 16-54 years/ TANF recipients | Survey data: Current Population Survey (CPS), 1996-2000;  State- or area-level aggregated published data  n = 185,682 individuals;  ~60,000 households | See description provided for Acs, Phillips and Nelson (2005) [2]. | *Adult outcomes*:  Benefits:  1. In receipt of benefits  *Exposure*:  ‘Activity sanctions’ which measures how strict states are in sanctioning those who do not satisfy job training programme requirements | Non-experimental:  Probit regression models with year and state fixed effects | Sanctions are associated with a 0.7% decrease in benefit receipt.  *Time horizon*: long-term | 1. 🡫 |
| Lee and Yoon  (2012)  [25] | USA  1999-2002  TANF leavers  < 65 years | Survey data: National Survey of America’s Families (NASF), 1999 and 2002  n = (up to) 2,045 individuals | See description provided for Acs, Phillips and Nelson (2005) [2]. | *Adult outcomes*:  Employment:  1. Currently employed  Earnings/income:  2. Family income as a % poverty line (1998-2001)  *Exposure*:  Imposition of partial or full benefit sanction. | Non-experimental:  Logistic and ordinal logistic regression models | Benefit sanctions decrease the odds of being in employment by 18% and decreases family income by 24% among welfare leavers.  *Time horizon*: short-term | 1. 🡫  2. 🡫 |

Notes: ^a^ Study number and reference for each study are reported in Table A4; ^b^ Time horizon of effects is classified as: short-term (0-12 months); medium-term (13-24 months), long-term (25+ months)

^c^ Direction of effect by outcome measure number: 🡩 = significant increase (p<0.05); 🡫 = significant decrease (p<0.05); ○ = no significant change (p>0.05).

TABLE A4. Continued

| Author(s) (year) and  study no. [#]^a^ | Country,  period and  population | Data and  sample size | Intervention  description | Outcome(s), measure(s)  and exposure | Study design and method | Key results and  time horizon of effects^b^ | Summary^c^ |
| --- | --- | --- | --- | --- | --- | --- | --- |
|  |  |  |  |  |  |  |  |
| Lindhorst and Mancoske (2006)  [26] | USA  (Louisiana)  1998-2001  Female TANF leavers | Survey data: Panel Study of Welfare Recipients, 1998-2001  n = (up to) 348 female TANF leavers | See description provided for Acs, Phillips and Nelson (2005) [2]. | *Adult outcomes*:  Employment:  1. % working full-time or part-time (20+ hrs/week)  Benefits:  2. % receiving Food Stamps  Earnings/income:  3. Total monthly financial resources (employment, TANF payments, Food Stamps, child support, other financial resources (e.g. Supplemental Security Income, Social Security Disability payments))  *Exposure*:  Imposition of partial or full-family (100%) benefit sanctions | Non-experimental:  Descriptive analysis: Analysis of Variance (ANOVA) | There were no significant differences between those sanction and other welfare categories in terms of percentage of those who were employed, receiving Food Stamps, and their monthly financial resources at least one year from initial observation.  *Time horizon*: short-/medium-term | 1. ○  2. ○  3. ○ |
| Lindhorst, Mancoske, and Kemp (2000)  [27] | USA  (Southern metropolitan region)  1998  Low-income families/lone parents | Survey data: authors’ telephone survey, 1998  n = 347 individuals | See description provided for Acs, Phillips and Nelson (2005) [2]. | *Adult outcomes*:  Employment:  1. Employment status  Earnings/income:  2. Total monthly income  *Exposure*:  Imposition of full-family (100%) sanctions | Non-experimental:  Descriptive analysis | When compared with individuals who left benefits voluntarily, sanctioned individuals were less likely to be working and had a lower level of reported income.  *Time horizon*: medium-term | 1. 🡫  2. 🡫 |

Notes: ^a^ Study number and reference for each study are reported in Table A4; ^b^ Time horizon of effects is classified as: short-term (0-12 months); medium-term (13-24 months), long-term (25+ months)

^c^ Direction of effect by outcome measure number: 🡩 = significant increase (p<0.05); 🡫 = significant decrease (p<0.05); ○ = no significant change (p>0.05).

TABLE A4. Continued

| Author(s) (year) and  study no. [#]^a^ | Country,  period and  population | Data and  sample size | Intervention  description | Outcome(s), measure(s)  and exposure | Study design and method | Key results and  time horizon of effects^b^ | Summary^c^ |
| --- | --- | --- | --- | --- | --- | --- | --- |
| Lissenburgh (2004)  [28] | UK  (Scotland)  1998-2001  Unemployment benefit claimants, aged 18-24 years | Linked administrative-survey data:  New Deal Evaluation Database (NDED), Joint Unemployment and Vacancies Operating System (JUVOS) and two-stage national survey conducted by Policy Studies Institute (PSI) and BMRB social research  n = (up to) 695 individuals (survey);  10,416 individuals (admin data) | The New Deal for Young People (NDYP) was introduced in 1998 to help young adults aged 18-24 years to enter the labour market by providing counselling and training. Participation is compulsory and failure to participate results in benefit sanctions.  After the first interview, participants entered a Gateway period where they receive intensive counselling about job search and job opportunities.  After 4 months, participants who are still on the programme may enter the next phase (lasting 6+ months; treatment group) which includes the following options: subsidised employment, full-time education/training; work for voluntary sector or environment task force.  Control group is formed by those who stayed for an extended period on Gateway programme and did not enter the above options. | *Adult outcomes*:  Non-employment/ inactivity:  1. % time off Jobseeker’s Allowance (JSA) benefits  2. Being off JSA  *Exposure*:  Take-up effect of participating into the New Deal for Young people (NDYD) programme implying a threat of sanctions. | Non-experimental:  Propensity Score Matching (PSM) | Participating into the New Deal for Young people (NDYD) programme increased the likelihood of benefit exit (6-10 percentage points in the treatment group versus 6 percentage points in the control group) and increased the percentage of time spent off JSA (9-14 percentage points in the treatment group versus 8 percentage points in the control group).  *Time horizon*: short-term | 1. 🡩  2. 🡩 |

Notes: ^a^ Study number and reference for each study are reported in Table A4; ^b^ Time horizon of effects is classified as: short-term (0-12 months); medium-term (13-24 months), long-term (25+ months)

^c^ Direction of effect by outcome measure number: 🡩 = significant increase (p<0.05); 🡫 = significant decrease (p<0.05); ○ = no significant change (p>0.05).

TABLE A4. Continued

| Author(s) (year) and  study no. [#]^a^ | Country,  period and  population | Data and  sample size | Intervention  description | Outcome(s), measure(s)  and exposure | Study design and method | Key results and  time horizon of effects^b^ | Summary^c^ |
| --- | --- | --- | --- | --- | --- | --- | --- |
| Moffitt (2000)  [29] | USA  (Boston, MA, Chicago, IL, and San Antonio, TX)  1996-1999  TANF leavers living in low- and moderate-income neighbourhoods | Survey data:  Three-City Study, 1999  n = 339 TANF leavers | See description provided for Acs, Phillips and Nelson (2005) [2].  Note that around 2/3 of the sample of TANF leavers receive other benefits (e.g. Medicaid, subsidised housing, school meals) while more than 1/3 receive food stamps. | *Adult outcomes*:  Employment:  1. % months employed  Non-employment/ inactivity:  2. % never employed  Earnings/income:  3. Average conditional median monthly earnings ($)  4. Welfare income from Child Support and Food Stamp ($)  *Exposure*:  Imposition of full-family (100%) benefit sanction | Non-experimental:  Descriptive analysis | Among TANF leavers, those who were sanctioned had lower rates of time in employment (19 percentage points) and higher rates of non-employment (10 percentage points) two years after interview date. On average, sanctioned TANF leavers also had lower median earnings and income from other welfare benefits ($805 and $97, respectively) compared to non-sanctioned TANF leavers ($919 and $121).  *Time horizon*: medium-term | 1. 🡫  2. 🡩  3. 🡫  4. 🡫 |
| Moore, Wood and Rangarajan (2012)  [30] | USA  (New Jersey)  1997-2003  TANF recipients | Linked administrative-survey data:  Work First New Jersey program administrative records data; client surveys conducted by Mathematical Policy Research, 1999-2003  n = 1,441 individuals | See description provided for Acs, Phillips and Nelson (2005) [2].  Work First is New Jersey’s employment programme. Recipients are required to participate in work activities. Work requirement is enforced through sanctions, benefit reductions for non-compliance which gradually increase toward full-family (100%) sanctions. New Jersey operates a 5-year time limit policy. In general New Jersey’s welfare policies are moderate. | *Adult outcomes*:  Non-employment/ inactivity:  1. Not employed or not receiving welfare benefits, not living with an employed partner, not incarcerated; receiving other means-tested benefits (e.g. Supplemental Nutrition Assistant Program (SNAP) or Food Stamps).  *Exposure*:  Imposition of full-family (100%) benefit sanctions | Non-experimental:  Discrete-time hazard models: logistic regression models | The imposition of a full sanction is significantly associated with a higher probability of transitioning into a disconnected status by 17% among female welfare recipients.  *Time horizon*: long-term | 1. 🡩 |

Notes: ^a^ Study number and reference for each study are reported in Table A4; ^b^ Time horizon of effects is classified as: short-term (0-12 months); medium-term (13-24 months), long-term (25+ months)

^c^ Direction of effect by outcome measure number: 🡩 = significant increase (p<0.05); 🡫 = significant decrease (p<0.05); ○ = no significant change (p>0.05).

TABLE A4. Continued

| Author(s) (year) and  study no. [#]^a^ | Country,  period and  population | Data and  sample size | Intervention  description | Outcome(s), measure(s)  and exposure | Study design and method | Key results and  time horizon of effects^b^ | Summary^c^ |
| --- | --- | --- | --- | --- | --- | --- | --- |
|  |  |  |  |  |  |  |  |
| Müller and Steiner (2008)  [31] | Germany  2000-2005  Unemployment Insurance (UI) and Unemployment Assistance (UA) benefits claimants | Administrative data: German Federal Employment Agency (FEA)  n = 314,283 individuals | The Federal Employment Agency (FEA), as part of the Hartz reforms, issued an internal decree calling for stricter activation and monitoring of the unemployed with a more frequent imposition of sanctions starting in the second quarter of 2003. | *Adult outcomes*:  Employment:  1. Entry into employment  *Exposure:*  Imposition of first sanction: 100%; duration: 84 days, then 21 days (following 2003 reform). Sanctioned individuals can still claim social assistance benefits for the sanction period and can still receive job-search assistance by the FEA. | Non-experimental:  Discrete-time hazard models with Propensity Score Matching (PSM) | Benefit sanctions are associated with positive employment effects. These exist in both the short- and long-term and are robust for men and women in East and West Germany. The effects diminish with the elapsed unemployment duration until a sanction is imposed. The limited use of benefit sanctions can thus be an effective activation tool if they take place not too late in an individual’s unemployment spell.  *Time horizon*: long-term | 1. 🡩 |
| Oggins and Fleming (2001)  [32] | USA  (New York state)  1997-1999  Low-income families | Survey conducted by authors, 1997 and 1999  n = 118 individuals | See description provided for Acs, Phillips and Nelson (2005) [2]. Sanction rates: There is an incentive for states to sanction claimants in order to get them off rolls, as this improves the work participation formula to maintain TANF block grant. | *Adult outcomes*:  Employment  1. Wages (income source)  Benefits  2. Benefits (income source)  Non-employment/ inactivity  3. Not in employment nor benefits (income source)  *Exposure*:  Imposition of 100% benefit sanctions; average duration: 106 days | Non-experimental:  Logistic regression | Those who had been sanctioned were more likely to report having neither work nor benefits and less likely than others to report receiving wages.  *Time horizon*: short- to medium-term | 1. 🡫  2. ○  3. 🡩 |

Notes: ^a^ Study number and reference for each study are reported in Table A4; ^b^ Time horizon of effects is classified as: short-term (0-12 months); medium-term (13-24 months), long-term (25+ months)

^c^ Direction of effect by outcome measure number: 🡩 = significant increase (p<0.05); 🡫 = significant decrease (p<0.05); ○ = no significant change (p>0.05).

TABLE A4. Continued

| Author(s) (year) and  study no. [#]^a^ | Country,  period and  population | Data and  sample size | Intervention  description | Outcome(s), measure(s)  and exposure | Study design and method | Key results and  time horizon of effects^b^ | Summary^c^ |
| --- | --- | --- | --- | --- | --- | --- | --- |
|  |  |  |  |  |  |  |  |
| Ovwigho et al. (2011)  [33] | USA  (Maryland)  1998-2006  TANF leavers | Linked administrative data: Maryland’s Longitudinal Study of Welfare Leavers, 1998-2006 (Client Automated Resources and Eligibility System (CARES); Maryland Automated Benefits System (MABS))  n = 3,555 individuals | See description provided for Acs, Phillips and Nelson (2005) [2].  Note that many of those persistently disconnected (not in employment nor in TANF during the follow-up period) are to a large extent in receipt of Supplemental Security Income (SSI), Child Support, additional income, moved out of state and to a less extent are in receipt of Unemployment benefits (UI). | *Adult outcomes*:  Long-term non-employment/inactivity:  1. Persistently not in employment nor benefits  *Exposure*:  Imposition of full-family (100%) benefit sanctions | Non-experimental:  Descriptive analysis | There are no statistically significant differences in terms of having a case closed due to work-related sanctions, between those who are persistently disconnected from the welfare system (not in employment/benefits) during the five years after leaving welfare.  *Time horizon*: long-term | 1. ○ |

Notes: ^a^ Study number and reference for each study are reported in Table A4; ^b^ Time horizon of effects is classified as: short-term (0-12 months); medium-term (13-24 months), long-term (25+ months)

^c^ Direction of effect by outcome measure number: 🡩 = significant increase (p<0.05); 🡫 = significant decrease (p<0.05); ○ = no significant change (p>0.05).

TABLE A4. Continued

| Author(s) (year) and  study no. [#]^a^ | Country,  period and  population | Data and  sample size | Intervention  description | Outcome(s), measure (s)  and exposure | Study design and method | Key results and  time horizon of effects^b^ | Summary^c^ |
| --- | --- | --- | --- | --- | --- | --- | --- |
|  |  |  |  |  |  |  |  |
| Peck (2007)  [34] | USA  (Atlanta, GA, Grand Rapids, MI, and Riverside, CA)  1991-1996  Low-income families without young children (aged 1-3 years) expected to participate in Job Opportunities and Basic Skills (JOBS) mandatory programme for 20+ hours/week | Linked administrative-survey data:  National Evaluation of Welfare-to-Work Strategies (NEWWS), 1991-1996  n =4,699 individuals | See description provided for Acs, Phillips and Nelson (2005) [2].  The implementation of sanction policies varies across sites and interacts with overall program design and discretion of frontline workers to carry out the sanction process. | *Adult outcomes*:  Employment:  1. Whether employed (%)  Benefits:  2. Cash assistance ($)  Earnings/income:  3. Earnings ($)  4. Income ($)  5. Net income ($)  *Exposure*:  High propensity of being sanctioned (treatment group) vs low propensity of being sanctioned (control group): reduction of cash assistance grants by the amount attributed to family’s adult (15%-20% of the total grant, depending on site and household characteristics). Duration: until compliance or up to 6 months. | Non-experimental:  Propensity Score Matching (PSM) | During the first three years follow-up period, having a higher sanction risk is associated with higher employment rates and higher earnings levels, but less cash assistance than those with lower sanction risk. As a result, sanctioned recipients have about the same (net) incomes as non-sanctioned recipients.  *Time horizon*: long-term | 1. 🡩  2. 🡫  3. 🡩  4. ○  5. ○ |

Notes: ^a^ Study number and reference for each study are reported in Table A4; ^b^ Time horizon of effects is classified as: short-term (0-12 months); medium-term (13-24 months), long-term (25+ months)

^c^ Direction of effect by outcome measure number: 🡩 = significant increase (p<0.05); 🡫 = significant decrease (p<0.05); ○ = no significant change (p>0.05).

TABLE A4. Continued

| Author(s) (year) and  study no. [#]^a^ | Country,  period and  population | Data and  sample size | Intervention  description | Outcome(s), measure(s)  and exposure | Study design and method | Key results and  time horizon of effects^b^ | Summary^c^ |
| --- | --- | --- | --- | --- | --- | --- | --- |
|  |  |  |  |  |  |  |  |
| Pingle (2005)  [35] | USA  1997-2000  Lone parents aged 18-30 years with children aged <5 years | Linked survey-admin data:  Current Population Survey (CPS) March Annual Demographic Supplement, 1997-2000;  Welfare Rules Database  n = 4,643 individuals | See description provided for Acs, Phillips and Nelson (2005) [2].  Before 1996, as part of AFDC, states were allowed to pay lower welfare benefits to single mothers who lived with their parents (shelter allowance); this was applied to women who lived with others (in general) or received public housing or any form of housing assistance. After 1996, many states retained these benefit reductions or similar policies. | *Adult outcomes*:  Employment:  1.Whether employed and living/not living with parents  Non-employment/ inactivity  2. Whether not employed and living with parents  *Exposure*:  Imposition of a severe initial sanction versus permanent sanction (state-level indicator) | Non-experimental:  Multi-level discrete-time competing risks models multinomial logistic regression models with random effects | Both the severity of the initial sanction and the imposition of permanent sanctions are not significantly associated the employment status of single mothers regardless of their living arrangements.  *Time horizon*: long-term | 1. ○  2. ○ |

Notes: ^a^ Study number and reference for each study are reported in Table A4; ^b^ Time horizon of effects is classified as: short-term (0-12 months); medium-term (13-24 months), long-term (25+ months)

^c^ Direction of effect by outcome measure number: 🡩 = significant increase (p<0.05); 🡫 = significant decrease (p<0.05); ○ = no significant change (p>0.05).

TABLE A4. Continued

| Author(s) (year) and  study no. [#]^a^ | Country,  period and  population | Data and  sample size | Intervention  description | Outcome(s), measure(s)  and exposure | Study design and method | Key results and  time horizon of effects^b^ | Summary^c^ |
| --- | --- | --- | --- | --- | --- | --- | --- |
|  |  |  |  |  |  |  |  |
| Qureshi (2013)  [36] | Denmark  2007-2008  Unemployment Assistance (UA) benefit claimants | Administrative data: Danish Register for Evaluation of Marginalization (DREAM), 2007-2008; KMD data set on sanctions records, COR data set with information on yearly income, 2006; MIA register containing records of workers with some regular job income; CPR-Register with information on number of children <18 years  n = 52,023 spells; 42,738 individuals | Unemployment Assistance (UA) is available to those who do not subscribe to an Unemployment Insurance (UI) fund, provided they can continually demonstrate workplace-availability. Requirements depend on whether the person faces barriers other than unemployment, such as alcoholism, chronic illness or poor language skills. | *Adult outcomes*:  Employment:  1. Entry into employment  *Exposure:*  First imposition of a sanction of 33% for 21 days (refuse work/ALMP) or 100% (missing a meeting – open-ended until contact re-established) | Non-experimental:  Timing-of-events: mixed proportional hazards models | The imposition of a sanction increases the risk of entry into employment by 52.8%.  *Time horizon*: medium-term | 1. 🡩 |

Notes: ^a^ Study number and reference for each study are reported in Table A4; ^b^ Time horizon of effects is classified as: short-term (0-12 months); medium-term (13-24 months), long-term (25+ months)

^c^ Direction of effect by outcome measure number: 🡩 = significant increase (p<0.05); 🡫 = significant decrease (p<0.05); ○ = no significant change (p>0.05).

TABLE A4. Continued

| Author(s) (year) and  study no. [#]^a^ | Country,  period and  population | Data and  sample size | Intervention  description | Outcome(s), measure(s)  and exposure | Study design and method | Key results and  time horizon of effects^b^ | Summary^c^ |
| --- | --- | --- | --- | --- | --- | --- | --- |
|  |  |  |  |  |  |  |  |
| Reeves (2017)  [37] | UK  (Great Britain)  2009-2014  Unemployment Insurance (UI) benefit claimants, receiving Job Seekers’ Allowance (JSA) and with long-term physical and mental disability, | Linked administrative-survey data (area-level):  various sources, 2009-2014  n = 364 local authorities;  2,067 local authority/ financial years observations | The reform of the UK Job Seekers’ Allowance (JSA) regime of 2012 was accompanied by a tightening of job-search requirements and an extension of both scope and duration of sanctions to ensure behavioural compliance. | *Adult outcomes*:  Employment:  1. % of employed people with a disability  Non-employment  inactivity:  2. % of economically inactive people with a disability  *Exposure*:  Number of disabled people who are sanctioned (% of all claimants within a local authority) | Non-experimental:  Fixed effects regression models | Sanction rates among those with a disability are significantly associated with an increase in the disability rate by 31% among those economically inactive. No significant association is found among those in employment.  *Time horizon*: long-term | 1. ○  2. 🡩 |

Notes: ^a^ Study number and reference for each study are reported in Table A4; ^b^ Time horizon of effects is classified as: short-term (0-12 months); medium-term (13-24 months), long-term (25+ months)

^c^ Direction of effect by outcome measure number: 🡩 = significant increase (p<0.05); 🡫 = significant decrease (p<0.05); ○ = no significant change (p>0.05).

TABLE A4. Continued

| Author(s) (year) and  study no. [#]^a^ | Country,  period and  population | Data and  sample size | Intervention  description | Outcome(s), measure(s)  and exposure | Study design and method | Key results and  time horizon of effects^b^ | Summary^c^ |
| --- | --- | --- | --- | --- | --- | --- | --- |
|  |  |  |  |  |  |  |  |
| Røed and Skogstrøm (2014)  [38] | Norway  1999-2007  New Unemployment Insurance (UI) benefit claimants (registered as unemployed job seekers with no prior registration in the past 6 months) | Norwegian administrative data, 1999-2007  n = 157,124 spells;  141,884 individuals | Unemployment Insurance (UI) benefit claimants are required to be in paid employment just before the unemployment spell; to have lost the job involuntarily; to have labour earnings of min. 120,000 NOK (15,000 Euros) last calendar year or 240,000 NOK over last 3 calendar years.  Wage compensation rate: 62.4% up to annual threshold of 480,000NOK  Max. duration: 3 years until December 2002; 2 years for new claimants from January 2003. | *Adult outcomes*:  Job stability:  1. Entry into regular employment for 3+ months  Earnings/income:  2. Total registered earnings during the first year after entry into regular employment  *Exposure:*  Imposition of ongoing/completed sanctions (temporary loss of UI benefits) for non-compliance with job-search requirement (e.g. fail to attend a meeting with caseworker, reject suitable job offers, refuse to participate in ALMPs); Duration of sanctions: typically 8 weeks. | Non-experimental:  Timing-of-events: mixed proportional hazards models | The imposition of an ongoing sanction increases the risk of entry into regular employment by 174%; the positive effect persists after the sanction is completed. Ongoing sanctions decreases the earnings from regular employment during the first year by 2%.  *Time horizon*: medium-term | 1. 🡩  2. 🡫 |

Notes: ^a^ Study number and reference for each study are reported in Table A4; ^b^ Time horizon of effects is classified as: short-term (0-12 months); medium-term (13-24 months), long-term (25+ months)

^c^ Direction of effect by outcome measure number: 🡩 = significant increase (p<0.05); 🡫 = significant decrease (p<0.05); ○ = no significant change (p>0.05).

TABLE A4. Continued

| Author(s) (year) and  study no. [#]^a^ | Country,  period and  population | Data and  sample size | Intervention  description | Outcome(s), measure(s)  and exposure | Study design and method | Key results and  time horizon of effects^b^ | Summary^c^ |
| --- | --- | --- | --- | --- | --- | --- | --- |
| Schneider (2008)  [39] | Germany  2004-2006  Unemployment Benefit II (UB II) benefit claimants | Survey data:  Life Situation and Social Security, 2005  n = (up to) 12,822 individuals | See description provided for Hillmann and Hohenleitner (2015) [13].  Base UI benefit:  345€ (Germany, West), 331€ (Germany, East). The amount is igher for previous UI claimants due to payment of a bonus. | *Adult outcomes*:  Employment:  1. Entry into employment  Earnings/income:  2. Net hourly reservation wage  *Exposure:*  First imposition of a benefit sanction of 30% or 10%; duration: 90 days. | Non-experimental:  Propensity Score Matching (PSM) | The main result is that there was no significant effect of sanctions on the reservation wages of sanctioned UB II recipients. A side result is that sanctioned UB II recipients were not more likely to be employed at the time of their interview either.  *Time horizon*: medium-term | 1. ○  2. ○ |
| Schram, Fording and Soss (2008)  [40] | USA  (Florida)  2001-2002  Low-income families | Administrative data:  Florida's Welfare Transition (WT) programme  n = n/a | See description provided for Acs, Phillips and Nelson (2005) [2]. Sanction rates: Florida makes significant use of sanctions to enforce participation in its WT programme, and as a result has repeatedly received a ‘high performance’ federal bonus. | *Adult outcomes*:  Earnings/income  1. Earnings 30+ hours/  week at minimum wage  *Exposure:*  Imposition of full-family (100%) sanctions | Non-experimental:  Logistic regression models | Upon exiting TANF, the earnings gap between sanctioned and non-sanctioned clients increases, suggesting that TANF clients who exited due to sanction were significantly less likely to earn an income level equivalent to full-time, minimum wage employment.  *Time horizon*: short-term | 1. 🡫 |
| Snarr  (2013)  [41] | USA  1990-2005  Low-income families | State-level panel data, various sources,  1990-2005  n = 800 state-year observations | See description provided for Acs, Phillips and Nelson (2005) [2]. | *Adult outcomes*:  Job quality:  1. Employment population ratio of women living under 150% of poverty  Benefits:  2. Average monthly welfare caseload relative to their corresponding peaks  *Exposure*:  Imposition of full-family (100%) sanctions  (at state-level) | Non-experimental:  Three-Stage Least Squares (3SLS) regression models | Full-family sanctions are associated with a significant decrease in welfare caseloads by 3.3%; there is no significant association between the imposition of full-family sanctions and the employment population ratio of women living in poverty.  *Time horizon*: long-term | 1. ○  2. 🡫 |

Notes: ^a^ Study number and reference for each study are reported in Table A4; ^b^ Time horizon of effects is classified as: short-term (0-12 months); medium-term (13-24 months), long-term (25+ months)

^c^ Direction of effect by outcome measure number: 🡩 = significant increase (p<0.05); 🡫 = significant decrease (p<0.05); ○ = no significant change (p>0.05).

TABLE A4. Continued

| Author(s) (year) and  study no. [#]^a^ | Country,  period and  population | Data and  sample size | Intervention  description | Outcome(s), measure(s)  and exposure | Study design and method | Key results and  time horizon of effects^b^ | Summary^c^ |
| --- | --- | --- | --- | --- | --- | --- | --- |
|  |  |  |  |  |  |  |  |
| Svarer (2011)  [42] | Denmark  2003-2005  Unemployment Insurance (UI) benefits claimants | Administrative data:  Danish Register for Evaluation of Marginalization (DREAM) and AMANDA register of sanctions  n = (up to) 219,348 spells;  164,962 individuals | See description provided for Ahmad, Svarer and Naveed (2019) [3]. | *Adult outcomes*:  Non-employment/ inactivity:  1.Exit from benefits  *Exposure*:  First imposition of a benefit sanction (assumed 100%); aggregate sanction duration (typically either 2-3 days or 3 weeks) | Non-experimental:  Timing-of-events: mixed proportional hazards models | The imposition of a sanction increases the exit rate from unemployment by more than 100% for both women and men; the effect of sanctions decreases over time (after 3 months) and becomes not significant.  *Time horizon*: short-term | 1.🡩 |
| Swenson, White and Murdock (2002)  [43] | USA  (Texas)  1997-1999  Low-income families/lone parents | Administrative data from the Center for Demographic and Socioeconomic Research and Education, Texas Department of Human Services  n = 374,604 individuals | See description provided for Acs, Phillips and Nelson (2005) [2].  In Texas, sanctions were applied for failure to meet behavioural as well as work requirements, but were never more than the adult portion of TANF payments. Many rural counties were exempted from work requirements, but still sanctioned for failure to meet behavioural requirements. The sanctioning rate is similar in rural and urban areas, with a sanctioning gap of 10% throughout the study period. | *Adult outcomes*:  Non-employment/ inactivity:  1. Exit from benefits  *Exposure*:  Imposition of benefit sanctions, ranging from $25 to adult portion of TANF benefit | Non-experimental:  Cox proportional hazards models | Receiving a sanction increases the likelihood of exiting from welfare by 22%, with the likelihood being slightly higher (23%) in non-metropolitan counties.  *Time horizon*: medium-term | 1. 🡩 |

Notes: ^a^ Study number and reference for each study are reported in Table A4; ^b^ Time horizon of effects is classified as: short-term (0-12 months); medium-term (13-24 months), long-term (25+ months)

^c^ Direction of effect by outcome measure number: 🡩 = significant increase (p<0.05); 🡫 = significant decrease (p<0.05); ○ = no significant change (p>0.05).

TABLE A4. Continued

| Author(s) (year) and  study no. [#]^a^ | Country,  period and  population | Data and  sample size | Intervention  description | Outcome(s), measure(s)  and exposure | Study design and method | Key results and  time horizon of effects^b^ | Summary^c^ |
| --- | --- | --- | --- | --- | --- | --- | --- |
|  |  |  |  |  |  |  |  |
| van den Berg and Vikström (2014)  [44] | Sweden  1999-2003  Unemployment Insurance (UI) benefits claimants | Linked administrative-survey data:  four population register datasets (employment offices, UI fund) and a large-scale annual employer survey (wages and hours worked)  n = (up to) 35,055 spells; 16,941 individuals | UI claimants must have been a member of a UI fund (1 year) and employed for 6 months in previous year.  Wage compensation rate: 80%;  Maximum duration: 300 days.  Focus of sanctions regime is on job acceptance monitoring rather than job-search. | *Adult outcomes*:  Employment:  1. Entry into employment  Job quality:  2. Entry into full-time employment  3. Monthly FTE wage  4. Occupational level  *Exposure:*  Pre-February 2001: imposition of 100% benefit sanction for 60 days (32% of sample).  Subsequent benefit sanctions: 25% for 40 days (68% of sample). | Non-experimental:  Timing-of-events: mixed proportional hazards models | Sanctions increase the risk of entry into employment by 23%. However, after a sanction, the accepted wage rate is lower (by 4%) and individuals move more often to a part-time job and a lower occupational level, incurring human capital losses. The risk of entry into full-time employment decreases by 15%. A sanction causes unemployed individuals to accept employment within an occupation that, on average, requires 0.04 to 0.05 fewer years of schooling. Post-unemployment outcomes are also affected in the long run.  *Time horizon*: long-term | 1. 🡩  2. 🡫  3. 🡫  4. 🡫 |

Notes: ^a^ Study number and reference for each study are reported in Table A4; ^b^ Time horizon of effects is classified as: short-term (0-12 months); medium-term (13-24 months), long-term (25+ months)

^c^ Direction of effect by outcome measure number: 🡩 = significant increase (p<0.05); 🡫 = significant decrease (p<0.05); ○ = no significant change (p>0.05).

TABLE A4. Continued

| Author(s) (year) and  study no. [#]^a^ | Country,  period and  population | Data and  sample size | Intervention  description | Outcome(s), measure(s)  and exposure | Study design and method | Key results and  time horizon of effects^b^ | Summary^c^ |
| --- | --- | --- | --- | --- | --- | --- | --- |
|  |  |  |  |  |  |  |  |
| van den Berg, Hofmann and Uhlendorff (2019)  [45] | Germany (West)  2000-2002  Unemployment Insurance (UI) and Unemployment Assistance (UA) male benefit claimants aged 24-58 years | Administrative data:  Integrated Employment Biographies (IEB), benefit recipient history (LeH), participation-in-measures history (MTH) and applicants pool database (BewA)  n = 128,377 individuals | For UI benefits, see description provided for Hofman (2012) [16].  A vacancy referral is a letter of notification of a job opening for which the benefit claimant is required to apply as soon as possible (usually within 2 weeks). Sanctions are applied for not following up a vacancy referral for a job which is deemed suitable (commuting time < 2.5 hours and wage not below 70%-80% of previous wage).  UI benefits continue to be paid during a sickness period of up to 6 weeks, after which a unemployed person is required to apply to health insurance agency for coverage. During sickness periods, work-related requirements for benefit claiming (including vacancy referrals) are not applied. | *Adult outcomes*:  Employment:  1. Entry into employment  Job stability:  2. Exit from employment  Earnings/income:  2. Log(wage)  *Exposure:*  Longer sanction spell (3+ months) versus shorter sanction spell (<3 months) following imposition of full (100%) sanctions of 12 weeks (maximum duration), usually applied for not applying for a suitable job notified through a vacancy referral. | Non-experimental:  Timing-of-events: discrete-time hazard models (logistic regression models with random effects) | Shorter sanction spells increase transitions to employment by 40% but also increase exits from employment by 14%, while no effects are found for longer sanction spells. Initial wages following a shorter spell of sanction are 15% lower than wages of individuals who were not sanctioned. The effect is smaller  (-4.5%) for those with shorter sanction spells.  *Time horizon*: medium-term | 1. 🡩 (<3 mths)  ○ (3+ mths)  2. 🡩 (<3 mths)  ○ (3+ mths)  3. 🡫 (<3 mths)  🡫 (3+ mths) |

Notes: ^a^ Study number and reference for each study are reported in Table A4; ^b^ Time horizon of effects is classified as: short-term (0-12 months); medium-term (13-24 months), long-term (25+ months)

^c^ Direction of effect by outcome measure number: 🡩 = significant increase (p<0.05); 🡫 = significant decrease (p<0.05); ○ = no significant change (p>0.05).

TABLE A4. Continued

| Author(s) (year) and  study no. [#]^a^ | Country,  period and  population | Data and  sample size | Intervention  description | Outcome(s), measure(s)  and exposure | Study design and method | Key results and  time horizon of effects^b^ | Summary^c^ |
| --- | --- | --- | --- | --- | --- | --- | --- |
| van den Berg, Uhlendorff and Wolff (2014)  [46] | Germany (West)  2007-2009  Unemployment Benefit II (UB II) young (18-24 years) male claimants (mostly single without children) | Administrative data:  Integrated Employment Biographies (IEB) and UB II History Records  n = 71,667 spells | See description provided for Hillmann and Hohenleitner (2015) [13].  Wage compensation rate for UB II benefits:  45% for single person households. | *Adult outcomes*:  Employment  1. Entry into employment  *Exposure:*  First imposition of a sanction affects base benefit: 10% (missing appointment) or 100% (insufficient job-search/ refusal of job/ALMP). Duration: 90 days. Housing/accommodation costs and social security contributions unaffected. | Non-experimental:  Timing-of-events: mixed proportional hazards models | Both mild and strong sanctions lead to an increased transition rate to work. Strong sanctions increase the risk of entry into employment by 118.6% and mild sanctions by 36.6%.  *Time horizon*: long-term | 1. 🡩 |
| van den Berg, Uhlendorff and Wolff (2017)  [47] | Germany (West)  2007-2009  Unemployment Benefit II (UB II) claimants young (18-24 years) male claimants in single- and multi-person households | Administrative data:  Integrated Employment Biographies (IEB) and UB II History Records  n = 70,382 spells | See description provided for Hillmann and Hohenleitner (2015) [13].  Sanction rates: particularly high for young people as <25 year olds defined as a special target group. | *Adult outcomes*:  Employment  1. Entry into employment  Non-employment/ inactivity  2. Entry into non-employment/inactivity  Earnings/income  3. Daily wage  *Exposure:*  First imposition of a sanction affects base benefit: 100% (insufficient job-search / refusal of job/ALMP).  Duration: 90 days. Housing/accommodation costs and social security contributions unaffected;  Second sanction: 100% (complete benefit); duration: 90 days. | Non-experimental:  Timing-of-events: mixed proportional hazards models | For those in single-person households, first sanctions increase the risk of entry into employment by 109%, increase the risk of exit out of the labour force by 286% and decrease wages by 5.5%. Second sanctions add to the first two effects but not for wages.  For those in multiple-person households, first sanctions increase the risk of entry into employment by 70% and decrease wages by 3.3%. Second sanctions add to the first effect.  *Time horizon*: long-term | 1. 🡩  2. 🡩 (single-person),  ○  (multi-person)  3. 🡫 |

Notes: ^a^ Study number and reference for each study are reported in Table A4; ^b^ Time horizon of effects is classified as: short-term (0-12 months); medium-term (13-24 months), long-term (25+ months)

^c^ Direction of effect by outcome measure number: 🡩 = significant increase (p<0.05); 🡫 = significant decrease (p<0.05); ○ = no significant change (p>0.05).

TABLE A4. Continued

| Author(s) (year) and  study no. [#]^a^ | Country,  period and  population | Data and  sample size | Intervention  description | Outcome(s), measure(s)  and exposure | Study design and method | Key results and  time horizon of effects^b^ | Summary^c^ |
| --- | --- | --- | --- | --- | --- | --- | --- |
|  |  |  |  |  |  |  |  |
| van den Berg, van der Klaauw and van Ours (2004)  [48] | Netherlands (Rotterdam)  1994-1996  Social assistance (SA) benefits claimants who are unemployed and have a labour market history | Administrative data:  Rotterdam Welfare Agency  n = 7,978 individuals | SA eligibility is means-tested, i.e. depends on assets, income of partner, and the amount depends on household composition.  Wage compensation rate: 62% (single individual); 74% (married couple without children); 80% (married couple with two children); and 72% (single individual with two children). | *Adult outcomes*:  Employment:  1. Entry into employment  *Exposure:*  First imposition of a benefit sanction: 5%, 10% or 20%; Typical duration: 30-60 days; maximum duration: 180 days. | Non-experimental:  Timing-of-events: mixed proportional hazards models | Sanctions increase the risk of entry into employment by 148%. The effect of a sanction is not restricted to the period of benefits reduction, whilst the amount of benefits reduction seems to be unimportant for the sanction effect.  *Time horizon*: medium-term | 1. 🡩 |
| van der Klaauw and van Ours (2013)  [49] | Netherlands (Rotterdam)  2000-2003  Social assistance (SA) benefits claimants | Administrative data:  Rotterdam Welfare Agency  n = 30,527 spells; 28,039 individuals | SA benefits provide support to unemployed workers who are not entitled to any other social insurance benefits such as UI or disability insurance benefits. SA benefits are means-tested and related to the household composition, but not limited in duration.  Rotterdam bonus scheme (until 2002): individuals unemployed > 1 year entitled to a re-employment bonus if job lasts ≥ 6 months. | *Adult outcomes*:  Employment:  1. Entry into employment  *Exposure:*  First imposition of a benefit sanction of 5%, 10%, or 20% (typically 5%); typical duration: 30-60 days; maximum duration: 180 days. | Non-experimental:  Timing-of-events: mixed proportional hazards models | Financial sticks in the form of benefit sanctions were effective in stimulating the exit from welfare, while the financial carrots in the form of re-employment bonuses were not.  A sanction increases the risk of entry into employment by 21% for men and 47% for women. There is no strong association between the size of the sanction and its effect.  *Time horizon*: long-term | 1. 🡩 |

Notes: ^a^ Study number and reference for each study are reported in Table A4; ^b^ Time horizon of effects is classified as: short-term (0-12 months); medium-term (13-24 months), long-term (25+ months)

^c^ Direction of effect by outcome measure number: 🡩 = significant increase (p<0.05); 🡫 = significant decrease (p<0.05); ○ = no significant change (p>0.05).

TABLE A4. Continued

| Author(s) (year) and  study no. [#]^a^ | Country,  period and  population | Data and  sample size | Intervention  description | Outcome(s), measure(s)  and exposure | Study design and method | Key results and  time horizon of effects^b^ | Summary^c^ |
| --- | --- | --- | --- | --- | --- | --- | --- |
|  |  |  |  |  |  |  |  |
| Wu  (2008)  [50] | USA  (Wisconsin)  1997-2003  Low-income families | Wisconsin administrative data: Client Assistance for Re-employment and Economic Support (CARES) and Unemployment Insurance (UI).  n = 153,275 spells;  13,171 individuals | See description provided for Acs, Phillips and Nelson (2005) [2].  The welfare-to-work programme in Wisconsin is Wisconsin Works (W-2), which is structured to mirror entry-level employment: work requirements and benefit levels vary across four different tiers regardless of family size:  1.Low sanctions (<10% of monthly benefits);  2.Medium sanctions (10-50% of monthly benefits;  3.High sanctions (51-90% of monthly benefits);  4.Full sanctions (90+% of monthly benefits. | *Adult outcomes*:  Job quality:  1. Entry into a lower-earning job  2. Entry into a higher-earning job  Non-employment/  inactivity:  3. Exit from benefits  *Exposure:*  W-2 sanctions are directly related to the number of hours in which recipients fail to comply with W-2 work programme requirements; benefit reduction is minimum wage ($5.15 per hour) for each hour missed. Most sanctions represented a benefit reduction of less than 50%, and very few (<5%) were full (100%) sanctions. | Non-experimental:  Discrete-time competing risks models: multinomial logistic regression models | Families with children who are currently being sanctioned have a 18% higher risk of leaving welfare without a job and a 26% lower risk of leaving welfare with a higher earnings job.  However, the different levels of and duration of current sanctions affect welfare exit and employment outcomes differently. Those families receiving a small sanction (<10%) are significantly less likely to leave welfare regardless of post-welfare employment status, while the risk of leaving welfare without a job or with a lower earnings job increases with the severity and duration of the sanctions. Moreover, previous sanction experience appears to be significantly associated with an increased probability of leaving welfare without a job or with a low-earnings job, suggesting that sanctions have lagged effects on employment outcomes.  *Time horizon*: long-term | 1. ○  2. 🡫  3. 🡩 |

Notes: ^a^ Study number and reference for each study are reported in Table A4; ^b^ Time horizon of effects is classified as: short-term (0-12 months); medium-term (13-24 months), long-term (25+ months)

^c^ Direction of effect by outcome measure number: 🡩 = significant increase (p<0.05); 🡫 = significant decrease (p<0.05); ○ = no significant change (p>0.05).

TABLE A4. Continued

| Author(s) (year) and  study no. [#]^a^ | Country,  period and  population | Data and  sample size | Intervention  description | Outcome(s), measure(s)  and exposure | Study design and method | Key results and  time horizon of effects^b^ | Summary^c^ |
| --- | --- | --- | --- | --- | --- | --- | --- |
|  |  |  |  |  |  |  |  |
| Wu, Cancian and Wallace (2014)  [51] | USA  (Wisconsin)  1998-2001  Low-income families | Wisconsin administrative data  n = 1,599 individuals | See description provided for Acs, Phillips and Nelson (2005) [2].  The welfare-to-work programme in Wisconsin is Wisconsin Works (W-2): cash benefits depend on hours worked, and payments do not vary by family size. Claimants are placed in tiers with requirements depending on employment preparedness. | *Adult outcome*:  Job quality:  1. Entry into below benefit-level job  2. Entry into above benefit-level job  Non-employment/ inactivity:  3. Exit from benefits  *Exposure:*  W-2 sanctions are directly related to the number of hours in which recipients fail to participate in assigned activities; benefit reduction is minimum wage ($5.15 per hour) for each hour missed. Most sanctions involve benefit reduction of < 50%. Sanctions are open ended until compliance (generally < 90 days;  20% > 90 days). | Non-experimental:  Discrete-time competing risk models: multinomial logistic regression models | Sanctions are significantly associated with an increase in the likelihood of making a transition to an above-benefits job.  *Time horizon*: long-term | 1. ○  2. 🡩  3. ○ |
| Yu (2001)  [52] | USA  1998  Low-income families/TANF benefits recipients | State-level aggregated data, various sources  n = 35 state observations | See description provided for Acs, Phillips and Nelson (2005) [2]. | *Adult outcomes:*  Benefits:  1. Welfare caseload  Exposure:  % welfare recipients experiencing benefit reductions  (at state-level) | Non-experimental:  Weighted Ordinary Least Squares (OLS) regression model | Sanction policies are significantly associated with a decrease in welfare caseload by 52%.  Time horizon: short-term | 1. 🡫 |

Notes: ^a^ Study number and reference for each study are reported in Table A4; ^b^ Time horizon of effects is classified as: short-term (0-12 months); medium-term (13-24 months), long-term (25+ months)

^c^ Direction of effect by outcome measure number: 🡩 = significant increase (p<0.05); 🡫 = significant decrease (p<0.05); ○ = no significant change (p>0.05).

TABLE A4. Continued

| Author(s) (year) and  study no. [#]^a^ | Country,  period and  population | Data and  sample size | Intervention  description | Outcome(s), measure(s)  and exposure | Study design and method | Key results and  time horizon of effects^b^ | Summary^c^ |
| --- | --- | --- | --- | --- | --- | --- | --- |
| Panel a2: Labour market outcomes: Quasi-experimental design | | | | | | | |
| Arni and Schiprowski (2016)  [53] | Switzerland  (14 out of 26 cantons)  2011-2014  Unemployment Insurance (UI) claimants aged 20-55 years, excluding part-time workers and disabled claimants | Swiss UI benefits data  n = (up to) 16,218 individuals | See description provided for Arni and Schiprowski (2015) [4].  Those ineligible for UI can receive social assistance (SA), which is equivalent to 76% UI benefits.  Claimants are required to provide evidence of a set number of monthly job applications. | *Adult outcomes:*  Employment:  1. Entry into employment  2. Entry into employment (individual effort only)  Job stability:  3. Entry into stable employment (lasting 12+ months post UI benefits exit; individual effort only)  Long-term non-employment/inactivity:  4. Re-entry into non-employment after an employment spell (lasting <12 mths post UI benefits exit; individual effort only; entry into unstable job)  Earnings/income:  5. Earnings from employment post-UI exit  6. Total earnings post-UI benefits exit  *Exposure:*  The warning effect of a sanction: receiving a notification for non-compliance (failure to submit evidence of job applications by monthly deadline) with increased risk of receiving a benefit sanction vs other non-compliance notifications, where the risk of receiving a sanction stayed constant. | Quasi-experimental:  Difference-in-difference: proportional hazards regression models with fixed effects | Stricter sanction enforcement for non-compliance increased transitions into employment only through individual effort by 24% within the first 6 months after UI entry; the effect was not significant in the longer term. Most of the transitions were to unstable jobs, with a 4% probability of unemployment within 12 months. For early job-finders there was a negative and significant effect on total earnings due to more frequent transitions on/off benefits, while a non-significant effect was found for the average job seeker. The average earnings over 18 months were up to 441 CFH lower for people who found a job within 6 months through their own effort.  *Time horizon:* short- to medium-term | 1. ○  2. 🡩  3. ○  4. 🡩  5. ○  6. 🡫 |

Notes: ^a^ Study number and reference for each study are reported in Table A4; ^b^ Time horizon of effects is classified as: short-term (0-12 months); medium-term (13-24 months), long-term (25+ months)

^c^ Direction of effect by outcome measure number: 🡩 = significant increase (p<0.05); 🡫 = significant decrease (p<0.05); ○ = no significant change (p>0.05).

TABLE A4. Continued

| Author(s) (year) and  study no. [#]^a^ | Country,  period and  population | Data and  sample size | Intervention  description | Outcome(s), measure(s)  and exposure | Study design and method | Key results and  time horizon of effects^b^ | Summary^c^ |
| --- | --- | --- | --- | --- | --- | --- | --- |
|  |  |  |  |  |  |  |  |
| Arni and Schiprowski (2019)  [54] | Switzerland  (4 out of 26 cantons)  2010-2014  Unemployment Insurance (UI) claimants aged 20-55 years, excluding part-time workers and disabled claimants | Administrative data:  Swiss UI benefit data, 2010-2017;  Social security data, 2010-2015.  n = (up to) 96,833 individuals | See description provided for Arni and Schiprowski (2015) [4].  Potential duration of UI benefits: 1.5 years.  Sanctions: 100% of UI benefit; duration: 5.5 days on average. | *Adult outcomes:*  Job stability:  1. Exit from employment spell following non-employment  Non-employment/ inactivity  2. Exit from unemployment  3. Exit from non-employment  Job quality (earnings):  4. Average monthly earnings from re-employment during the first 3 months from exit from non-employment  *Exposure:*  Threat of sanctions through binding job-search requirements | Quasi -experimental:  Instrumental Variable (IV) estimation:  Two-Stage Least Squares (2SLS) regression models with fixed effects | The requirement to send one additional job application per month does not have a statistically significant effect at 0.05 level on the duration of re-employment spells. Additional required monthly applications increase the rate of exit from both unemployment and non-employment by 3% (reducing unemployment and non-employment spells by 7 days and 10 days, respectively). No significant effect was found for re-employment wages.  *Time horizon:* short-term | 1. ○  2. 🡩  3. 🡩  4. ○ |

Notes: ^a^ Study number and reference for each study are reported in Table A4; ^b^ Time horizon of effects is classified as: short-term (0-12 months); medium-term (13-24 months), long-term (25+ months)

^c^ Direction of effect by outcome measure number: 🡩 = significant increase (p<0.05); 🡫 = significant decrease (p<0.05); ○ = no significant change (p>0.05).

TABLE A4. Continued

| Author(s) (year) and  study no. [#]^a^ | Country,  period and  population | Data and  sample size | Intervention  description | Outcome(s), measure(s)  and exposure | Study design and method | Key results and  time horizon of effects^b^ | Summary^c^ |
| --- | --- | --- | --- | --- | --- | --- | --- |
|  |  |  |  |  |  |  |  |
| Boockmann, Thomsen and Walter (2014)  [55] | Germany  2006-2007  Unemployment Benefits II (UB II) claimants | Linked administrative-survey data: Unique survey, 2007;  Administrative data from Federal Employment Agency (FEA), 2006-2007  n = 15,361 individuals | See description provided for Hillmann and Hohenleitner (2015) [13] | *Adult outcomes*:  Non-employment/ inactivity:  1.Exit benefits  *Exposure*:  First imposition of a sanction (first infringement entails a 10% or a 30% reduction of UB payment; duration: 90 days) | Quasi-experimental:  Instrumental Variable (IV) estimation | A tightening of the sanction regime increases the probability of leaving the welfare system by 58 to 68 percentage points.  *Time horizon*: short-term | 1.🡩 |
|  |  |  |  |  |  |  |  |

Notes: ^a^ Study number and reference for each study are reported in Table A4; ^b^ Time horizon of effects is classified as: short-term (0-12 months); medium-term (13-24 months), long-term (25+ months)

^c^ Direction of effect by outcome measure number: 🡩 = significant increase (p<0.05); 🡫 = significant decrease (p<0.05); ○ = no significant change (p>0.05).

TABLE A4. Continued

| Author(s) (year) and  study no. [#]^a^ | Country,  period and  population | Data and  sample size | Intervention  description | Outcome(s), measure(s)  and exposure | Study design and method | Key results and  time horizon of effects^b^ | Summary^c^ |
| --- | --- | --- | --- | --- | --- | --- | --- |
|  |  |  |  |  |  |  |  |
| Cockx and Dejemeppe (2007)  [56] | Belgium (Brussels, Flanders and Wallonia regions)  2004 – 2005  Long-term Unemployment Insurance (UI) claimants  (13+ months), aged 25-34 years | Administrative data:  Federal Unemployment Agency (UA) benefit claims data  n = (up to) 37,668 spells;  6,278 individuals | In July 2004, the monitoring of the Unemployment Insurance (UI) scheme was intensified through the introduction of a notification letter at 14 months informing claimants aged <30 years that they must seek work and will be called for an interview in 8 months to evidence this. Job counselling and training were introduced simultaneously. Procedures following receipt of letter vary across regions.  UI benefits:  Gross wage compensation rate: <55%-60% on average, depending on whether cohabiting with a partner.  Sanctions imposed for failure to evidence sufficient job seeking activity after July 2004. First failure: 4 months. Second failure: permanent. | *Adult outcomes:*  Employment:  1. Employed (not in receipt of UI benefits or employed while on UI benefits) by claimants’ group  Job stability:  2. Employed for 3+ months by claimants’ group  3. Employed for 6+ months by claimants’ group  Job quality:  4. Low-wage part-time employed  *Exposure:*  Threat of a sanction: receipt of letter after 13 months notifying claimants that their job search will be monitored and they will have to attend an interview 8 months after notification, risking a sanction for insufficient searching. The letter was sent only to claimants aged <30 years. In Brussels counselling was provided after notification; in Flanders counselling was not provided; in Wallonia both counselling and training were provided. | Quasi-experimental:  Fuzzy regression discontinuity design: Two-Stage Least Squares (2SLS) procedure with estimated propensity scores | The pure threat effect of intensified monitoring without counselling increases the probability of employment only for more highly educated workers (Flanders (F), 12%). However, it also increases the probability of transition to more stable employment (3+ and 6+ months) and lower quality jobs (part-time employment with income supplement) for this group (14%, 12% and 3%, respectively).  Counselling provided after notification increases the probability of employment for women (Brussels (B), 13%). However, this also increases transition to employment lasting for 3+ months only for women (9%). Similar results for women were recorded also in Wallonia (W), where both counselling and training were provided (8%) although no significant effect was recorded for job quality outcomes (2-4).  *Time horizon:* short-term | 1. 🡩 (B),  🡩 (F)  🡩 (W)  2. 🡩 (B),  🡩 (F)  ○ (W)  3. ○ (B),  🡩 (F)  ○ (W)  4. ○ (B),  🡩 (F)  ○ (W) |

Notes: ^a^ Study number and reference for each study are reported in Table A4; ^b^ Time horizon of effects is classified as: short-term (0-12 months); medium-term (13-24 months), long-term (25+ months)

^c^ Direction of effect by outcome measure number: 🡩 = significant increase (p<0.05); 🡫 = significant decrease (p<0.05); ○ = no significant change (p>0.05).

TABLE A4. Continued

| Author(s) (year) and  study no. [#]^a^ | Country,  period and  population | Data and  sample size | Intervention  description | Outcome(s), measure(s)  and exposure | Study design and method | Key results and  time horizon of effects^b^ | Summary^c^ |
| --- | --- | --- | --- | --- | --- | --- | --- |
| Cockx and Dejemeppe (2012)  [57] | Belgium (Flanders)  2004-2005  Long-term Unemployment Insurance (UI) claimants  (13+ months), aged 25-35 years | Administrative data:  Federal Unemployment Benefit (UB) agency, Regional Public Employment Services (PES) and Crossroads Bank for Social Security data.  n = 1,490 individuals | See description provided for Cockx and Djemeppe (2007) [56].  Requirements prior to the interview are stated quite vaguely; claimants must evidence 'regular' job seeking.  In the event of non-compliance with requirements imposed after first negative evaluation benefits are withdrawn entirely for 4 months. Subsequent non-compliance leads to total loss of benefits but social assistance is still available.  If there is a positive evaluation at the first interview, the subsequent interview is delayed by up to 16 months. | *Adult outcomes:*  Employment:  1.Entry into employment at 8 months post-notification receipt  Non-employment/  inactivity:  2.Entry into training (e.g. job-search assistance and return to standard education)  3. Entry into inactivity  *Exposure:*  Threat effect of receipt of notification letter requiring attendance at job monitoring interview 8 months after receipt. | Quasi-experimental:  Fuzzy regression discontinuity design: Ordinary Least Squares (OLS) and Two-Stage Least Squares (2SLS) procedure with estimated propensity scores | No effect at 5% significance level was found for notifications of job-search monitoring, backed with threat of sanctions on transitions to employment, training participation and labour market exits.  *Time horizon:* short-term | 1. ○  2. ○  3. ○ |

Notes: ^a^ Study number and reference for each study are reported in Table A4; ^b^ Time horizon of effects is classified as: short-term (0-12 months); medium-term (13-24 months), long-term (25+ months)

^c^ Direction of effect by outcome measure number: 🡩 = significant increase (p<0.05); 🡫 = significant decrease (p<0.05); ○ = no significant change (p>0.05).

TABLE A4. Continued

| Author(s) (year) and  study no. [#]^a^ | Country,  period and  population | Data and  sample size | Intervention  description | Outcome(s), measure(s)  and exposure | Study design and method | Key results and  time horizon of effects^b^ | Summary^c^ |
| --- | --- | --- | --- | --- | --- | --- | --- |
|  |  |  |  |  |  |  |  |
| Danielson and Klerman (2008)  [58] | USA  1990-2005  Low-income families | Administrative data:  Welfare Rules Database,  1990-2005  n = 9,359 state-months observations | See description provided for Acs, Phillips and Nelson (2005) [2]. | *Adult outcomes*:  Benefits:  1. Benefit caseload (all benefit recipients)  *Exposure*:  Imposition of full-family (100%) benefit sanctions (immediate closure or gradual i.e. case is closed only after repeated non-compliance; state-level indicator. | Quasi-experimental  Difference-in-differences (DiD) with fixed effects | The state-level introduction of full-family sanction policies reduces the welfare caseload three years after implementation by 36% (immediate full-family sanction) and 13% (gradual full-family sanctions).  *Time horizon*: long-term | 1. 🡫 |
| Fording, Schram and Soss (2013)  [59] | USA  (Florida)  2000-2003  Low-income families | Administrative data:  Welfare Transition Programme with earnings data from UI benefits records.  n = 36,319 individuals | See description provided for Acs, Phillips and Nelson (2005) [2].  The TANF programme in Florida is the Welfare Transition programme: it adopted some of the strictest time limits and work requirements in the US, and restricted possibilities for exemptions from sanctions – sanction rates are high compared with other states with full-family sanctions. | *Adult outcome*:  Earnings/income:  1. Change in earnings up to a year after benefit exit  *Exposure:*  Immediate full-family (100%) benefit sanctions; duration: 10 days (first non-compliance), 30 and 90 days (subsequent non-compliance instances). Sanctions are accompanied by reduction in food stamps. | Quasi-experimental:  Difference-in-differences (DiD) with Propensity Score Matching (PSM) | Sanctioning has a negative effect on earnings among TANF clients, up to a year after benefit exit. The effect is both statistically and substantively significant. The effect is consistent across racial groups, larger among clients with at least 12 years of schooling (compared with <12 years of schooling), and generally increases with the frequency of sanctioning.  *Time horizon*: short-term | 1. 🡫 |

Notes: ^a^ Study number and reference for each study are reported in Table A4; ^b^ Time horizon of effects is classified as: short-term (0-12 months); medium-term (13-24 months), long-term (25+ months)

^c^ Direction of effect by outcome measure number: 🡩 = significant increase (p<0.05); 🡫 = significant decrease (p<0.05); ○ = no significant change (p>0.05).

TABLE A4. Continued

| Author(s) (year) and  study no. [#]^a^ | Country,  period and  population | Data and  sample size | Intervention  description | Outcome(s), measure(s)  and exposure | Study design and method | Key results and  time horizon of effects^b^ | Summary^c^ |
| --- | --- | --- | --- | --- | --- | --- | --- |
| Moffitt (2003)  [60] | USA  (Boston, MA, Chicago, IL, and San Antonio, TX)  1999-2001  Low-income families/lone parents living in low and moderate-income neighbourhoods | Survey data:  Three-City Study,  1999-2001  n =2,136 individuals | See description provided for Acs, Phillips and Nelson (2005) [2]. Massachusetts (Boston) is a Work First state requiring work activity within 60 days and moderate number of exemptions from the requirement. Sanctions are imposed for work non-compliance but also for other reasons. Sanctions are initially imposed only on the adult but can escalate to full-family sanctions.  Illinois (Chicago) is not a Work First state, requiring work only within the first 2 years of benefit receipt; has large number of allowable activities/  exemptions. Sanctions are imposed for different reasons and start at 50% of benefit (partial) and escalate to full (100%) sanctions.  Texas (San Antonio) is a Work First state that requires immediate work. Modest number of activities/exemptions. Sanctions imposed for non-compliance; accumulate at $78/month until a cap is reached, but always partial. | *Adult outcome*:  Non-employment/ inactivity:  1.Being off benefits at 24 months  Benefits:  2.Being on benefits at 24 months  *Exposure*:  Take-up effect of participating into the New Deal for Young people (NDYD) programme implying a threat of sanctions. | Quasi-experimental:  Instrumental Variable (IV) estimation | Through work requirements, sanction have a positive threat effects on transition off welfare benefits, while results are not significant for entry into welfare benefits.  *Time horizon*: medium-term | 1. ○  2. 🡩 |

Notes: ^a^ Study number and reference for each study are reported in Table A4; ^b^ Time horizon of effects is classified as: short-term (0-12 months); medium-term (13-24 months), long-term (25+ months)

^c^ Direction of effect by outcome measure number: 🡩 = significant increase (p<0.05); 🡫 = significant decrease (p<0.05); ○ = no significant change (p>0.05).

TABLE A4. Continued

| Author(s) (year) and  study no. [#]^a^ | Country,  period and  population | Data and  sample size | Intervention  description | Outcome(s), measure(s)  and exposure | Study design and method | Key results and  time horizon of effects^b^ | Summary^c^ |
| --- | --- | --- | --- | --- | --- | --- | --- |
|  |  |  |  |  |  |  |  |
| National Audit Office (2016b)  [61] | UK  (Great Britain)  2014-2015  Unemployment Insurance (UI) and disability-related benefit claimants (Jobseeker’s Allowance (JSA) and Employment and Support Allowance (ESA))/ participating in Work Programme (WP) | Linked administrative data: Work and Pensions Longitudinal Study (WPLS) from the Department for Work and Pensions (DWP)  n = (up to) 225,956 individuals | In June 2011, the Work Programme (WP) was implemented to provide employment support for long-term benefit claimants. The programme is run by service provi  ders with discretionary powers on which activities to make compulsory. Within each area, claimants are randomly allocated to providers in ‘contract package areas’ by DWP. The Work Programme was discontinued in April 2017. In October 2012 a New Sanctions Regime was implemented with the introduction of 100% sanctions for between 4 weeks and 3 years for all benefit claimants. | *Adult outcomes*:  Employment:  1. Employment status  Job stability:  2. Number of days in employment  Benefits:  3. Number of days claiming JSA/ESA benefits  Non-employment/ inactivity:  4. Number of days not claiming nor employed  Earnings/income:  5. Earnings from employment  *Exposure*:  Imposition of first sanction. Sanctions can be either partial of full (100%) benefit sanctions, lasting between 4 weeks and 3 years. | Quasi-experimental:  Instrumental Variable (IV) estimation: Two-Stage Least Squares (2SLS) models | Benefit sanctions increase both the probability of being in employment by 98 percentage points and the time in employment by 225 days among JSA benefit claimants one year after initial sanctions. Among ESA benefit claimants a significant reduction by 63 percentage points and 40 days are observed for the same employment outcomes. A significant reduction by 468 days is found for benefit claiming among JSA recipients, while an increase of 88 days if found among ESA recipients. For both JSA and ESA recipients, sanctions increase the time neither employed nor claiming by 236 and 23 days, respectively. A decrease in earnings from employment by 2,810 pounds is found among ESA benefit claimants while the effect is not significant one year after the first sanction among JSA benefit claimants.  *Time horizon*: short-term | 1. 🡩 (JSA)  🡫(ESA)  2. 🡩 (JSA)  🡫(ESA)  3. 🡫 (JSA)  🡩(ESA)  4. 🡩 (JSA)  🡩(ESA)  4. ○ (JSA)  🡫(ESA) |

Notes: ^a^ Study number and reference for each study are reported in Table A4; ^b^ Time horizon of effects is classified as: short-term (0-12 months); medium-term (13-24 months), long-term (25+ months)

^c^ Direction of effect by outcome measure number: 🡩 = significant increase (p<0.05); 🡫 = significant decrease (p<0.05); ○ = no significant change (p>0.05).

TABLE A4. Continued

| Author(s) (year) and  study no. [#]^a^ | Country,  period and  population | Data and  sample size | Intervention  description | Outcome(s), measure(s)  and exposure | Study design and method | Key results and  time horizon of effects^b^ | Summary^c^ |
| --- | --- | --- | --- | --- | --- | --- | --- |
|  |  |  |  |  |  |  |  |
| Taulbut, Mackay and McCartney  (2018)  [62] | United Kingdom  (Great Britain)  2001-2014  Unemployment Insurance (UI) benefit claimants (Job Seeker’s Allowance (JSA) working-age claimants) | Aggregate published data from various sources.  n = n/a | Since April 2010: Increase in penalties for missing an advisor appointment without good reason.  June 2011: Implementation of Work Programme, which replaced most previous DWP-funded employment support for long-term benefit claimants.  October 2012: New Sanctions Regime with the introduction of 100% sanctions for between 4 weeks and 3 years for all JSA claimants. | *Adult outcomes:*  Employment  1. JSA off-flows into employment  *Exposure:*  Increases in severity of imposed sanctions and expansion of reasons for incurring a sanction. Implementation of new employment support programme (Work Programme) for long-term benefit claimants. | Quasi-experimental:  Interrupted time-series regression models | Since October 2012, the increase in the severity and intensity of sanctions decreased the number of movements off benefits and into employment in the short-run while the change in the trend was not significant. No significant effect was found for the cumulative impact of the three policies (April 2010 increase in penalties, June 2011 Work Programme and October 2012 New Sanctions Regimes).  *Time horizon:* long-term | 1. 🡫 |

Notes: ^a^ Study number and reference for each study are reported in Table A4; ^b^ Time horizon of effects is classified as: short-term (0-12 months); medium-term (13-24 months), long-term (25+ months)

^c^ Direction of effect by outcome measure number: 🡩 = significant increase (p<0.05); 🡫 = significant decrease (p<0.05); ○ = no significant change (p>0.05).

TABLE A4. Continued

| Author(s) (year) and  study no. [#]^a^ | Country,  period and  population | Data and  sample size | Intervention  description | Outcome(s), measure(s)  and exposure | Study design and method | Key results and  time horizon of effects^b^ | Summary^c^ |
| --- | --- | --- | --- | --- | --- | --- | --- |
|  |  |  |  |  |  |  |  |
| Panel a3: Labour market outcomes: Experimental design | | | | | | | |
| Knab et al. (2000)  [63] | USA  (Riverside, CA;  Grand Rapids, MI)  1991-1994  AFDC recipients (Jobs Opportunities and Basic Skills Training (JOBS) programme) | Administrative data:  National Evaluation of Welfare-to-Work Strategies  n = 23,177 individuals | The Job Opportunities and Basic Skills Training (JOBS) programme was introduced with the Family Support Act (FSA) of 1988 and was designed to move welfare recipients into the labour market. JOBS mandate allows benefit recipients to be employed part-time. | *Adult outcomes*:  Employment:  1. % ever employed  Benefits:  2. Months received AFDC  Earnings/income:  3. Total earnings ($)  *Exposure*:  Threat of a sanction. Sanctions for non-compliance typically entail a 19% reduction of benefits for single-parent households; 100% reduction for two-parent households. | Experimental:  Random assignment: Weighted Ordinary Least Squares (OLS) regression models | JOBS mandate appears to have strong positive short-term effects on both employment and earnings outcomes (12 and 31 percent difference, respectively, during the first 12 months) which tend to disappear in the medium term (24 months after the random assignment exercise).  *Time horizon*: short- to medium-term | 1. 🡩 (yr 1)  ○ (yr 2)  2. ○ (yr 1)  ○ (yr 2)  3. 🡩 (yr 1)  ○ (yr 2) |

Notes: ^a^ Study number and reference for each study are reported in Table A4; ^b^ Time horizon of effects is classified as: short-term (0-12 months); medium-term (13-24 months), long-term (25+ months)

^c^ Direction of effect by outcome measure number: 🡩 = significant increase (p<0.05); 🡫 = significant decrease (p<0.05); ○ = no significant change (p>0.05).

TABLE A4. Continued

| Author(s) (year) and  study no. [#]^a^ | Country,  period and  population | Data and  sample size | Intervention  description | Outcome(s), measure(s)  and exposure | Study design and method | Key results and  time horizon of effects^b^ | Summary^c^ |
| --- | --- | --- | --- | --- | --- | --- | --- |
|  |  |  |  |  |  |  |  |
| Michaelidis and Mueser (2018)  [64] | USA  (Nevada: metropolitan areas of Las Vegas-Henderson-Paradise and Reno)  2009-2011  Unemployment Insurance (UI) benefits claimants who are eligible for participating in the re-employment programme | Linked administrative data:  UI benefits claims data;  UI wage records;  Employment Services data  n =31,793 individuals | In 2009, in order to facilitate the recovery from the post-2008 economic recession, the American Recovery and Reinvestment Act (ARRA) made available large investment funds to enhance the capacity of states to provide re-employment services.  In Nevada, the re-employment programme required UI recipients to: (1) undergo an eligibility review to confirm whether they were qualified for benefits and were actively searching for a job and, if deemed eligible, (2) received job-counselling services. | *Adult outcomes*:  Employment:  1. Whether positive earnings in quarters 1-6  Benefits:  2. Weeks in receipts of UI and Emergency Unemployment Compensation (EUC) (additional payment for UI claimants who exhausted their regular UI benefits)  Earnings/income:  3. Total earnings ($) in quarters 1-6  *Exposure*:  Threat of benefit sanctions (100% disqualifications) for failure to attend a meeting and for not attending job counselling services. | Experimental:  Random assignment: Ordinary Least Squares (OLS) regression models with fixed effects | Participants in the re-employment programme that were exposed to the threat effects of sanctions reduced the amount of time in receipt of unemployment benefits by 4.4 weeks, a 12% reduction relative to the control group. Taking part in the re-employment programme also increased employment rates by 7 and 8.2 percentage points in the first and second quarters, respectively. The programme's effect on employment gradually declined over time but remained positive and statistically significant. The programme had also positive effects on earnings, with total earnings increasing by $2,607 (18 percent increase compared to the control group)  *Time horizon*: short- to medium-term | 1. 🡩  2. 🡫  3. 🡩 |

Notes: ^a^ Study number and reference for each study are reported in Table A4; ^b^ Time horizon of effects is classified as: short-term (0-12 months); medium-term (13-24 months), long-term (25+ months)

^c^ Direction of effect by outcome measure number: 🡩 = significant increase (p<0.05); 🡫 = significant decrease (p<0.05); ○ = no significant change (p>0.05).

TABLE A4. Continued

| Author(s) (year) and  study no. [#]^a^ | Country,  period and  population | Data and  sample size | Intervention  description | Outcome(s), measure(s)  and exposure | Study design and method | Key results and  time horizon of effects^b^ | Summary^c^ |
| --- | --- | --- | --- | --- | --- | --- | --- |
|  |  |  |  |  |  |  |  |
| Micklewright and Nagy (2010)  [65] | Hungary  (6 out of 20 counties)  2003  Unemployment Insurance (UI) benefits claimants aged <50 years, with 75-179 days of UI entitlement | Linked administrative data (unspecified sources)  n =2,134 individuals;  28 out of 170 local employment offices | In Autumn 2003, a new legislation was introduced requiring UI benefit claimants to make at least one visit to the local employment office every 3 months. Previous legislation only required visits to be ‘regular’ while the frequency was left at the discretion of the offices.  There is a high variation in practice across employment offices within counties.  Participants in the treatment group were subject to increased monitoring and required to visit the employment office every three months and were questioned about their job-search activities. The control group faced no questions on job search.  Assignment to treatment or control groups was conducted on the basis of the date of birth of UI claimants. | *Adult outcomes*:  Employment  1. Entry into employment  *Exposure*:  Threat of benefit sanctions for failure to attend a meeting and potentially for failure to report job-search activities (the latter were not imposed). Temporary sanctions (until compliance): usually imposed for missing a meeting with the employment office; permanent sanctions: for rejecting a job offer or being fired (for second or subsequent incidence of non-compliance). | Experimental:  Randomised Control Trial (RCT): flexible parametric hazard models, accounting for clustering at the employment office level | The threat of sanctions increased the risk of entry into employment by 50% for women aged 30+ years, whereas the effect was not significant for younger women and for men  *Time horizon*: short-term | 1. 🡩 |

Notes: ^a^ Study number and reference for each study are reported in Table A4; ^b^ Time horizon of effects is classified as: short-term (0-12 months); medium-term (13-24 months), long-term (25+ months)

^c^ Direction of effect by outcome measure number: 🡩 = significant increase (p<0.05); 🡫 = significant decrease (p<0.05); ○ = no significant change (p>0.05).

TABLE A4. Continued

| Author(s) (year) and  study no. [#]^a^ | Country,  period and  population | Data and  sample size | Intervention  description | Outcome(s), measure(s)  and exposure | Study design and method | Key results and  time horizon of effects^b^ | Summary^c^ |
| --- | --- | --- | --- | --- | --- | --- | --- |
|  |  |  |  |  |  |  |  |
| Olson, Schexnayder and O’Shea (1997)  [66] | USA  (Hawaii)  1995-1996  Low income families/  AFDC recipients/ participants in Jobs Opportunities and Basic Skills Training (JOBS) WORKS! programme | Administrative data from Hawaii Departments of Human Services, and Labor and Industrial Relations  n = (up to) 3,294 individuals | See description provided for Knab et al. (2000) [63].  Hawaii received a waver from the US Department of Health and Human Services to implement a pilot project which will become JOBS WORKS! Programme for JOBS clients. AFDC recipients participating in the programme who are included in the treatment group are required to take 18 hours of work-related activities.  JOBS WORKS! offered immediate job search and job readiness activities as well as job development and placement services to eligible AFDC benefit recipients. | *Adult outcomes*:  Employment:  1. Whether employed after selection  2. Months of employment for those employed  Benefits:  3. Total AFDC benefits for 21 months after selection  Non-employment/  inactivity:  4. Whether exited AFDC after selection  Long-term benefits:  5. Whether returned on AFDC within 21 months after selection  Earnings/income:  6. Earnings ($) for those employed  7. Total family income ($) for 21 months after selection  *Exposure*:  Threat of a benefit sanction. | Experimental:  Random assignment: Weighted Ordinary Least Squares (OLS) regression models | The threat of sanctions had significant positive impacts on both employment and earnings of AFDC participants. The adjusted positive net effect of programme participation on employment status and length of employment was 4.5% and approximately 10%, respectively. Earnings increased by approximately 30% over the 21-month follow-up period where no effect was observed for total family income. The programme also reduced participants’ dependence on benefits in the medium term, with a decrease in benefit payments by 6% and an increase in benefit exits by 5%. No effect was found on re-entry into benefits for those who exited AFDC.  *Time horizon*: short- to medium-term | 1. 🡩  2. 🡩  3. 🡫  4. 🡩  5. ○  6. 🡩  7. ○ |

Notes: ^a^ Study number and reference for each study are reported in Table A4; ^b^ Time horizon of effects is classified as: short-term (0-12 months); medium-term (13-24 months), long-term (25+ months)

^c^ Direction of effect by outcome measure number: 🡩 = significant increase (p<0.05); 🡫 = significant decrease (p<0.05); ○ = no significant change (p>0.05).

TABLE A4. Continued

| Author(s) (year) and  study no. [#]^a^ | Country,  period and  population | Data and  sample size | Intervention  description | Outcome(s), measure(s)  and exposure | Study design and method | Key results and  time horizon of effects^b^ | Summary^c^ |
| --- | --- | --- | --- | --- | --- | --- | --- |
|  |  |  |  |  |  |  |  |
| Riccio and Hasenfeld (1996)  [67] | USA  (6 out of 58 counties in California – not randomly sampled)  1988-1993  Lone parents with children aged 6 years or older/  AFDC recipients participating in Greater Avenues to Independence (GAIN) programme | Linked administrative-survey data: GAIN administrative case records; Survey conducted by Manpower Demonstration Research Corporation (MDRC);  n = ~ 23,000 individuals | See description provided for Knab et al. (2000) [63].  The Greater Avenues to Independence (GAIN) programme is a type of Job Opportunities and Basic Skills Training (JOBS) programme available in California. It offers a mix of job search, education, training and work experience activities and provides basic education for those lacking reading and mathematics skills or proficiency in English. Childcare and transportation are available if required. Participants in specific circumstances (e.g. in part-/full-time work, illness) may be temporarily deferred or exempted from participation. | *Adult outcomes*:  Benefits:  1. Average reduction in AFDC payments  Earnings/income:  2. Average earnings  *Exposure*:  Formal penalty rate measuring the extent to which programme staff relied on GAIN’S formal penalty process (i.e. procedure leading to sanctions); formal penalty rates vary across counties and local employment offices | Experimental:  Random assignment:  Correlation coefficients at county or local employment office level | No statistically significant correlation was found between formal penalty rate and average earnings and reduction in AFDC payments at both county- and office-levels three years after initial random assignment.  *Time horizon*: long-term | 1. ○  2. ○ |

Notes: ^a^ Study number and reference for each study are reported in Table A4; ^b^ Time horizon of effects is classified as: short-term (0-12 months); medium-term (13-24 months), long-term (25+ months)

^c^ Direction of effect by outcome measure number: 🡩 = significant increase (p<0.05); 🡫 = significant decrease (p<0.05); ○ = no significant change (p>0.05).

TABLE A4. Continued

| Author(s) (year) and  study no. [#]^a^ | Country,  period and  population | Data and  sample size | Intervention  description | Outcome(s), measure(s)  and exposure | Study design and method | Key results and  time horizon of effects^b^ | Summary^c^ |
| --- | --- | --- | --- | --- | --- | --- | --- |
|  |  |  |  |  |  |  |  |
| Scrivener and Walter (2001)  [68] | USA  (Columbus, Ohio)  1992-1997  Lone parents with youngest child 3 years or older/ AFDC recipients participating in the Job Opportunities and Basic Skills Training (JOBS) mandatory programme | Linked administrative-survey data:  National Evaluation of Welfare-to-Work Strategies (NEWWS),  1992-1997  n = 7,242 individuals | See description provided for Knab et al. (2000) [63].  As part of the Job Opportunities and Basic Skills Training (JOBS) mandatory programme, Columbus’s operated an integrated JOBS programme (combining income maintenance and employment/training services) and a traditional JOBS programme (with separated services). Participants in both programmes (treatment groups) received the same services. | *Adult outcomes*:  Employment:  1. Ever employed (%)  2. Quarters employed  Benefits:  3. Ever received AFDC  4. Months received AFDC  5. AFDC amount ($)  Earnings/income:  6. Earnings ($)  *Exposure*:  Participants in both treatment groups (integrated and traditional JOBS programmes) were subject to benefit sanctions and received job-related services. The control group could access services independently if they wished and may have been subject to less severe sanctions under AFDC.  Average benefit sanction: 18% reduction of the monthly payment. Duration: first sanction: until compliance; second sanction: minimum 3 months; third sanction: minimum 6 months. | Experimental:  Random assignment | During the three years following random assignment, participation in the integrated and traditional programmes produced small increases in both employment rates (2.2% and 2.6%, respectively) and average length of time in employment (0.23 and 0.29 quarters, respectively). No significant effect was found for rates of benefit receipt, although programme participation significantly decreased welfare receipt after the first year of the follow-up period. Both the number of months and amount of AFDC receipt decreased significantly during the three-year follow-up period (1.7 and 2.6 months in the first case for integrated and traditional programmes, respectively, corresponding to a reduction of 8% and 12%, and 1,079$ and 816$ in the second case, respectively, corresponding to a reduction of 11% and 15%). Participants in the integrated programme had an increase in earnings of 1,181$ (corresponding to a 10% increase) while those assigned to the traditional programme had an increase of 1,000$ (8%).  *Time horizon*: long-term | 1. 🡩  2. 🡩  3. ○  4. 🡫  5. 🡫  6. 🡩 |

Notes: ^a^ Study number and reference for each study are reported in Table A4; ^b^ Time horizon of effects is classified as: short-term (0-12 months); medium-term (13-24 months), long-term (25+ months)

^c^ Direction of effect by outcome measure number: 🡩 = significant increase (p<0.05); 🡫 = significant decrease (p<0.05); ○ = no significant change (p>0.05).

TABLE A4. Continued

| Author(s) (year) and  study no. [#]^a^ | Country,  period and  population | Data and  sample size | Intervention  description | Outcome(s), measure(s)  and exposure | Study design and method | Key results and  time horizon of effects^b^ | Summary^c^ |
| --- | --- | --- | --- | --- | --- | --- | --- |
| Panel b1: Wider outcomes: Non-experimental design | | | | | | | |
| Baltagi and Yen (2016)  [69] | USA  (42 states)  1994-2005  Low-income families/TANF recipients with at most a high school degree and with children aged  <18 years | Survey data: Survey of Income and Program  Program  (SIPP),  1994-2005  n = (up to) 57,057 spells | See description provided for Acs, Phillips and Nelson (2005) [2].  Between 1996 and 2005, the number of States which implemented full (100%) initial sanctions varies between 6 and 18. | *Child outcomes*:  Child well-being (health)  1. Poor/fair health  (reported by parent)  2. Number of times parents consulted a doctor in the past 12 months  3. Nights of hospital stays in the past 12 months  *Exposure*:  Imposition of full (100%) initial benefit sanctions (state-level indicator) | Non-experimental  Two-way fixed effects regression model | Among the youngest children (aged under 6 years) of working mothers, the probability of reporting having poor or fair health increases by 3% after the implementation of a full (100%) initial sanction. No significant effect is found for the health status of older children, aged between 7 and 12 years. For these, the number of doctor consultations increases by 68% compared to the oldest children, aged between 12 and 18 years. The number of hospital stays decreases by 38% among children with non-working mother on average.  *Time horizon*: long-term | 1. 🡩  (<6 Years, working mother)  2. 🡩  (7-12 years, working mother)  3. 🡫  (children on average, non-working mother) |
| Casey et al. (2004)  [70] | USA  (6 metropolitan communities in 5 regions)  2000-2001  Lone mothers with children aged 3 years or younger/ TANF and Food Stamps recipients, being seen in hospital general clinics or emergency department | Survey data: Children’s Sentinel Nutritional Assessment  Program  (C-SNAP),  2000-2001  n =5,306 individuals | See description provided for Acs, Phillips and Nelson (2005) [2]. | *Adult outcomes*:  Health problems  1. Mental health conditions  (self-reported)  *Exposure*:  Imposition of partial (unspecified) to full (100%) benefit sanctions | Non-experimental  Logistic regression model | The imposition of benefit sanctions has no significant association with maternal depression.  *Time horizon*: short-term | 1. ○ |

Notes: ^a^ Study number and reference for each study are reported in Table A4; ^b^ Time horizon of effects is classified as: short-term (0-12 months); medium-term (13-24 months), long-term (25+ months)

^c^ Direction of effect by outcome measure number: 🡩 = significant increase (p<0.05); 🡫 = significant decrease (p<0.05); ○ = no significant change (p>0.05).

TABLE A4. Continued

| Author(s) (year) and  study no. [#]^a^ | Country,  period and  population | Data and  sample size | Intervention  description | Outcome(s), measure(s)  and exposure | Study design and method | Key results and  time horizon of effects^b^ | Summary^c^ |
| --- | --- | --- | --- | --- | --- | --- | --- |
|  |  |  |  |  |  |  |  |
| Chase-Lansdale et al. (2002)  [71] | USA  (Boston, MA, Chicago, IL, and San Antonio, TX)  1999  Low-income families/lone parents/TANF recipients | Survey data:  Children and Welfare: a three-city study,  1999  n =1,885 individuals | See description provided for Acs, Phillips and Nelson (2005) [2]. | *Child outcomes*:  Child well-being  1. Cognitive achievement  2. Behavioural problems  *Exposure*:  Imposition of partial (unspecified) to full (100%) benefit sanctions | Non-experimental:  Descriptive statistics (no statistical test provided for differences across groups) | Pre-schoolers and adolescents in sanctioned families showed problematic cognitive and behavioural outcomes. Pre-schoolers of sanctioned mothers scored 5-10 points lower than pre-schoolers of non-sanctioned mothers in cognitive test measures. For adolescents the patterns were less consistent. Higher rates of behavioural and emotional problems were observed for both pre-schoolers and adolescents of sanctioned mothers (56% and 40%, respectively).  *Time horizon*: medium term | 1. n/a  2. n/a |
|  |  |  |  |  |  |  |  |

Notes: ^a^ Study number and reference for each study are reported in Table A4; ^b^ Time horizon of effects is classified as: short-term (0-12 months); medium-term (13-24 months), long-term (25+ months)

^c^ Direction of effect by outcome measure number: 🡩 = significant increase (p<0.05); 🡫 = significant decrease (p<0.05); ○ = no significant change (p>0.05).

TABLE A4. Continued

| Author(s) (year) and  study no. [#]^a^ | Country,  period and  population | Data and  sample size | Intervention  description | Outcome(s), measure(s)  and exposure | Study design and method | Key results and  time horizon of effects^b^ | Summary^c^ |
| --- | --- | --- | --- | --- | --- | --- | --- |
|  |  |  |  |  |  |  |  |
| Chavkin, Romero and Wise (2000)  [8] | USA  1995-1998  Low-income families/lone parents | Linked  administrative-survey data:  various sources,  1995-1998  n = 50 states | See description provided for Acs, Phillips and Nelson (2005) [2]. | *Adult and child outcomes*:  Adult outcomes:  Health insurance:  1. % change in Medicaid enrolment for TANF recipients (adults and children)  2. % change in Medicaid enrolment (all adults)  3. % change in  uninsured (total population)  Child outcomes:  Health insurance:  4. % change in  uninsured (<18 years)  *Exposure*:  Imposition of a full-family (100%) benefit sanction for initial non-compliance *versus* imposition of a TANF sanction (100%) for work non-compliance to Medicaid receipt (at state level) | Non-experimental:  Ordinary Least Squares (OLS) regression model | State-level sanctions policies are not shown to be associated with health insurance status for the adult and children population.  *Time horizon*: medium-term | 1. ○  2. ○  3. ○  4. ○ |

Notes: ^a^ Study number and reference for each study are reported in Table A4; ^b^ Time horizon of effects is classified as: short-term (0-12 months); medium-term (13-24 months), long-term (25+ months)

^c^ Direction of effect by outcome measure number: 🡩 = significant increase (p<0.05); 🡫 = significant decrease (p<0.05); ○ = no significant change (p>0.05).

TABLE A4. Continued

| Author(s) (year) and  study no. [#]^a^ | Country,  period and  population | Data and  sample size | Intervention  description | Outcome(s), measure(s)  and exposure | Study design and method | Key results and  time horizon of effects^b^ | Summary^c^ |
| --- | --- | --- | --- | --- | --- | --- | --- |
|  |  |  |  |  |  |  |  |
| Cherlin et al.  (2002)  [9] | USA  (Boston, MA, Chicago, IL, and San Antonio, TX)  1999  Low-income families | Survey data:  Children and welfare: a three-city study, 1999  n = 108 individuals | See description provided for Acs, Phillips and Nelson (2005) [2]. Sanctioning rates: substantial variation at the levels of benefit office and caseworker. | *Adult outcomes*:  Material hardship:  1.Various indicators (e.g. cut back on necessities; got money from friends/family; cut back on extras; delayed/stopped paying bills)  *Exposure*:  Imposition of partial to full-family (100%) benefit sanctions (varies by state) | Non-experimental:  Descriptive statistics | Families that reported a partial or full loss of benefits (without complete restoration of these benefits) cut back on necessities and rely mainly on friends/family for support.  *Time horizon*: medium-term | 1. n/a |
| Cook et al.  (2002)  [72] | USA  (6 cities)  1998-2000  Low-income families | Survey data:  Children’s Sentinel Nutritional Assessment  Program  (C-SNAP),  1998-2000  n =2,718 individuals | See description provided for Acs, Phillips and Nelson (2005) [2]. | *Child outcomes*:  Material hardship:  1. Food insecurity  Health:  2. Hospitalised since birth, emergency department admissions  *Exposure*:  Imposition of partial to full-family (100%) benefit sanctions (varies by state) | Non-experimental:  Logistic regression | Benefit sanctions are associated with an increase in the likelihood that young children will experience food insecurity by 50% and an increase in the likelihood that they will experience hospitalisations by 30%.  *Time horizon*: short-term | 1. 🡩  2. 🡩 |

Notes: ^a^ Study number and reference for each study are reported in Table A4; ^b^ Time horizon of effects is classified as: short-term (0-12 months); medium-term (13-24 months), long-term (25+ months)

^c^ Direction of effect by outcome measure number: 🡩 = significant increase (p<0.05); 🡫 = significant decrease (p<0.05); ○ = no significant change (p>0.05).

TABLE A4. Continued

| Author(s) (year) and  study no. [#]^a^ | Country,  period and  population | Data and  sample size | Intervention  description | Outcome(s), measure(s)  and exposure | Study design and method | Key results and  time horizon of effects^b^ | Summary^c^ |
| --- | --- | --- | --- | --- | --- | --- | --- |
|  |  |  |  |  |  |  |  |
| Davis (2019)  [73] | USA  2000-2015  Low-income families | Linked administrative data: Welfare Rules Database and Office of Family Assistance and Behavioral Risk Factor Surveillance System,  2000-2015  n = 195 state-year observations;  233,716 individuals | See description provided for Acs, Phillips and Nelson (2005) [2]. | *Adult outcomes*:  Health:  1. Days of mental ill-health  *Exposure*:  Imposition of partial (33%) to full-family (100%) benefit sanctions (state-level indicator) | Non-experimental:  Fixed effects and random effects models | Compared with less-stringent state-level sanction policies, harsher state-level sanction policies are associated with worse mental health among low-educated lone mothers – the main effect size is 1.07 additional days of mental ill-health.  *Time horizon*: short-term | 1. 🡩 |
| Dunifon, Hynes and Peters (2009)  [74] | USA  1992-1999  Low-income families | Linked administrative-survey data:  Welfare Rules Database;  Survey of Income and Programme Participation (SIPP), 1992, 1993, and 1996  n= 45,847 person-years observations | See description provided for Acs, Phillips and Nelson (2005) [2].  Prior to 1996 welfare reform, states had leeway to opt out of AFDC system requirements and to develop their own welfare policies, such as: work requirements, stringent child support enforcement provisions, and rules that increased cash assistance for married-couple families. | *Child outcomes*:  Demographic outcomes:  1. Living arrangements  *Exposure*:  Imposition of partial to full-family (100%) benefit sanctions (state-level indicator) | Non-experimental:  Multinomial logistic regression with fixed effects | State-level sanctions policies are not shown to be associated with children’s living arrangements.  *Time horizon*: short-term | 1. ○ |

Notes: ^a^ Study number and reference for each study are reported in Table A4; ^b^ Time horizon of effects is classified as: short-term (0-12 months); medium-term (13-24 months), long-term (25+ months)

^c^ Direction of effect by outcome measure number: 🡩 = significant increase (p<0.05); 🡫 = significant decrease (p<0.05); ○ = no significant change (p>0.05).

TABLE A4. Continued

| Author(s) (year) and  study no. [#]^a^ | Country,  period and  population | Data and  sample size | Intervention  description | Outcome(s), measure(s)  and exposure | Study design and method | Key results and  time horizon of effects^b^ | Summary^c^ |
| --- | --- | --- | --- | --- | --- | --- | --- |
|  |  |  |  |  |  |  |  |
| Eardley (2006)  [75] | Australia  2000-2002  Unemployment Assistance (UA) benefit claimants | Survey data:  Telephone survey conducted by author, 2002  n= 1,005 individuals | There have always been sanctions within unemployment benefit system but the introduction of the ‘Mutual Obligation’ regime (1999-2001) led to a large increase in the number of breaches imposed. Employment services agencies came under contractual obligation to report non-compliance. Public campaign against rise in sanctions eventually led to their rates falling. | *Adult outcomes*:  Compliance:  1. Compliance with requirements  Material hardship:  2. Living expenses problems  3. Housing problems  4. Borrowing/debt  Health:  5. Health problems  Other:  6. Social relationships problems  7. Risk-taking behaviour  *Exposure*:  Imposition of benefit sanctions (‘breach’): A$ 384 (one breach) or A$ 3,491 (3 breaches within 2-year period) | Non-experimental:  Ordinary Least Squares (OLS) regression model | Compared with those who had had a sanction overturned, sanctions lead to a greater compliance with requirements but negative effects are nevertheless widespread, including impacts on living expenses, reliance on friends/family for support, housing problems, health impacts and risk-taking activities.  *Time horizon*: short- to medium-term. | 1. 🡩  2. 🡩  3. ○  4. 🡩  5. 🡩  6. 🡩  7. 🡩 |
| Fitzgerald and Ribar (2004)  [76] | USA  1989-2000  Low-income families/lone parents | Linked administrative-survey data:  Survey of Income and Programme Participation (SIPP),  1990, 1992, 1993, and 1996;  Welfare Rules Database  n = (up to)  654,327 person-waves | See description provided for Acs, Phillips and Nelson (2005) [2]. Prior to 1996 welfare reform, states had leeway to opt out of AFDC system requirements and to develop their own welfare policies, such as: work requirements, stringent child support enforcement provisions, and rules that increased cash assistance for married-couple families. | *Adult outcomes*:  Demographic outcomes:  1. Female household headship status  *Exposure*:  Imposition of partial to full-family (100%) benefit sanctions (state-level indicator) | Non-experimental  Logistic regression model with fixed effects and Cox proportional hazards models | State-level sanctions policies are not shown to be associated with female headship.  *Time horizon*: short-term | 1. ○ |

Notes: ^a^ Study number and reference for each study are reported in Table A4; ^b^ Time horizon of effects is classified as: short-term (0-12 months); medium-term (13-24 months), long-term (25+ months)

^c^ Direction of effect by outcome measure number: 🡩 = significant increase (p<0.05); 🡫 = significant decrease (p<0.05); ○ = no significant change (p>0.05).

TABLE A4. Continued

| Author(s) (year) and  study no. [#]^a^ | Country,  period and  population | Data and  sample size | Intervention  description | Outcome(s), measure(s)  and exposure | Study design and method | Key results and  time horizon of effects^b^ | Summary^c^ |
| --- | --- | --- | --- | --- | --- | --- | --- |
|  |  |  |  |  |  |  |  |
| Graefe, Irving and DeJong (2006)  [12] | USA  1996-2003  Low**-**income families | Survey data: Survey of Income and Program Participation (SIPP), 1996 and 2001  n = 7,062 spells; 4,999 individuals | See description provided for Acs, Phillips and Nelson (2005) [2]. | *Adult outcome****s***:  E outcomes:  1. Entry into marriage  2. Entry into cohabitation  *Exposure*:  State-level indicator:  Stringent sanction policies (temporary or permanent ineligibility) versus lenient sanction policies (partial loss of benefits) | Non-experimental:  Discrete-time competing risks models: multinomial logistic regression models | Compared with partial sanction policies, stringent sanction policies are not shown to have a significant association with transitions to marriage but are found to reduce the likelihood of transitions to cohabitation by 32%.  *Time horizon*: long-term | 1. ○  2. 🡫 |
| Hunter and Santhiveeran  (2005)  [77] | USA  1999  Low-income families who received their last TANF benefits in 1997 | Survey data:  National Survey of America's Families  (NSAF), 1999  n = 220 families | See description provided for Acs, Phillips and Nelson (2005) [2]. | *Adult outcomes*:  Material hardship:  1. Food deprivation  2. Financial hardship  3. Inability to pay rent/  utility bills  *Exposure*:  Imposition of partial to full (100%) benefit sanctions which led respondents to involuntarily leave welfare | Non-experimental:  Descriptive statistics | Compared to those who left TANF voluntarily, those who were involuntarily cut off from welfare were less unable to pay rent or other household utility bills (30% vs 43%). This difference was statistically significant at a 0.05 significance level.  *Time horizon*: short-term | 1. ○  2. ○  3. 🡫 |

Notes: ^a^ Study number and reference for each study are reported in Table A4; ^b^ Time horizon of effects is classified as: short-term (0-12 months); medium-term (13-24 months), long-term (25+ months)

^c^ Direction of effect by outcome measure number: 🡩 = significant increase (p<0.05); 🡫 = significant decrease (p<0.05); ○ = no significant change (p>0.05).

TABLE A4. Continued

| Author(s) (year) and  study no. [#]^a^ | Country,  period and  population | Data and  sample size | Intervention  description | Outcome(s), measure(s)  and exposure | Study design and method | Key results and  time horizon of effects^b^ | Summary^c^ |
| --- | --- | --- | --- | --- | --- | --- | --- |
|  |  |  |  |  |  |  |  |
| Kalil, Seefeldt and Wang  (2002)  [78] | USA  (a urban county, Michigan)  1997-1999  Lone mothers/  TANF recipients in an urban county in Michigan in 1997 | Survey data:  Women's Employment Study (WES), 1997, 1998 and 1999  n = 562 individuals | See description provided for Acs, Phillips and Nelson (2005) [2].  In Michigan, Work First is the state’s job search, training and employment programme. TANF recipients are required to participate in work-related activities for at least 20 hours (if child aged < 6 years) or 25 hours (parents with older children). Sanctions are applied mostly for non-compliance with work-related requirements although in a minority of cases sanctions were also applied for non-compliance with child-support requirements. | *Adult outcomes*:  Material hardship:  1. Utility cut-off  2. Having had at least one hardship experience  3. Expecting hardship in the next 2 months  *Exposure*:  Imposition of partial benefit sanctions only.  First stage: 25% reduction in TANF/food stamp grants; duration: until compliance or maximum 4 month, then full (100%) reduction/case closure; duration: minimum 1 month (second stage). | Non-experimental:  Logistic regression model | Those receiving a sanction were 1.66 times more likely to experience utility shutoffs and were 2.03 times more likely to be engaging in hardship-related activities. Sanctions also increased recipients’ subjective perceptions of material hardship by 1.41 times.  *Time horizon*: short-term | 1. 🡩  2. 🡩  3. 🡩 |
|  |  |  |  |  |  |  |  |

Notes: ^a^ Study number and reference for each study are reported in Table A4; ^b^ Time horizon of effects is classified as: short-term (0-12 months); medium-term (13-24 months), long-term (25+ months)

^c^ Direction of effect by outcome measure number: 🡩 = significant increase (p<0.05); 🡫 = significant decrease (p<0.05); ○ = no significant change (p>0.05).

TABLE A4. Continued

| Author(s) (year) and  study no. [#]^a^ | Country,  period and  population | Data and  sample size | Intervention  description | Outcome(s), measure(s)  and exposure | Study design and method | Key results and  time horizon of effects^b^ | Summary^c^ |
| --- | --- | --- | --- | --- | --- | --- | --- |
|  |  |  |  |  |  |  |  |
| Larson, Singh and Lewis  (2011)  [79] | USA  (Minnesota)  2005-2006  Low-income families | Linked administrative data from the Minnesota Departments of Human Services and Education  n = 19,381 children from 19,381 families (1 child randomly selected per family) | See description provided for Acs, Phillips and Nelson (2005) [2]. Sanctioning rates: in 2008 in Minnesota 5.5% of TANF recipients experienced sanctions for non-compliance with work-related requirements and 0.8% of all cases experiences six sanctions and were therefore eligible for case closure. | *Child outcomes*:  Education  1. School attendance rate  2. Enrolment disruptions  *Exposure*:  Imposition of at least one benefit sanction. Sanctions can be either partial or full-family (100%) sanctions. First instance: 10% reduction for one eligible adult or 30% if two eligible adults in the household. Second instance: increase to (additional) 30% for each month of non-compliance. After 6 instances: 100% reduction, but families can apply for Food Support. | Non-experimental:  Descriptive  analysis | Over the 24-month study period, elementary-school children from families with at least one sanction had significantly lower mean attendance ratios while the total number of disruptions to children’s school enrolment did not vary by number of sanctions received their families.  *Time horizon*: medium-term | 1. 🡫  2. ○ |

Notes: ^a^ Study number and reference for each study are reported in Table A4; ^b^ Time horizon of effects is classified as: short-term (0-12 months); medium-term (13-24 months), long-term (25+ months)

^c^ Direction of effect by outcome measure number: 🡩 = significant increase (p<0.05); 🡫 = significant decrease (p<0.05); ○ = no significant change (p>0.05).

TABLE A4. Continued

| Author(s) (year) and  study no. [#]^a^ | Country,  period and  population | Data and  sample size | Intervention  description | Outcome(s), measure(s)  and exposure | Study design and method | Key results and  time horizon of effects^b^ | Summary^c^ |
| --- | --- | --- | --- | --- | --- | --- | --- |
|  |  |  |  |  |  |  |  |
| Lee, Slack and Lewis (2004)  [23] | USA  (Illinois)  1999-2001  Female TANF recipients aged 15-64 years with a child aged < 18 years | Linked admin-survey data:  Illinois Family Study (IFS), 1999/2000, 2001 and 2002;  Admin data from Illinois Department of Human Services and the Illinois Department of Employment Security  n = 921 individuals | See description provided for Acs, Phillips and Nelson (2005) [2].  Under TANF, Illinois included both incentives and penalties. Among incentives there are generous income disregard policy, an inclusive definition of legitimate work activities (e.g. self-employment, job search, training and child care provided for a person in community service programme). Gradual sanction approach applies for non-compliance with work requirements, child support and children’s school attendance. | *Adult outcomes*:  Material hardship:  1. Rent hardship  2. Utility hardship  3. Food hardship  4. Overall perceived hardship  *Exposure*:  Imposition (I) or threat (T) of benefit sanctions. Gradual sanctions with 3 steps: (1) Partial-grant sanctions (50% reduction) until compliance; (2) Partial-grant sanctions: benefits reduced by extra 50% until compliance or for 3 months; (3) Full-grant or full-family sanction for 3 months. | Non-experimental:  Ordinary Least Squares (OLS) and logistic regression models | Those with an imposed sanction (I) were 2.23 times more likely to experience food hardship than those who did not received a sanction. Those experiencing the threat of a sanction (T) were 1.12 times more likely to experience rent hardship than those with not a threat of a sanction.  *Time horizon*: short- to medium-term | 1. ○ (I)  🡩(T)  2. ○ (I)  ○ (T)  3. 🡩 (I)  ○ (T)  4. ○ (I)  ○ (T) |

Notes: ^a^ Study number and reference for each study are reported in Table A4; ^b^ Time horizon of effects is classified as: short-term (0-12 months); medium-term (13-24 months), long-term (25+ months)

^c^ Direction of effect by outcome measure number: 🡩 = significant increase (p<0.05); 🡫 = significant decrease (p<0.05); ○ = no significant change (p>0.05).

TABLE A4. Continued

| Author(s) (year) and  study no. [#]^a^ | Country,  period and  population | Data and  sample size | Intervention  description | Outcome(s), measure(s)  and exposure | Study design and method | Key results and  time horizon of effects^b^ | Summary^c^ |
| --- | --- | --- | --- | --- | --- | --- | --- |
|  |  |  |  |  |  |  |  |
| Lindhorst and  Mancoske (2006)  [26] | USA  (Louisiana)  1998-2001  Lone parents, aged 18 years/  TANF recipients | Survey data: Panel Study of Welfare Recipients, 1998-2001  n = (up to) 348 individuals | See description provided for Acs, Phillips and Nelson (2005) [2].  In Louisiana, the Family Independence Assistance Program (FITAP) was created as part of TANF. Recipients are required to participate in approved work activities for at least 20 hours (if child aged one year or older). A time limit policy applies to welfare recipients after 24 months of benefit payments and families must wait two years before re-applying. Temporary exemptions are given to people because of physical health problems of the mother or care of a child with a disability. | *Adult and child outcomes*:  Adult and child outcomes:  Material hardship  1. % insufficient food  2. % housing problems  3. % unable to obtain medical care for parent  4. % unable to obtain medical care for child  5. % needed, but did not receive Medicaid  Adult outcomes:  Health:  6. % current major depression  7. number of days of poor physical health  *Exposure*:  Imposition of partial or full-family (100%) benefit sanctions | Non-experimental:  Descriptive analysis: Analysis of Variance (ANOVA) | There were significant differences between those sanctioned and other welfare categories in terms of the percentage of those who were not able to receive Medicaid.  *Time horizon*: short- to medium-term | 1. ○  2. ○  3. ○  4. ○  5. 🡩  6. ○  7. ○ |

Notes: ^a^ Study number and reference for each study are reported in Table A4; ^b^ Time horizon of effects is classified as: short-term (0-12 months); medium-term (13-24 months), long-term (25+ months)

^c^ Direction of effect by outcome measure number: 🡩 = significant increase (p<0.05); 🡫 = significant decrease (p<0.05); ○ = no significant change (p>0.05).

TABLE A4. Continued

| Author(s) (year) and  study no. [#]^a^ | Country,  period and  population | Data and  sample size | Intervention  description | Outcome(s), measure(s)  and exposure | Study design and method | Key results and  time horizon of effects^b^ | Summary^c^ |
| --- | --- | --- | --- | --- | --- | --- | --- |
|  |  |  |  |  |  |  |  |
| Lindhorst, Mancoske, and Kemp (2000)  [27] | USA  (Southern metropolitan region)  1998  Low-income families/lone parents | Survey data: authors’ telephone survey, 1998  n = 347 individuals | See description provided for Acs, Phillips and Nelson (2005) [2]. | *Adult and child outcomes*:  Material hardship  1. Total number of problems  2. Respondents: went without food; family had other problems; heat/  utilities cut off.  3. Health insurance status:  could not afford medical care/medications.  4. Rent-related issues; homelessness/move in with others; phone disconnected; use of food bank/soup kitchen;  5. Child outcomes: Child(ren): had to change school; had to live away from caregiver; spent time in foster care  *Exposure*:  Imposition of full-family (100%) benefit sanctions | Non-experimental:  Descriptive analysis | When compared with those who left benefits voluntarily, those who were sanctioned reported significantly higher levels of: overall number of problems they experienced; having unmet medical needs; going without food; and having their utilities turned off.  *Time horizon*: medium-term | 1. 🡩  2. 🡩  3. 🡩  4. ○  5. ○ |

Notes: ^a^ Study number and reference for each study are reported in Table A4; ^b^ Time horizon of effects is classified as: short-term (0-12 months); medium-term (13-24 months), long-term (25+ months)

^c^ Direction of effect by outcome measure number: 🡩 = significant increase (p<0.05); 🡫 = significant decrease (p<0.05); ○ = no significant change (p>0.05).

TABLE A4. Continued

| Author(s) (year) and  study no. [#]^a^ | Country,  period and  population | Data and  sample size | Intervention  description | Outcome(s), measure(s)  and exposure | Study design and method | Key results and  time horizon of effects^b^ | Summary^c^ |
| --- | --- | --- | --- | --- | --- | --- | --- |
| Livermore  et al. (2015)  [80] | USA  (Louisiana)  2003-2007  Lone parents/TANF recipients, former participants in STEP programme | Linked administrative-survey data:  Administrative data from Louisiana Department of Children and Family Sources (DCFS) and Louisiana Workforce Commission (LWC), 2003-2006;  Survey data collected by authors, 2007  n = 459 individuals | See description provided for Acs, Phillips and Nelson (2005) [2].  The Strategies to Empower People (STEP) programme is the work programme associated with Louisiana’s TANF cash assistance. Participants must be aged < 60 years and must not have a permanent disability or be caring for a family member with a disability. Participants who are pregnant or with a child aged <1 year are required to receive parenting skills training while others receive job preparation training. | *Adult and child outcomes*:  Material hardship:  1. Experiencing material hardship (various indicators: food deprivation, utility disconnection, homelessness/eviction)  *Exposure*:  Imposition of 100% benefit sanction (welfare case closure for 3 months). Sanctions are applied for non-compliance with requirements related to work, child support and immunisation. | Non-experimental:  Logistic regression model | Among those who left welfare and were sanctioned while on welfare were 80% more likely to experience material hardships, such as food deprivation, utility disconnection and homelessness.  *Time horizon*: medium- to long-term | 1. 🡩 |
| Lohman et al.  (2004)  [81] | USA  (Boston, MA, Chicago, IL, and San Antonio, TX)  1999  Low-income children (aged 2-4 years and 10-14 years) and their families from low-income urban neighbourhoods | Survey data:  Welfare, Children, and Families: Three-City Study,  1999  n = (up-to) 1,885 children | See description provided for Acs, Phillips and Nelson (2005) [2]. | *Child outcomes*:  Child well-being:  1. Cognitive achievement  2. Behavioural problems  *Exposure*:  Imposition of partial or full (100%) benefit sanctions | Non-experimental:  Descriptive analysis: dummy or no-constant regression | Pre-school children of sanctioned mothers had lower scores of 9-10 points compared to children of non-sanctioned mothers in cognitive achievement tests. In terms of behavioural problems, pre-school children also displayed a scoring in the range of concern which was twice or more as higher than children in other groups. Adolescent of sanctioned welfare leavers revealed the highest rates of behavioural problems although differences were not statistically significant.  *Time horizon*: medium-term | 1. 🡫  2. 🡩  (pre-school children only) |

Notes: ^a^ Study number and reference for each study are reported in Table A4; ^b^ Time horizon of effects is classified as: short-term (0-12 months); medium-term (13-24 months), long-term (25+ months)

^c^ Direction of effect by outcome measure number: 🡩 = significant increase (p<0.05); 🡫 = significant decrease (p<0.05); ○ = no significant change (p>0.05).

TABLE A4. Continued

| Author(s) (year) and  study no. [#]^a^ | Country,  period and  population | Data and  sample size | Intervention  description | Outcome(s), measure(s)  and exposure | Study design and method | Key results and  time horizon of effects^b^ | Summary^c^ |
| --- | --- | --- | --- | --- | --- | --- | --- |
|  |  |  |  |  |  |  |  |
| Loopstra et al. (2018)  [82] | UK  (Great Britain)  2012-2015  Unemployment Insurance (UI) benefit claimants, receiving Job Seekers’ Allowance (JSA) | Linked administrative data (area-level): various sources,  2012-2015  n = 3,041 local authority-quarters;  259 local authorities | Starting in 2010, three reforms were implemented: (1) policy interventions were introduced to move people deemed able to work from Incapacity Benefits/Employment Support Allowance or Income Support onto Jobseeker’s Allowance (JSA); (2) conditions to obtain JSA became more stringent: a mandatory Work Programme and a Claimant Commitment introduce contractual obligations for JSA claimants; (3) introduction of more severe sanctions for non-compliance with work-related activities set in Claimant Commitment | *Adult outcomes*:  Material hardship:  1. Number of adults fed by food banks  *Exposure*:  Number of sanctions imposed to JSA benefit claimants (at local-authority level) | Non-experimental:  Fixed effects regression models | An increase of 10 additional sanctions per 100,000 JSA benefit claimants is associated with an increase of 3.36 adults per 100,000 being fed by food banks.  *Time horizon*: long-term | 1. 🡩 |

Notes: ^a^ Study number and reference for each study are reported in Table A4; ^b^ Time horizon of effects is classified as: short-term (0-12 months); medium-term (13-24 months), long-term (25+ months)

^c^ Direction of effect by outcome measure number: 🡩 = significant increase (p<0.05); 🡫 = significant decrease (p<0.05); ○ = no significant change (p>0.05).

TABLE A4. Continued

| Author(s) (year) and  study no. [#]^a^ | Country,  period and  population | Data and  sample size | Intervention  description | Outcome(s), measure(s)  and exposure | Study design and method | Key results and  time horizon of effects^b^ | Summary^c^ |
| --- | --- | --- | --- | --- | --- | --- | --- |
|  |  |  |  |  |  |  |  |
| Loopstra et al. (2015)  [83} | UK  (Great Britain)  2009-2014  Unemployment Insurance (UI) benefit claimants with long-term physical and mental disability: Job Seekers’ Allowance (JSA) | Linked administrative and survey data (area-level):  various sources, 2009-2014  n = 1,071 local authority/ financial years observations;  346 local authorities | See description provided for Loopstra et al. (2018) [82]. | *Adult outcomes*:  Material hardship:  1. Food bank opening  2. % change in food bank use per capita  *Exposure*:  Rate of adverse sanction imposed per JSA benefit claimant (at local-authority level) | Non-experimental:  Logistic regression and Ordinary Least Squares (OLS) regression models | While each 1% increase in the rate of benefit sanctions is not significantly associated with the probability of a food bank opening, it is significantly associated with an increase of 0.09 percentage point change in food bank use per capita.  *Time horizon*: short-term | 1. ○  2. 🡩 |
| Moffitt (2000)  [29] | USA  (Boston, MA, Chicago, IL, and San Antonio, TX)  1996-1999  TANF leavers living in low- and moderate-income neighbourhoods | Survey data:  Three-City Study, 1999  n = 339 individuals | See description provided for Acs, Phillips and Nelson (2005) [2].  Note that around 2/3 of the sample of TANF leavers receive other benefits (e.g. Medicaid, subsidised housing, school meals) while more than 1/3 receive food stamps. | *Adult outcomes*:  Health insurance:  1. Ever covered by health insurance  Material hardship:  2. Poverty rate (%)  *Adult and child outcomes*:  Health insurance:  3. % in receipt of Medicaid  *Exposure*:  Imposition of full-family (100%) benefit sanction | Non-experimental:  Descriptive analysis | Among TANF leavers, those who were sanctioned had lower health insurance coverage rates (2 percentage points) two years after interview date. Sanctioned TANF leavers also had lower Medicaid coverage (1 percentage point) and higher rates poverty at the household level (18 percentage points)  *Time horizon*: medium-term | 1. 🡫  2. 🡩  3. 🡫 |

Notes: ^a^ Study number and reference for each study are reported in Table A4; ^b^ Time horizon of effects is classified as: short-term (0-12 months); medium-term (13-24 months), long-term (25+ months)

^c^ Direction of effect by outcome measure number: 🡩 = significant increase (p<0.05); 🡫 = significant decrease (p<0.05); ○ = no significant change (p>0.05).

TABLE A4. Continued

| Author(s) (year) and  study no. [#]^a^ | Country,  period and  population | Data and  sample size | Intervention  description | Outcome(s), measure(s)  and exposure | Study design and method | Key results and  time horizon of effects^b^ | Summary^c^ |
| --- | --- | --- | --- | --- | --- | --- | --- |
|  |  |  |  |  |  |  |  |
| Oggins and Fleming (2001)  [32] | USA  (New York state)  1997-1999  Low-income families | Survey data: conducted by authors, 1997 and 1999  n = 118 individuals | See description provided for Acs, Phillips and Nelson (2005) [2]. Sanction rates: There is an incentive for states to sanction claimants in order to get them off rolls, as this improves the work participation formula to maintain TANF block grant. | *Adult and child outcomes*:  Material hardship:  1. Harder to pay for bills, rent and adult health care in past 6 months; moved due to lack of rent; child changed schools; no phone.  2. Lacked food in past month  *Exposure*:  Imposition of 100% benefit sanctions; average duration: 106 days | Non-experimental:  Logistic regression models | After being sanctioned, respondents noted greater hardship paying for adult health care, bills and rent; a greater likelihood of moving due to lack of rent; a greater likelihood of children having to move schools; and a greater likelihood of having no phone.  *Time horizon*: short- to medium-term | 1. 🡩  2. ○ |
| Ovwigho, Leavitt and Born (2003)  [84] | USA  (Maryland)  1996-2002  Low-income families; TANF/TCA recipients who left welfare benefits between 1996 and 2001 | Linked administrative data from Maryland’s Life After Welfare study: various databases  n = 17,441 children;  8,900 households | See description provided for Acs, Phillips and Nelson (2005) [2].  The Temporary Cash Assistance (TCA) programme is the TANF programme in Maryland. Child-only cases, where the adult caregiver is not part of the assistance unit, are an increasing proportions of the TANF caseload as the number of traditional lone parents has declined. | *Child outcomes*:  Child maltreatment:  1. Report of child maltreatment  *Exposure*:  Involuntary exit (case closure) from benefits due to sanctions. Sanctions can be either partal or full-family (100%) sanctions. Sanctions are applied for non-compliance with work or child-support requirements. | Non-experimental:  Discrete-time hazard models: logistic regression models | Families exiting benefits due to sanctions three years after the reform have significantly higher child maltreatment rates (90%) than families exiting benefits for other reason (e.g. voluntary exits or exits due to work/income).  *Time horizon*: short-term | 1. 🡩 |

Notes: ^a^ Study number and reference for each study are reported in Table A4; ^b^ Time horizon of effects is classified as: short-term (0-12 months); medium-term (13-24 months), long-term (25+ months)

^c^ Direction of effect by outcome measure number: 🡩 = significant increase (p<0.05); 🡫 = significant decrease (p<0.05); ○ = no significant change (p>0.05).

TABLE A4. Continued

| Author(s) (year) and  study no. [#]^a^ | Country,  period and  population | Data and  sample size | Intervention  description | Outcome(s), measure(s)  and exposure | Study design and method | Key results and  time horizon of effects^b^ | Summary^c^ |
| --- | --- | --- | --- | --- | --- | --- | --- |
|  |  |  |  |  |  |  |  |
| Paxson and Waldfogel (2003)  [85] | USA  1990-1998  Low-income families/TANF recipients | Linked administrative- survey data (state-level):  various sources,  1990-1998  n = 389 state-year observations;  49 states | See description provided for Acs, Phillips and Nelson (2005) [2]. | *Child outcomes*:  Child maltreatment  1. Reports of child maltreatment  2. Substantiated cases of maltreatment  3. Cases of abuse/neglect  4. Children in foster care  *Exposure*:  First imposition of a full-family (100%) benefit sanction (state-level indicator) | Non-experimental:  Ordinary Least Square (OLS) regression models with fixed effects | Severe sanctions are positively and significantly associated with the number of substantiated (or confirmed) cases of child maltreatment and the number of children placed in foster care, but there is no significant association with other measures of maltreatment (number of reports and cases of abuse or neglect).  *Time horizon*: short- to medium-term | 1. ○  2. 🡩  3. ○  4. 🡩 |
| Pingle (2005)  [35] | USA  1997-2000  Lone parents aged 18-30 years with children aged <5 years | Linked administrative-survey data:  Welfare Rules Database;  Current Population Survey (CPS) March Annual Demographic Supplement, 1997-2000  n = 4,643 individuals | See description provided for Acs, Phillips and Nelson (2005) [2].  Before the 1996 welfare reform, as part of AFDC, states were allowed to pay lower welfare benefits to single mothers who lived with their parents (shelter allowance); this was applied to women who lived with others (in general) or received public housing or any form of housing assistance. After 1996 welfare reform, many states retained these benefit reductions or similar policies. | *Adult outcomes*:  Demographic outcomes:  1.Whether living with parents and in/not in employment  *Exposure*:  Imposition of a severe initial benefit sanction versus permanent benefit sanction (state-level indicator) | Non-experimental:  Multi-level discrete-time competing risks models: multinomial logistic regression models with random effects | Both the severity of the initial sanction and the imposition of permanent sanctions are not significantly associated with the likelihood that a single mother lives with her parents regardless of her employment status.  *Time horizon*: long-term | 1. ○ |

Notes: ^a^ Study number and reference for each study are reported in Table A4; ^b^ Time horizon of effects is classified as: short-term (0-12 months); medium-term (13-24 months), long-term (25+ months)

^c^ Direction of effect by outcome measure number: 🡩 = significant increase (p<0.05); 🡫 = significant decrease (p<0.05); ○ = no significant change (p>0.05).

TABLE A4. Continued

| Author(s) (year) and  study no. [#]^a^ | Country,  period and  population | Data and  sample size | Intervention  description | Outcome(s), measure(s)  and exposure | Study design and method | Key results and  time horizon of effects^b^ | Summary^c^ |
| --- | --- | --- | --- | --- | --- | --- | --- |
|  |  |  |  |  |  |  |  |
| Reeves and Loopstra  (2017)  [86] | UK  (Great Britain)  2009/10-2014/15  Unemployment Insurance (UI) benefit claimants of working age, receiving Job Seekers’ Allowance (JSA) | Linked survey-administrative data (area-level): various sources,  2008/09-2014/15  n = 1,205 local authority-years observations;  175 local authorities | See description provided for Loopstra et al. [82]. | *Adult outcomes*:  Vulnerable status  1. Persons with disability  2. Lone parents  *Exposure*:  Proportion of JSA benefit claimants who received an adverse benefit sanction (at local-authority level) | Non-experimental:  Ordinary Least Square (OLS) regression with fixed effects | There is significant and positive association between sanctioning rates and the proportion of JSA claimants who report a disability or being a lone parent.  *Time horizon*: long-term | 1. 🡩  2. 🡩 |
| Reichman, Teitler and Curtis (2005)  [87] | USA  1998-2003  Low-income families | Survey data: Fragile Families and Child Wellbeing Survey,  1998-2000  n = 821 individuals | See description provided for Acs, Phillips and Nelson (2005) [2]. | *Adult and child outcomes*:  Material hardship:  1. Maternal/child hunger, utility shutoffs, material hardship, moved in with family/ friends;  2. Homelessness/eviction, lack of medical care, received financial support from family/ friends,  Mother’s health:  3. Depression/anxiety;  4. Poor self-reported physical health;  Child’s health:  5. Poor physical health (reported by mother)  *Exposure*:  Imposition of partial to full-family (100%) benefit sanctions (varies by state) | Non-experimental:  Logistic regression models with fixed effects | Banefit sanctions are associated with an increased likelihood of hunger by 63%, utility shutoffs by 76%, material hardship by 85%, relying on others for housing by 75% and poor maternal physical health by 66%.  *Time horizon*: short-term | 1. 🡩  2. ○  3. ○  4. 🡩  5. ○ |

Notes: ^a^ Study number and reference for each study are reported in Table A4; ^b^ Time horizon of effects is classified as: short-term (0-12 months); medium-term (13-24 months), long-term (25+ months)

^c^ Direction of effect by outcome measure number: 🡩 = significant increase (p<0.05); 🡫 = significant decrease (p<0.05); ○ = no significant change (p>0.05).

TABLE A4. Continued

| Author(s) (year) and  study no. [#]^a^ | Country,  period and  population | Data and  sample size | Intervention  description | Outcome(s), measure(s)  and exposure | Study design and method | Key results and  time horizon of effects^b^ | Summary^c^ |
| --- | --- | --- | --- | --- | --- | --- | --- |
|  |  |  |  |  |  |  |  |
| Rodgers, Payne and Chervachidze (2006)  [88] | USA  1997-2003  Low-income families/TANF recipients | Various sources, 1997-2003  n = 50 states | See description provided for Acs, Phillips and Nelson (2005) [2]. | *Adult outcomes*:  Material hardship:  1. % change in poverty rate (1997-2003)  *Exposure*:  Imposition of weak, moderate and strong benefit sanctions  (state-level indicator) | Non-experimental:  Ordinary Least Squares (OLS) regression models | Sanction policies are associated with a 6.6% decrease in the percentage change in poverty rates  *Time horizon*: short-term | 1. 🡫 |
| Ryan, Manlove and Hofferth (2006)  [89] | USA  1989-1996  Lone mothers/  AFDC recipients aged under 40 years with a child aged under 18 years | Survey data:  Panel Study of Income Dynamics (PSID),  1989-1996  n = 26,782 person-months observations;  458 individuals | See description provided for Acs, Phillips and Nelson (2005) [2]. | *Adult outcomes*:  Demographic outcomes:  1. Having a non-marital subsequent birth  *Exposure*:  Imposition of partial to full (100%) benefit sanctions  (at state-level) for failure to comply with work or other requirements. Severe sanction policies include loss of the entire benefit for any failure to comply. | Non-experimental:  Discrete-time hazard models: logistic regression models with state fixed effects | Severe sanctions are not associated with non-marital childbearing  *Time horizon*: long-term | 1. ○ |

Notes: ^a^ Study number and reference for each study are reported in Table A4; ^b^ Time horizon of effects is classified as: short-term (0-12 months); medium-term (13-24 months), long-term (25+ months)

^c^ Direction of effect by outcome measure number: 🡩 = significant increase (p<0.05); 🡫 = significant decrease (p<0.05); ○ = no significant change (p>0.05).

TABLE A4. Continued

| Author(s) (year) and  study no. [#]^a^ | Country,  period and  population | Data and  sample size | Intervention  description | Outcome(s), measure(s)  and exposure | Study design and method | Key results and  time horizon of effects^b^ | Summary^c^ |
| --- | --- | --- | --- | --- | --- | --- | --- |
|  |  |  |  |  |  |  |  |
| Slack, Lee and Berger (2007)  [90] | USA  (9 selected counties, Illinois)  1999-2003  Lone parents/ TANF recipients | Linked administrative-survey data:  Admin data from various sources, 1999-2003;  Illinois Family Study (IFS)  1999-2000  n = 14,640 person-quarters observations; 1,260 individuals | See description provided for Acs, Phillips and Nelson (2005) [2].  TANF programme in Illinois involves a mixture of work incentives and penalties for non-compliance with welfare requirements. Incentives include generous income disregards, a stopped clock policy (where the TANF 60-month lifetime limit stops whenever recipients work a minimum of hours) and a broad definition of work activities. Disincentives include a family cap which was discontinued in 2004 and sanctions involving additional reductions in food stamps and Medicaid coverage for the head of the household. | *Child outcomes*:  Child maltreatment:  1. Reports of child neglect  2. Reports of child abuse  3. Indicated reports of maltreatment  *Exposure*:  Imposition of partial (50%) benefit sanctions for first and second instances of non-compliance with welfare requirements to full-family (100%) benefit sanctions for a third episode of non-compliance. Sanctions can be supplemented by other sources of income such as earnings or food stamps. | Non-experimental:  Cox proportional hazards models and discrete-time hazard models: logistic regression models with fixed effects | Those receiving a sanction without any income supplement have a risk of receiving an investigation following a report for child neglect which is 2.02 times higher than non-sanctioned recipients. No significant effects were found for reports of child abuse and indicated reports for child maltreatment following investigation from the child protection system.  *Time horizon*: n/a | 1. 🡩  2. ○  3. ○ |

Notes: ^a^ Study number and reference for each study are reported in Table A4; ^b^ Time horizon of effects is classified as: short-term (0-12 months); medium-term (13-24 months), long-term (25+ months)

^c^ Direction of effect by outcome measure number: 🡩 = significant increase (p<0.05); 🡫 = significant decrease (p<0.05); ○ = no significant change (p>0.05).

TABLE A4. Continued

| Author(s) (year) and  study no. [#]^a^ | Country,  period and  population | Data and  sample size | Intervention  description | Outcome(s), measure(s)  and exposure | Study design and method | Key results and  time horizon of effects^b^ | Summary^c^ |
| --- | --- | --- | --- | --- | --- | --- | --- |
| Panel b2: Wider outcomes: Quasi-experimental design | | | | | | | |
| Acs and Nelson (2004)  [91] | USA  (13 states)  1997-1999  Low-income families | Survey data: National Surveys of America’s Families (NSAF)  1997 and 1999  n = n/a | See description provided for Acs, Phillips and Nelson (2005) [2]. | *Adult and child outcomes*:  Demographic outcomes  1. Single-mother household  2. Two-parent household  3. Children not living with parents  *Exposure*:  State-level indicator: imposition of full-family (100%) benefit sanctions | Quasi-experimental:  Difference-in-difference-in-differences (DiDiD) | State-level sanctions policies are found to have no clear consistent association with the living arrangements of low-income families with children.  *Time horizon*: short-term | 1. ○  2. ○  3. ○ |
| Machin and Marie (2006)  [92] | United Kingdom  (England and Wales)  1995-1996  Unemployment Insurance (Job Seekers’ Allowance (JSA)) benefit claimants of working age | Administrative data:  Joint Unemployment and Vacancies On-line System (JUVOS) database and Home Office crime data, 1995-1996  n = 225 area-quarters observations;  45 police-force areas | In October 1996, the Jobseeker’s Allowance (JSA) programme was created, by replacing pre-exiting benefits such as Unemployment Benefit (UB) and Income Support (IS). The new stringent unemployment benefit regime brought about major changes to the entitlement of unemployment benefits and the introduction of sanctions through the tightening of the monitoring of job-search activities. The duration of means-tested contributory benefits was also reduced from 12 to 6 months. | *Adult outcomes*:  Crime  1. Change in property crime rates  2. Change in violent crime rates  *Exposure*:  Outflow from the JSA claimant count due to claim withdrawal or failure to sign in at the job centre (area-level indicator). This indicator is deemed to be linked to the increase in the imposition of sanctions related to JSA claims. | Quasi-experimental:  Difference-in-differences (DiD) | A greater proportion of claimants affected by JSA introduction and hence moving off the unemployment register, due to an increase in benefit cuts and sanctions, has a significant effect on property crime rates but no effect on violent crime rates.  *Time horizon*: short-term | 1. 🡩  2. ○ |

Notes: ^a^ Study number and reference for each study are reported in Table A4; ^b^ Time horizon of effects is classified as: short-term (0-12 months); medium-term (13-24 months), long-term (25+ months)

^c^ Direction of effect by outcome measure number: 🡩 = significant increase (p<0.05); 🡫 = significant decrease (p<0.05); ○ = no significant change (p>0.05).

TABLE A4. Continued

| Author(s) (year) and  study no. [#]^a^ | Country,  period and  population | Data and  sample size | Intervention  description | Outcome(s), measure(s)  and exposure | Study design and method | Key results and  time horizon of effects^b^ | Summary^c^ |
| --- | --- | --- | --- | --- | --- | --- | --- |
|  |  |  |  |  |  |  |  |
| Wang  (2015)  [93] | USA  2004-2011  Low-income families/TANF recipients with children aged 0-17 years | Survey data:  Survey of Income and Program Participation  (SIPP), 2004 and 2008 panels  n = 4,163 families | See description provided for Acs, Phillips and Nelson (2005) [2]. | *Child outcomes*:  Child well-being:  1. Cognitive stimulation provided by the family  2. Family’s interactions  3. Family’s stress  4. Educational outcomes  *Exposure*:  Imposition of lenient benefit sanctions (no sanction, only sanctions on adult portion of TANF grant, partial sanctions) versus strict benefit sanctions (full-family (100%) sanctions) | Quasi-experimental:  Difference-in-differences (DiD) and  Propensity Score Matching (PSM) | Strict work sanction policies are not shown to significantly affect child well-being.  *Time horizon*: medium-term | 1. ○  2. ○  3. ○  4. ○ |

Notes: ^a^ Study number and reference for each study are reported in Table A4; ^b^ Time horizon of effects is classified as: short-term (0-12 months); medium-term (13-24 months), long-term (25+ months)

^c^ Direction of effect by outcome measure number: 🡩 = significant increase (p<0.05); 🡫 = significant decrease (p<0.05); ○ = no significant change (p>0.05).

TABLE A4. Continued

| Author(s) (year) and  study no. [#]^a^ | Country,  period and  population | Data and  sample size | Intervention  description | Outcome(s), measure(s)  and exposure | Study design and method | Key results and  time horizon of effects^b^ | Summary^c^ |
| --- | --- | --- | --- | --- | --- | --- | --- |
|  |  |  |  |  |  |  |  |
| Panel b3: Wider outcomes: Experimental design | | | | | | | |
| Fein and Lee (2003)  [94] | USA  (Delaware)  1995-1999  Lone parents/  AFDC/TANF recipients participating in A Better Chance Welfare Reform Program (ABC) | Linked administrative data  (various sources)  n = 3,959 families | See description provided for Acs, Phillips and Nelson (2005) [2].  A Better Chance Welfare Reform Program (ABC) is Delaware’s mandatory programme, designed to encourage desired employment and parenting behaviours among welfare recipients  Incentives include generous disregards for income from paid employment and child support and a wide health insurance and childcare coverage. Participants are required to participate in work-related activities for the first 2 years, a period that can be extended for another 2 years subject to workfare job participation. Welfare recipients were randomly assigned to a treatment group (subject to ABC policies) and a control group (subject to traditional AFDC rules). | *Child outcomes*:  Child maltreatment  1. Report of child maltreatment  2. Substantiated maltreatment (any maltreatment, physical/emotional abuse, sexual abuse, neglect)  3. Foster-care placement  *Exposure*:  Threat of partial to full (100%) benefit sanctions.  First non-compliance: 1/3 reduction for 2 months;  Second non-compliance: 2/3 reduction for 2 months;  Third non-compliance: permanent case closure. | Experimental:  Random assignment | The threat of benefit sanctions increases the rates of substantiated reports for child neglect by more than 50 percent during the first and third year after random assignment.  *Time horizon*: long-term | 1. ○  2. 🡩  3. ○ |

Notes: ^a^ Study number and reference for each study are reported in Table A4; ^b^ Time horizon of effects is classified as: short-term (0-12 months); medium-term (13-24 months), long-term (25+ months)

^c^ Direction of effect by outcome measure number: 🡩 = significant increase (p<0.05); 🡫 = significant decrease (p<0.05); ○ = no significant change (p>0.05).

TABLE A5. References of the studies included in the analytical sample by study number

| Study no. [#] | Reference |
| --- | --- |
|  |  |
| [1] | Abbring, J. H., van den Berg, G. and van Ours, J. C. (2005), ‘The effect of unemployment insurance sanctions on the transition rate from unemployment to employment’, *Economic Journal,* 115, 602-630. |
| [2] | Acs, G., Phillips, K. R. and Nelson, S. (2005), ‘The road not taken? Changes in welfare entry during the 1990s’, *Social Science Quarterly,* 86, s1, 1060-1079. |
| [3] | Ahmad, N., Svarer, M. and Naveed, A. (2019), ‘The effect of Active Labour Market Programmes and benefit sanctions on reducing unemployment duration’, *Journal of Labor Research*, 40, 202-229. |
| [4] | Arni P. and Schiprowski A. (2015), ‘The effects of binding and non-binding job search requirements*’,* IZA Discussion Paper no. 8951, Bonn: Institute for the Study of Labor (IZA), <https://www.iza.org/publications/dp/8951/the-effects-of-binding-and-non-binding-job-search-requirements> [accessed 01.12.2019]. |
| [5] | Arni P., Lalive R. and Van Ours J. C. (2013), ‘How effective are unemployment benefit sanctions? Looking beyond unemployment exit’, *Journal of Applied Econometrics*, 28, 7, 1153-78. |
| [6] | Born, C. E., Ovwigho, P. C. and Cordero, M. L. (2002), ‘Returns to welfare under welfare reform: Early patterns and their implications’, *Administration in Social Work*, 26, 3, 53-69. |
| [7] | Busk, H. (2016), ‘Sanctions and the exit from unemployment in two different benefit schemes’, *Labour Economics*, 42, 159-176. |
| [8] | Chavkin, W., Romero, D. and Wise, P. H. (2000), ‘State welfare reform policies and declines in health insurance’, *American Journal of Public Health*, 90, 6, 900-908. |
| [9] | Cherlin, A. J., Bogen, K., Quane, J. M. and Burton, L. (2002), ‘Operating within the rules: welfare recipients' experiences with sanctions and case closings’, *Social Service Review*, 76, 3, 387-405. |
| [10] | Diop-Christensen, A. (2015), ‘Is ‘making work pay’ effective for the ‘unemployable’? The impact of benefit sanctions on social assistance recipients in Denmark’, *Journal of European Social Policy*, 25, 2, 210-224. |
| [11] | Farrell, M., Rich, S., Turner, L., Seith, D. and Bloom, D. (2008), ‘Welfare time limits: an update on state policies, implementation, and effects on families’, New York: Manpower Demonstration Research Corporation (MDRC), <https://www.mdrc.org/publication/update-state-welfare-time-limit-policies-and-their-effects-families> [accessed 01.12.2020]. |
| [12] | Graefe, D. R., Irving, S. K. and De Jong, G. F. (2006), ‘Getting married and exiting welfare: the role of two-parent TANF eligibility rules’, paper presented at 2007 Population Association of America, <https://paa2007.princeton.edu/abstracts/7121> [accessed 01.12.2020]. |
| [13] | Hillmann, K. and Hohenleitner, I. (2015), ‘Impact of welfare sanctions on employment entry and exit from labor force: Evidence from German survey data’, HWWI  Research Paper, No. 129, Hamburg: Hamburg Institute of International Economics (HWWI), <https://www.hwwi.org/fileadmin/hwwi/Publikationen/Research/Paper/Research_Paper_168.pdf> [accessed 01.12.2020]. |
| [14] | Hofferth, S. L., Stanhope, S. and Harris, K. M. (2002), ‘Exiting welfare in the 1990s: did public policy influence recipients' behavior?’, *Population Research & Policy Review*, 21, 5, 433-472. |
| [15] | Hofferth, S. L., Stanhope, S. and Harris, K. (2005), ‘Remaining off welfare in the 1990s: the influence of public policy and economic conditions’, *Social Science Research*, 34, 2, 426-453. |

TABLE A5. Continued

| Study no. [#] | Reference |
| --- | --- |
|  |  |
| [16] | Hofmann, B. (2012), ‘Short- and long-term ex-post effects of unemployment insurance sanctions: Evidence from West Germany’, *Jahrbücher fur Nationalökonomie und Statistik*, 232, 1, 31-60. |
| [17] | Hohenleitner, I. and Hillmann, K. (2019a), ‘Impact of welfare sanctions on employment and benefit receipt: considering top-up benefits and indirect sanctions’, HWWI  Research Paper, No. 189, Hamburg, Germany: Hamburg Institute of International Economics (HWWI), <http://epub.sub.uni-hamburg.de/epub/volltexte/2019/94682/pdf/HWWI_ResearchPaper_189.pdf> [accessed 01.12.2020]. |
| [18] | Hohenleitner, I. and Hillmann, K. (2019b), ‘Impact of welfare sanctions on the quality of subsequent employment: wages, incomes, and employment stability’, HWWI  Research Paper, No. 190, Hamburg, Germany: Hamburg Institute of International Economics (HWWI), <https://www.econbiz.de/Record/impact-of-welfare-sanctions-on-the-quality-of-subsequent-employment-wages-incomes-and-employment-stability-hohenleitner-ingrid/10011985034> [accessed 01.12.2020]. |
| [19] | Irving, S. K. (2008), ‘State welfare rules, TANF exits, and geographic context: does place matter?’, *Rural Sociology*, 73, 4, 605-630. |
| [20] | Kim, J. (2010), ‘Welfare-to-work programs and the dynamics of TANF use’, *Journal of Family and Economic Issues*, 31, 198-2011. |
| [21] | Koning, P. (2015), ‘Making work pay for the indebted? Assessing the effects of debt services on welfare recipients’, *Labour Economics*, 34, 152-161. |
| [22] | Lalive, R., van Ours, J. C. and Zweimüller, J. (2005), ‘The effect of benefit sanctions on the duration of unemployment’, *Journal of the European Economic Association*, 3, 6, 1386-1417. |
| [23] | Lee, B. J., Slack, K. S. and Lewis, D. A. (2004) ‘Are welfare sanctions working as intended? Welfare receipts, work activity, and material hardship among TANF-recipient families’, *Social Service Review*, 78, 3, 370-403. |
| [24] | Lee, H. J. and Tomohara, A. (2007), ‘How did public assistance reforms affect women's welfare program participation?’, *Southwestern Economic Review,* 34, 145-159. |
| [25] | Lee, K. H. and Yoon, D. P. (2012), ‘A comparison of sanctions in African American and white TANF leavers’, *Journal of Evidence-Based Social Work*, 9, 396-413. |
| [26] | Lindhorst, T. and Mancoske R. J. (2006), ‘The social and economic impact of sanctions and time limits on recipients of Temporary Assistance to Needy Families’, *Journal of Sociology and Social Welfare*, 33, 1, 93-114. |
| [27] | Lindhorst, T., Mancoske, R. J. and Kemp, A. A. (2000), ‘Is welfare reform working? A study of the effects of sanctions on families receiving Temporary Assistance to Needy Families’, *Journal of Sociology and Social Welfare*, 27, 4, 185-201. |
| [28] | Lissenburgh, S. (2004), ‘New deal option effects on employment entry and unemployment exit. An evaluation using propensity score matching’, *International Journal of Manpower*, 25, 5, 411-430. |
| [29] | Moffitt, R. (2000), ‘The diversity of welfare leavers’, Policy Brief 00-01, Baltimore: Johns Hopkins University, <http://web.jhu.edu/threecitystudy/Publications/index_old.html> [accessed 01.12.2019]. |

TABLE A5. Continued

| Study no. [#] | Reference |
| --- | --- |
|  |  |
| [30] | Moore, Q., Wood, R. G. and Rangarajan, A. (2012), ‘The dynamics of women disconnected from employment and welfare’, *Social Service Review*, 86, 1, 93-118. |
| [31] | Müller, K.-U. and Steiner, V. (2008), ‘Imposed benefit sanctions and the unemployment-to-employment transition: the German experience’, Institute for the Study of Labor (IZA) Discussion Paper 3483, Bonn: IZA, <https://www.iza.org/publications/dp/3483/imposed-benefit-sanctions-and-the-unemployment-to-employment-transition-the-german-experience> [accessed 01.12.2019]. |
| [32] | Oggins, J. and Fleming, A. (2001), ‘Welfare reform sanctions and financial strain in a food-pantry sample‘, *Journal of Sociology and Social Welfare*, 28, 2, 101-124. |
| [33] | Ovwigho, P. C., Kolupanowich, N. J., Hetling, A. and Born, C. E. (2011), ‘Lost leavers: uncovering the circumstances of those without welfare and without work’, *Families in Society: The Journal of Contemporary Social Services*, 92, 4, 397-404. |
| [34] | Peck, L. (2007), ‘What are the effects of welfare sanction policies? Or, using propensity scores as a subgroup indicator to learn more from social experiments’, *American Journal of Evaluation*, 28, 3, 256-274. |
| [35] | Pingle, J. F. (2005), ‘Welfare, intergenerational cohabitation penalties, and single mothers' employment’, *Review of Economics of the Household*, 3, 123-144. |
| [36] | Qureshi, A. (2013), ‘The effect of punitive sanctions on the transition rate from welfare to work: evidence from Denmark’, *Nationalokonomisk Tidsskrift*, 151, 2, 225-246. |
| [37] | Reeves, A. (2017), ‘Does sanctioning disabled claimants of unemployment insurance increase labour market inactivity? An analysis of 346 British local authorities between 2009 and 2014’, *Journal of Poverty and Social Justice*, 25, 2, 129-146. |
| [38] | Røed, K. and Skogstrøm, J. F. (2014), ‘Unemployment insurance and entrepreneurship’, *Labour*, 28, 4, 430-448. |
| [39] | Schneider, J. (2008), ‘The effect of unemployment benefit II sanctions on reservation wages’, IAB Discussion Paper 19/2008, Nuremberg: Institute for Employment Research (IAB), <https://www.iab.de/966/section.aspx/Publikation/k080429n01> [accessed 01.12.2019]. |
| [40] | Schram, S. F., Fording, R. C. and Soss, J. (2008), ‘Neo-liberal poverty governance: race, place and the punitive turn in US welfare policy’, *Cambridge Journal of Regions, Economy and Society*, 1, 1, 17-36. |
| [41] | Snarr, H. W. (2013), ‘Was it the economy or reform that precipitated  the steep decline in the US welfare caseload?’, *Applied Economics*, 45, 4, 525-540. |
| [42] | Svarer, M. (2011), ‘The effect of sanctions on exit from unemployment: evidence from Denmark’, *Economica*, 78, 312, 751-778. |
| [43] | Swenson, T., White, S. and Murdock, S. (2002), ‘Time limit and sanction effects of the Texas TANF Waiver’, *Southern Rural Sociology*, 18, 1, 82-110. |
| [44] | van den Berg, G. J. and Vikström, J. (2014), ‘Monitoring job offer decisions, punishments, exit to work, and job quality’, *Scandinavian Journal of Economics*, 116, 2, 284-334. |
| [45] | van den Berg, G. J., Hofmann, B. and Uhlendorff, A. (2019), ‘Evaluating vacancy referrals and the role of sanctions and sickness absence’, *The Economic Journal*, 129, 3292-3322. |
| [46] | van den Berg, G. J., Uhlendorff, A. and Wolff, J. (2014), ‘Sanctions for young welfare recipients’, *Nordic Economic Policy Review*, 1, 177-208. |

TABLE A5. Continued

| Study no. [#] | Reference |
| --- | --- |
|  |  |
| [47] | van den Berg, G. J., Uhlendorff, A. and Wolff, J. (2017), ‘Under heavy pressure: intense monitoring and accumulation of sanctions for young welfare recipients in Germany’, IZA Discussion Paper 10730, Bonn: Institute of Labor Economics (IZA),  <https://www.iza.org/publications/dp/10730/under-heavy-pressure-intense-monitoring-and-accumulation-of-sanctions-for-young-welfare-recipients-in-germany> [accessed 01.12.2019]. |
| [48] | van den Berg, G. J., van der Klaauw, B. and van Ours, J. C. (2004), ‘Punitive sanctions and the transition rate from welfare to work’, *Journal of Labor Economics*, 22, 1, 211-241. |
| [49] | van der Klaauw, B. and van Ours, J. C. (2013), ‘Carrot and stick: how re-employment bonuses and benefit sanctions affect exit rates from welfare’, *Journal of Applied Econometrics*, 28, 2, 275-296. |
| [50] | Wu, C.-F. (2008), ‘Severity, timing, and duration of welfare sanctions and the economic well-being of TANF families with children’*, Children and Youth Services Review*, 30, 1, 26-44. |
| [51] | Wu, C.-F., Cancian, M. and Wallace, G. (2014), ‘The effect of welfare sanctions on TANF exits and employment’, *Children and Youth Services Review*, 36, 1-14. |
| [52] | Yu, H. (2001), ‘Welfare reform and caseload reductions’, *International Journal of Social Economics*, 28, 4, 383-343. |
| [53] | Arni P. and Schiprowski A. (2016), ‘Strengthening enforcement in unemployment insurance: a natural experiment’, IZA Discussion Paper no. 10353, Bonn: Institute for the Study of Labor (IZA), <https://www.iza.org/en/publications/dp/10353/strengthening-enforcement-in-unemployment-insurance-a-natural-experiment> [accessed 01.12.2019]. |
| [54] | Arni, P. and Schiprowski, A. (2019), ‘Job search requirements, effort provision and labour market outcomes’, *Journal of Public Economics*, 169, 65-88. |
| [55] | Boockmann, B., Thomsen, S. L. and Walter, T. (2014), ‘Intensifying the use of benefit sanctions: an effective tool to increase employment?’, *IZA Journal of Labor Policy*, 3, 21, 1-19. |
| [56] | Cockx, B. and Dejemeppe, M. (2007), ‘Is the notification of monitoring a threat to the unemployed? A regression discontinuity approach’, IZA Discussion Paper no. 2854, Bonn: Institute for the Study of Labor (IZA), <http://ftp.iza.org/dp2854.pdf> [accessed 01.12.2019]. |
| [57] | Cockx, B. and Dejemeppe, M. (2012), ‘Monitoring job search effort: an evaluation based on a regression discontinuity design’, *Labour Economics*, 19, 5, 729-737. |
| [58] | Danielson, C. and Klerman, J. (2008), ‘Did welfare reform cause the caseload decline?’, *The Social Service Review*, 82, 4, 703-730. |
| [59] | Fording, R. C., Schram, S. F. and Soss, J. (2013), ‘Do welfare sanctions help or hurt the poor? Estimating the causal effect of sanctioning on client earnings’, *The Social Service Review*, 87, 4, 641-676. |
| [60] | Moffitt, R. (2003), ‘The role of nonfinancial factors in exit and entry in the TANF program’, *Journal of Human Resources*, 38, 1221-1254. |
| [61] | National Audit Office (NAO) (2016b), ‘Benefit sanctions: detailed methodology’, Report HC 628, London: National Audit Office, <https://www.nao.org.uk/report/benefit-sanctions/> [accessed 01.12.2020]. |
| [62] | Taulbut M., Mackay D. F. and McCartney G. (2018), ’Job Seeker’s Allowance (JSA) benefit sanctions and labour market outcomes in Britain, 2001–2014’, *Cambridge Journal of Economics*, 42, 5, 1417-1434. |

TABLE A5. Continued

| Study no. [#] | Reference |
| --- | --- |
|  |  |
| [63] | Knab, J. T., Bos, J. M., Friedlander, D. and Weissman, J. W. (2000), ‘Do mandates matter? The effects of a mandate to enter a welfare-to-work program’, New York: Manpower Demonstration Research Corporation (MDRC), <https://www.mdrc.org/publication/do-mandates-matter> [accessed 01.12.2020]. |
| [64] | Michaelidis, M. and Mueser, P. (2018), ‘Are reemployment services effective? Experimental evidence from the Great Recession’, *Journal of Policy Analysis and Management*, 37, 3, 546-570. |
| [65] | Micklewright, J. and Nagy, G. (2010), ‘The effect of monitoring unemployment insurance recipients on unemployment duration: Evidence from a field experiment’, *Labour Economics*, 17, 180-187. |
| [66] | Olson, J. A., Schexnayder, D. T. and O'Shea, D. P. (1997), ‘Participation patterns and program impacts of Hawaii's JOBS WORKS! Demonstration Project’, Austin: Texas University, Lyndon B. Johnson School of Public Affairs, <https://eric.ed.gov/?id=ED416421> [accessed 01.12.2020]. |
| [67] | Riccio J. and Hasenfeld Y. (1996), ’Enforcing a participation mandate in a welfare-to-work program’, *Social Service Review*, 70, 4, 516-42. |
| [68] | Scrivener, S. and Walter, J. (2001), ‘Evaluating two approaches to case management: implementation, participation patterns, costs, and three-year impacts of the Columbus Welfare-to-Work Program’, New York: Manpower Demonstration Research Corporation (MDRC), <https://eric.ed.gov/?id=ED455442> [accessed 01.12.2020]. |
| [69] | Baltagi, B. H. and Yen, Y.-F. (2016), ‘Welfare reform and children’s health’, *Health Economics*, 25, 277-291. |
| [70] | Casey, P., Goolsby, S., Berkowitz, C., Frank, D., Cook, J., Cutts, D., Black, M. M., Zaldivar, N., Levenson, S., Heeren, T. and Meyers, A. (2004), ‘Maternal depression, changing public assistance, food security, and child health status’, *Pediatrics*, 113, 2, 298-304. |
| [71] | Chase-Lansdale, P. L., Coley, R. L., Lohman, B. J. and Pittman, L. D. (2002), ‘Welfare Reform: What about the Children? Welfare, Children & Families: A Three-City Study. Policy Brief’, Report No-02-01, Baltimore: Johns Hopkins University, <http://web.jhu.edu/threecitystudy/Publications/index_old.html> [accessed 01.12.2020]. |
| [72] | Cook, J. T., Frank, D. A., Berkowitz, C., Black, M. M., Casey, P. H., Cutts, D. B., Meyers, A. F., Zaldivar, N., Skalicky, A., Levenson, S. and Heeren, T. (2002), ‘Welfare reform and the health of young children: A sentinel survey in 6 US cities’, *Archives of Pediatrics and Adolescent Medicine*, 156, 678-684. |
| [73] | Davis, O. (2019), ‘What is the relationship between benefit conditionality and mental health? Evidence from the United States on TANF policies’, *Journal of Social Policy*, 48, 2, 249-269. |
| [74] | Dunifon, R., Hynes, K. and Peters, E. H. (2009), ‘State welfare policies and children's living arrangements’, *Social Service Review*, 83, 3, 351-388. |
| [75] | Eardley, T. (2006), ‘The Impact of breaching and financial penalties on income support recipients’, *The Economic and Labour Relations Review*, 17, 1, 79-105. |
| [76] | Fitzgerald, J. M. and Ribar, D. C. (2004), ’Welfare reform and female headship’, *Demography*, 41, 2, 189-212. |
| [77] | Hunter, T. and Santhiveeran, J. (2005), ‘Experiences of material hardships among TANF leavers’, *Journal of Family Social Work*, 9, 1, 1-16. |
| [78] | Kalil, A., Seefeldt, K. S. and Wang, H.-C. (2002), ‘Sanctions and material hardship under TANF’, *Social Service Review*, 76, 4, 642-662. |

TABLE A5. Continued

| Study no. [#] | Reference |
| --- | --- |
|  |  |
| [79] | Larson, A. M., Singh, S. and Lewis, C. (2011), ’Sanctions and education outcomes for children in TANF families’, *Child & Youth Services*, 32, 2, 180-189. |
| [80] | Livermore, M., Powers, R. S., Lim, Y. and Davis, B. C. (2015), ‘Predicting material hardship among former welfare-to-work participants: an income- and resource-packaging model’, *Journal of Social Service Research*, 41, 2, 158-171. |
| [81] | Lohman, B. J., Pittman, L. D., Coley, R. L. and Chase-Lansdale, P. L. (2004), ‘Welfare history, sanctions, and developmental outcomes among low-income children and youth’, *Social Service Review*, 78, 1, 41-73. |
| [82] | Loopstra, R., Fledderjohann, J., Reeves, A. and Stuckler, D. (2018) ‘Impact of welfare benefit sanctioning on food insecurity: a dynamic cross-area study of food bank usage in the UK’, *Journal of Social Policy*, 47, 3, 437-457. |
| [83] | Loopstra, R., Reeves, A., Taylor-Robinson, D., Barr, B., McKee, M. and Stuckler, D. (2015), ‘Austerity, sanctions, and the rise of food banks in the UK’, *British Medical Journal*, 360, h1775, 8 April. |
| [84] | Ovwigho, P. C., Leavitt, K. L. and Born, C. E. (2003), ‘Risk factors of child abuse and neglect among former TANF families: do later leavers experience greater risk?’, *Children and Youth Services Review*, 25, 1/2, 139-163. |
| [85] | Paxson, C. and Waldfogel, J. (2003), ‘Welfare reforms, family resources, and child maltreatment’, *Journal of Policy Analysis and Management*, 22, 1, 85-113. |
| [86] | Reeves, A. and Loopstra, R. (2017),’'Set up to fail'? How welfare conditionality undermines citizenship for vulnerable groups’, *Social Policy and Society*, 16, 2, 327-338. |
| [87] | Reichman, N. E., Teitler, J. O. and Curtis, M. A. (2005), ‘TANF sanctioning and hardship’, *Social Service Review*, 79, 2, 215-236. |
| [88] | Rodgers, H. R., Payne, L. Jr. and Chervachidze, S. (2006), ‘State poverty rates: do the new welfare policies make a difference?’, *Review of Policy Research*, 33, 3, 657-679. |
| [89] | Ryan, S., Manlove, J. and Hofferth, S. L. (2006), ‘State-level welfare policies and nonmarital subsequent childbearing’, *Population Research and Policy Review*, 25, 1, 103-126. |
| [90] | Slack, K. S., Lee, B. J. and Berger, L. M. (2007), ‘Do welfare sanctions increase child protection system involvement? A cautious answer’, *Social Service Review*, 81, 2, 207-228. |
| [91] | Acs, G. and Nelson, S. (2004), ‘Changes in living arrangements during the late 1990s: do welfare policies matter?’, *Journal of Policy Analysis and Management*, 23, 2, 273-290. |
| [92] | Machin, S. and Marie, O. (2006), ‘Crime and benefit sanctions’, *Portuguese Economic Journal*, 5, 149–165. |
| [93] | Wang, J. S.-H. (2015), ‘TANF coverage, state TANF requirement stringencies, and child well-being’, *Children and Youth Services Review*, 53, 121-129. |
| [94] | Fein, D. J. and Lee, W. L. (2003), ‘The impacts of welfare reform on child maltreatment in Delaware’, *Children and Youth Services Review*, 25, 1/2, 83-111. |
|  |  |
